# Guidelines for Neuroprognostication in Comatose Adult Survivors of Cardiac Arrest

**DOI:** 10.1007/s12028-023-01688-3

**Published:** 2023-03-22

**Authors:** Venkatakrishna Rajajee, Susanne Muehlschlegel, Katja E. Wartenberg, Sheila A. Alexander, Katharina M. Busl, Sherry H. Y. Chou, Claire J. Creutzfeldt, Gabriel V. Fontaine, Herbert Fried, Sara E. Hocker, David Y. Hwang, Keri S. Kim, Dominik Madzar, Dea Mahanes, Shraddha Mainali, Juergen Meixensberger, Felipe Montellano, Oliver W. Sakowitz, Christian Weimar, Thomas Westermaier, Panayiotis N. Varelas

**Affiliations:** 1 Departments of Neurology and Neurosurgery, 3552 Taubman Health Care Center, SPC 5338, University of Michigan, 1500 E. Medical Center Drive, Ann Arbor, MI 48109-5338 USA; 2 Departments of Neurology, Anesthesiology, and Surgery, University of Massachusetts Chan Medical School, Worcester, MA USA; 3 Department of Neurology, University of Leipzig, Leipzig, Germany; 4 School of Nursing, University of Pittsburgh, Pittsburgh, PA USA; 5 Departments of Neurology and Neurosurgery, College of Medicine, University of Florida, Gainesville, FL USA; 6 Department of Neurology, Northwestern University Feinberg School of Medicine, Chicago, IL USA; 7 Department of Neurology, University of Washington, Seattle, WA USA; 8 Departments of Pharmacy and Neurosciences, Intermountain Healthcare, Salt Lake City, UT USA; 9 Department of Neurosurgery, Denver Health Medical Center, Denver, CO USA; 10 Department of Neurology, Mayo Clinic, Rochester, MN USA; 11 Department of Neurology, University of North Carolina at Chapel Hill, Chapel Hill, NC USA; 12 Pharmacy Practice, University of Illinois, Chicago, IL USA; 13 Department of Neurology, University of Erlangen, Erlangen, Germany; 14 Departments of Neurology and Neurosurgery, University of Virginia Health, Charlottesville, VA USA; 15 Department of Neurology, Virginia Commonwealth University, Richmond, VA USA; 16 Department of Neurosurgery, University of Leipzig, Leipzig, Germany; 17 Department of Neurology, University of Wuerzburg, Würzburg, Germany; 18 Department of Neurosurgery, Neurosurgery Center Ludwigsburg-Heilbronn, Ludwigsburg, Germany; 19 Institute of Medical Informatics, Biometry, and Epidemiology, University Hospital Essen, Essen, Germany; 20 BDH-Clinic Elzach, Elzach, Germany; 21 Department of Neurosurgery, University of Wuerzburg, Würzburg, Germany; 22 Department of Neurology, Albany Medical College, Albany, NY USA

**Keywords:** Cardiac arrest, Prognosis, Functional status, Coma, Mortality

## Abstract

**Background:**

Among cardiac arrest survivors, about half remain comatose 72 h following return of spontaneous circulation (ROSC). Prognostication of poor neurological outcome in this population may result in withdrawal of life-sustaining therapy and death. The objective of this article is to provide recommendations on the reliability of select clinical predictors that serve as the basis of neuroprognostication and provide guidance to clinicians counseling surrogates of comatose cardiac arrest survivors.

**Methods:**

A narrative systematic review was completed using Grading of Recommendations Assessment, Development and Evaluation (GRADE) methodology. Candidate predictors, which included clinical variables and prediction models, were selected based on clinical relevance and the presence of an appropriate body of evidence. The Population, Intervention, Comparator, Outcome, Timing, Setting (PICOTS) question was framed as follows: “When counseling surrogates of comatose adult survivors of cardiac arrest, should [predictor, with time of assessment if appropriate] be considered a reliable predictor of poor functional outcome assessed at 3 months or later?” Additional full-text screening criteria were used to exclude small and lower-quality studies. Following construction of the evidence profile and summary of findings, recommendations were based on four GRADE criteria: quality of evidence, balance of desirable and undesirable consequences, values and preferences, and resource use. In addition, good practice recommendations addressed essential principles of neuroprognostication that could not be framed in PICOTS format.

**Results:**

Eleven candidate clinical variables and three prediction models were selected based on clinical relevance and the presence of an appropriate body of literature. A total of 72 articles met our eligibility criteria to guide recommendations. Good practice recommendations include waiting 72 h following ROSC/rewarming prior to neuroprognostication, avoiding sedation or other confounders, the use of multimodal assessment, and an extended period of observation for awakening in patients with an indeterminate prognosis, if consistent with goals of care. The bilateral absence of pupillary light response > 72 h from ROSC and the bilateral absence of N20 response on somatosensory evoked potential testing were identified as reliable predictors. Computed tomography or magnetic resonance imaging of the brain > 48 h from ROSC and electroencephalography > 72 h from ROSC were identified as moderately reliable predictors.

**Conclusions:**

These guidelines provide recommendations on the reliability of predictors of poor outcome in the context of counseling surrogates of comatose survivors of cardiac arrest and suggest broad principles of neuroprognostication. Few predictors were considered reliable or moderately reliable based on the available body of evidence.

**Supplementary Information:**

The online version contains supplementary material available at 10.1007/s12028-023-01688-3.

## Introduction

The Cardiac Arrest Registry to Enhance Survival (CARES) 2020 report estimated that the crude incidence of nontraumatic out-of-hospital cardiac arrests (OHCAs) was 88.8 per 100,000 in 2020, greater than in each of the previous 3 years. Of 127,376 OHCAs with EMS response reported to CARES in 2020, 24% of patients survived to hospital admission and 9% of patients survived to hospital discharge. Of the patients who survived to hospital discharge, 79% had a good neurological outcome at discharge [1]. The American Heart Association (AHA) reported an incidence of adult in-hospital cardiac arrest (IHCA) of 10.16 per 1,000 hospital admissions in the 2019 Get With The Guidelines (GWTG) database [2]. Of 28,012 patients with IHCA in the GWTG database, 27% survived to discharge, and 80% of survivors had a good neurological outcome at discharge. Prognostication of long-term neurological outcome is relevant in survivors of cardiac arrest who remain comatose following return of spontaneous circulation (ROSC). More than 80% of patients with OHCA who achieve ROSC will be comatose an hour after, and about half will remain comatose 72 h following ROSC (or 72 h from rewarming when hypothermia is used) [3, 4]. In the United States, withdrawal of life-sustaining therapy (WLST) remains the most common cause of death following cardiac arrest, occurring in 40–80% of comatose survivors [5, 6]. It is likely that in these cases, WLST is preceded by prognostication of poor outcome by a clinician. Because the overwhelming majority of patients who undergo WLST after cardiac arrest will die [7], it is of critical importance that prognostication be performed accurately on the basis of appropriately validated predictors. Validation of predictors of outcome in comatose survivors of cardiac arrest is challenging. A 2019 scientific statement from the AHA reviewed the specific challenges involved with the conduct of research in this population and recommended standards for future studies [8]. One of the most important challenges identified was the self-fulfilling prophecy. Because this risk of bias, to varying degrees, is inherent in most studies of neuroprognostication following cardiac arrest, an element of uncertainty is unavoidable even with predictors identified as reliable. There is, therefore, inherent risk with the formulation of guidelines for neuroprognostication in comatose patients dependent on life support. However, prognostication during counseling is both essential and inevitable and occurs routinely in intensive care units (ICUs) worldwide. The objective of these guidelines from the Neurocritical Care Society and Deutsche Gesellschaft für Neurointensivmedizin is to ensure that such prognostication and counseling is performed on the basis of the most reliable predictors available rather than the arbitrary criteria clinicians may use in the absence of all guidance.

### Scope, Purpose, and Target Audience

The scope of these Grading of Recommendations Assessment, Development and Evaluation (GRADE) guidelines is the prognostication of neurological outcome in adult survivors of nontraumatic OHCA and IHCA who remain comatose following ROSC. The purpose of these guidelines is to provide evidence-based recommendations on the reliability of predictors of neurological outcome in comatose survivors of cardiac arrest to aid clinicians in formulating a prognosis. The target audience consists of clinicians responsible for such counseling.

### How to Use These Guidelines

These guidelines provide recommendations on the reliability of select demographic and clinical variables as well as prediction models when counseling families and surrogates of comatose survivors of cardiac arrest. We categorized these predictors as reliable, moderately reliable, or not reliable. We based this categorization on a GRADE-based assessment of certainty in the body of evidence, as well as effect size (quantification of predictor accuracy) across published studies, primarily the false positive rate (FPR), as shown in Table 1. Reliable predictors, for the purposes of these guidelines, may be used to formulate a prognosis when the appropriate clinical context is present in the absence of potential confounders. These are predictors with clear actionable thresholds or clinical/radiographic definitions and a low rate of error in prediction of poor outcomes and with at least moderate certainty in the body of evidence using GRADE criteria. When the prognosis is formulated on the basis of one or more reliable predictors, the clinician may describe the outcome as “very likely” during counseling. Given the inherent limitations in neuroprognostication research, the clinician must nevertheless acknowledge the presence of uncertainty, albeit low, in the prognosis. Moderately reliable individual predictors may be used for prognostication only when additional reliable or moderately reliable predictors are present, in addition to the appropriate clinical context as specified above. These are also predictors with clear, actionable thresholds or clinical/radiographic definitions and a low rate of error in prediction of poor outcomes but with lower certainty in the body of evidence using GRADE criteria, often a result of smaller studies that result in imprecision. When the prognosis is formulated on the basis of multiple moderately reliable predictors, the clinician may describe the outcome as “likely” during counseling but must acknowledge “substantial” uncertainty in the prognosis. Moderately reliable clinical prediction models that generate predicted probabilities of outcomes, in contrast, may be used for prognostication during counseling in the absence of other reliable or moderately reliable predictors. However, it is recommended that the clinician describe the predicted probability of the outcome as “an objective estimate only, subject to considerable uncertainty.” Although the panelists recognize that predictors that do not meet the criteria to be described as reliable or moderately reliable are often used by clinicians in formulating their subjective impressions of prognosis, they have nevertheless been deemed not reliable for the purposes of these guidelines and cannot be formally recommended for prognostication on their own. However, variables deemed not reliable may be a component of reliable or moderately reliable prediction models.

**Table 1:**
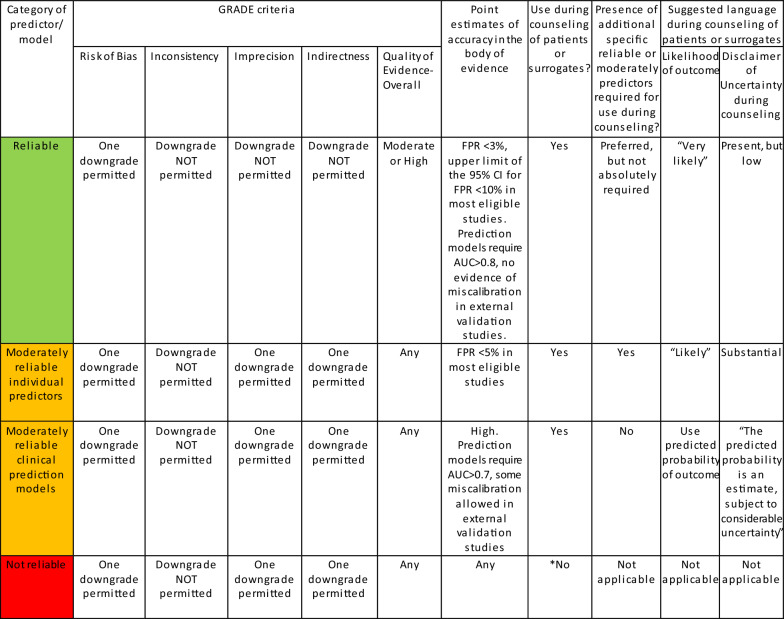
Reliable and moderately reliable predictors

*Many predictors designated “not reliable” are practically used by clinicians in formulating and communicating real-world subjective impressions of prognosis. The purpose of these guidelines is to identify predictors, if any, that meet reliable or moderately reliable criteria

## Methods

An in-depth description of the methodology used in these guidelines is available in the Supplementary Appendix 1.

### Selection of Guideline Questions

Candidate predictors were selected on the basis of clinical relevance and the presence of an appropriate body of literature. Candidate predictors and prediction models were considered “clinically relevant” if, in the subjective opinion of the content experts and guideline chairs, the predictor or components of the prediction models were (a) accessible to clinicians, although universal availability was not required, and (b) likely to be considered by clinicians while formulating a neurological prognosis for comatose survivors of cardiac arrest. Predictors addressed in prior cardiac arrest neuroprognostication guidelines were thought particularly likely to be considered by clinicians and therefore prioritized. An appropriate body of literature was considered present for any clinical variable that fulfilled two criteria: (1) evaluated in at least two published studies that included a minimum of 100 study participants and (2) established as an independent predictor in a multivariate analysis. An appropriate body of literature was considered present for clinical prediction models with at least one external validation study of at least 100 patients in addition to the initial report on development of the model (also with a minimum of 100 patients).

Based on these criteria, the following candidate predictors were selected:

Clinical variables:

1. Age
2. Initial cardiac rhythm (shockable vs. nonshockable)
3. Time to ROSC
4. Absent or extensor best motor response, assessed at least 72 h from ROSC (or 72 h from rewarming in patients treated with therapeutic hypothermia)
5. Bilateral absence of a pupillary light response assessed at least 72 h from ROSC
6. Myoclonus < 48 h from ROSC, without concomitant electroencephalography (EEG) assessment
7. Diffuse pattern (across vascular distributions in the bilateral anterior and posterior circulation, with involvement of cerebral cortex and deep gray matter) of loss of gray–white differentiation with sulcal effacement on noncontrast computed tomography (CT) imaging of the brain performed at least 48 h from ROSC
8. Diffuse pattern (across vascular distributions in the bilateral anterior and posterior circulation, with involvement of cerebral cortex and deep gray matter) of restricted diffusion on magnetic resonance imaging (MRI) of the brain performed between 2 and 7 days from ROSC
9. Suppressed or burst suppression background, with or without periodic discharges, on EEG performed at least 72 h from ROSC (or 72 h from rewarming in patients treated with therapeutic hypothermia) in the absence of sedation or other potential confounders
10. Bilateral absence of the N20 wave (with preservation of responses at Erb’s point and the cervical spine) on somatosensory evoked potential (SSEP) testing performed at least 48 h from ROSC
11. Serum level of neuron-specific enolase (NSE) measured < 72 h from ROSC

Clinical prediction models:

1. OHCA
2. Cardiac Arrest Hospital Prognosis (CAHP)
3. Good Outcome Following Attempted Resuscitation (GOFAR)

The Population, Intervention, Comparator, Outcome, Timing, Setting (PICOTS) question was then framed for the specific candidate predictors as follows: “When counseling surrogates of comatose adult survivors of cardiac arrest, should [predictor, with time of assessment if appropriate] be considered a reliable predictor of [outcome, with time frame of assessment]?”.

### Selection of Outcomes

The outcomes rated “critical” using the GRADE 1–9 scale were functional outcome (average rating 8.33) assessed at or beyond 3 months from ROSC, mortality (average rating 7.67) assessed at or beyond discharge, and cognitive outcome (average rating 7.33) assessed at or beyond 3 months from ROSC. However, no studies that included cognitive outcomes met other full-text screening criteria for the systematic review. Following the systematic review, the mortality outcome (particularly when assessed at discharge, the most common time point in the literature) was recognized to be inseparable from WLST, which accounts for up to 80% of deaths in this clinical setting [5, 6, 8, 9]. Although the panel did provide recommendations for predictors of mortality, available in Supplementary Appendix 2, the body of evidence for the prediction of all-cause mortality was thought to be compromised by an unacceptably high risk of bias from the self-fulfilling prophecy and not reflect the probability of death when life support measures are used indefinitely and in their entirety. The body of evidence for predictors of progression to death by neurological criteria or death in the absence of WLST was considered insufficient to serve as the basis for recommendations. Therefore, the primary focus of recommendations in these guidelines will be the prediction of long-term functional outcome.

Neuroprognostication in these guidelines is primarily focused on the prediction of poor outcomes, reflecting the overwhelming majority of research in comatose survivors of cardiac arrest. More recent publications have examined the ability to predict good outcomes in this population [10]. Functional outcome assessment of comatose survivors of cardiac arrest in the published literature has overwhelmingly been performed with the Cerebral Performance Category (CPC) scale (Supplementary Appendix 3) [11, 12]. The CPC is typically dichotomized at the ability to perform activities of daily living or work in a sheltered environment (CPC 1–2 vs. 3–5). Following this convention, for the purposes of this systematic review, a poor functional outcome was defined as severe disability, a minimally conscious state or a vegetative/unresponsive-wakeful state. Importantly, this definition of poor outcome is focused on recovery of functional ability and not on recovery of responsiveness. Although recovery of responsiveness in patients with long-term disorders of consciousness may be seen many months or years following hospital discharge, most of these patients have severe, persistent disability [13, 14]. Additionally, because this definition of poor outcome is focused on recovery of functional ability, a distinction between a minimally conscious and chronic vegetative/unresponsive-wakeful state is not relevant to these guidelines. Severe disability was defined as the equivalent of CPC > 2: the inability to perform activities of daily living or work in a sheltered environment. Several studies used the modified Rankin scale (Supplementary Appendix 3), which was developed for the assessment of outcome in cerebrovascular disease [15, 16] and was considered an appropriate alternative to the CPC. Other functional outcome scales that incorporated the ability to perform activities of daily living were also considered acceptable. The assessment of functional outcome at 3 months or later is consistent with recommendations of the AHA consensus statement on primary outcomes for resuscitation science studies [17], as well as the AHA standards for neuroprognostication research following cardiac arrest [8]. There is evidence that a significant proportion of patients demonstrate an improvement in functional outcomes following discharge [18, 19]. In one study, 50% of patients progressed from poor to good functional outcome between 1 and 3 months [20]. In addition, the ability to work in a sheltered environment, travel by public transportation, or prepare food, all of which are inherent to the CPC score, cannot be adequately assessed in the inpatient setting. Although a longer duration from time of injury to outcome assessment is ideal to capture the entirety of functional recovery, this may result in loss to follow-up. Significant loss to follow-up in observational studies may result in a selection bias based on the patients most likely to respond or return to the index hospital for further medical care.

### Systematic Review Methodology

**Fig. 1:**
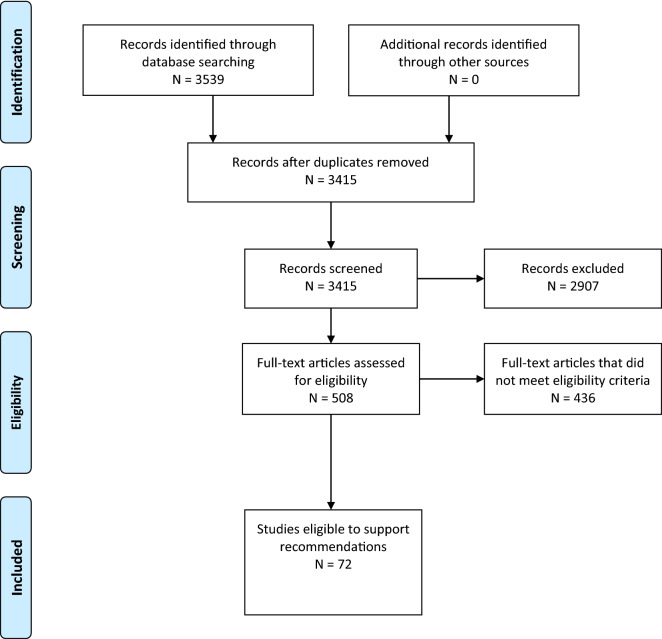
PRISMA 2009 flow diagram- systematic review: neuroprognostication in adult comatose survivors of cardiac arrest

An in-depth description of systematic review methodology for these guidelines is in Supplementary Appendix 1. The librarian search string used for this systematic review is in Supplementary Appendix 4 and the Preferred Reporting Items for Systematic Reviews and Meta-Analyses flow diagram in Fig. 1. Full-text screening was performed with the following exclusion criteria: sample size less than 100, studies focused on a highly selected subgroup (such as traumatic cardiac arrest), studies of predictors not established as independent with multivariate analysis, studies focused on a genetic polymorphism as a predictor, and studies of clinical prediction models that did not report model discrimination. Studies of laboratory biomarkers were included only if the biomarker was considered clinically relevant and had been evaluated in more than one published study that met other criteria.

Studies with no restrictions on WLST and likely incorporation of predictors under investigation into clinical neuroprognostication during the course of the study were considered to have a high risk of bias from the self-fulfilling prophecy. Studies that included a systematic restriction of WLST for at least 72 h and those that blinded clinicians to the predictor under investigation were considered to have a moderate risk of bias in this domain. Studies from countries with restrictions or cultural limitations on withdrawal of life support, including from East Asia, were judged to have a lower risk of bias from the self-fulfilling prophecy [21]. However, withholding escalation of therapy, including cardiopulmonary resuscitation in the event of recurrent cardiac arrest, is relatively common in these settings [21]. Studies from these settings with a mortality outcome that did not include specific restrictions on withholding escalation of care were therefore judged to have a moderate risk of bias from the self-fulfilling prophecy.

A summary of individual studies of predictors is in Supplementary Appendix 5. The GRADE evidence profile (EP) and summary of findings (SoF) table for predictors of functional outcome is in Table 2, and the EP/SoF table for predictors of mortality is in Supplementary Appendix 2.

**Table 2:** GRADE evidence profile/summary of findings table: neuroprognostication, cardiac arrest

Outcome	Predictor	Quality of evidence					Summary of findings (narrative of effect size)
		Risk of bias	Inconsistency	Indirectness	Imprecision	Quality of evidence, summary	
Functional outcome	Age	↓	↓			Low	Point estimate of odds ratio for poor outcome 0.98–2.90
Functional outcome	Cardiac rhythm- nonshockable	↓			↓	Low	Point estimate of odds ratio for poor outcome 3.89–13.46 Point estimate of odds ratio for good outcome 0.09–0.36. False positive rate 13–40%
Functional outcome	Time to return of spontaneous circulation	↓				Moderate	Point estimate of odds ratio for poor outcome 1.03–1.05 Point estimate of odds ratio for good outcome 0.79–0.96. False positive rate 24–32%
Functional outcome	Neurological examination ≥ 72 h from ROSC—Bilateral absence of the pupillary reflex	↓	Inconsistency explained by time of assessment			Moderate	Point estimate of odds ratio for poor outcome 3.8. False positive rate 0–5% (upper limit of 95% confidence interval up to 0–12%)
Functional outcome	Neurological examination ≥ 72 h from ROSC—Bilateral absence of the corneal reflex	↓	Inconsistency explained by time of assessment			Moderate	Point estimate of OR is 5.63–6.643. False positive rate 0–16% for the corneal reflex alone
Functional outcome	Neurological examination ≥ 72 h from ROSC- Motor response no better than extension	↓	↓			Low	Point estimate of odds ratio for good outcome 0.40–0.83. False positive rate 0–30%
Functional outcome	Myoclonus ≤ 48 h from ROSC	↓↓			↓	Very low	Sample sizes generally too small for a meaningful OR. False positive rate 0–10% but large risk of bias in prognostic factor measurement
Functional outcome	Computed Tomography (CT) ≥ 72 h from ROSC- diffuse loss of grey-white differentiation with sulcal effacement	↓		↓	↓	Very low	Sensitivity 1–37%, False positive rate 0–3% but with wide confidence intervals
Functional outcome	Magnetic Resonance Imaging (MRI)- with diffuse pattern of restricted diffusion 2–7 days from ROSC	↓		↓	↓	Very low	AUC 0.83–0.94. Sensitivity 33–93%, False positive rates 0–6% but with wide confidence intervals
Functional outcome	Electroencephalography (EEG) ≥ 72 h from ROSC with suppressed or burst suppression background, with or without periodic discharges	↓			↓	Low	Sensitivity 30–64%, False positive rate 0–5% but with wide confidence intervals
Functional outcome	Somatosensory evoked potential (SSEP)	↓				Moderate	False positive rate point estimate 0–3%, upper limit of 95% CI 0–7%. Sensitivity 31–71%. Point estimate of AUC 0.65–0.86
Functional outcome	Neuron Specific Enolase (NSE)	↓	Inconsistency present, but mostly explained by the use of different thresholds			Moderate	False positive rate point estimate 0–42%, Sensitivity 61–92% depending on threshold and other factors. Point estimate of AUC 0.78–0.91. Odds ratio 1.04–37.47
Functional outcome	Out of Hospital Cardiac Arrest (OHCA) prediction model	↓				Moderate	Insufficient evidence. AUC 0.57–0.86. OHCA score > 60: Sensitivity 2–25%, Specificity 100% for poor functional outcome. Calibration not reported for functional outcome at 3 months or beyond
Functional outcome	Cardiac Arrest Hospital Prognosis (CAHP) prediction model	↓				Moderate	Insufficient evidence. Single study with functional outcome assessed 3 months or beyond- AUC 0.8, sensitivity 5% and specificity 100%, calibration not reported
Functional outcome	Good Outcome Following Attempted Resuscitation (GOFAR) prediction model	↓		↓		Low	Insufficient evidence- no studies with assessment of functional outcome at 3 months or beyond. One study reports miscalibration with systematic underestimation of neurologically intact survival

### Effect Size (Predictor Accuracy)

Predictor accuracy is often described using measures such as the odds ratio (OR), which measures the relative probability of the outcome when the predictor is present compared to the probability of the outcome in the absence of the predictor. In the context of counseling families of comatose survivors of cardiac arrest on the likelihood of a poor outcome, the single most important measure of accuracy of individual clinical variables may be the FPR, the proportion of patients with a good outcome in whom the predictor was observed. FPR = [false positives/(false positives + true negatives)] = [1 – specificity]. The sensitivity of the predictor, or the proportion of patients who suffer a poor outcome in whom the predictor is observed, is also useful. Predictors with lower sensitivity, such as the bilateral absence of both the pupillary light response and corneal reflex, typically have lower prevalence in comatose cardiac arrest survivors, whereas the rate of poor outcome may be high. Clinical prediction model performance is evaluated using measures of model discrimination and calibration, as described in Supplementary Appendix 1 [22].

### Evidence to Recommendation Criteria

1. Quality of evidence/certainty in the evidence and effect size: For the purposes of these guidelines, predictors described as “reliable” have both a higher overall certainty in the evidence and greater effect size than “moderately reliable” predictors (Table 1). For “reliable” individual predictors, one downgrade was permitted for risk of bias, but none for inconsistency, imprecision or indirectness, and the overall quality of evidence was high or moderate. Reliable predictors were required to have a point estimate of the FPR ≤ 3% and upper limit of the 95% confidence interval (CI) for FPR ≤ 10% in most eligible studies. “Reliable” prediction models were required to demonstrate an area under the receiver operating curve (AUC) of > 0.8 and no evidence of miscalibration in external validation studies that reported calibration. Single downgrades in each of the domains of risk of bias, imprecision, and indirectness were permitted for “moderately reliable” predictors, but a downgrade for inconsistency was not. Moderately reliable predictors were required to have a point estimate of FPR ≤ 5%. No upper limit of the 95% CI was specified for “moderately reliable” predictors because imprecision was permitted. In addition, “moderately reliable” prediction models were required to demonstrate an AUC > 0.7, and some miscalibration in some external populations was allowed. Predictors that did not fit “reliable” or “moderately reliable” criteria were classified as “not reliable.”
2. Balance of desirable and undesirable consequences: An accurate prediction of poor outcome is expected to result in grief, a sense of loss and anxiety about the future. However, a desirable consequence of accurate prediction of a poor outcome is the ability of surrogates and the clinical team to align goals of care to the perceived wishes of the comatose survivor. Potential desirable consequences for family and surrogates in this situation include greater certainty in the course of events, a sense of closure, and catharsis from respecting the patient’s wishes. However, inaccurate prediction of a poor outcome (i.e., a false positive prediction of poor outcome) may lead to the undesirable consequence of withdrawal of life support in an individual who would otherwise have made a meaningful recovery. Because the withdrawal of life support measures almost always leads to death in comatose survivors of cardiac arrest, the undesirable consequence of an inaccurate prediction of poor outcome was a primary concern during discussions on the reliability of a predictor. Other potential undesirable consequences of predictor assessment include the risk of events such as loss of airway, hemodynamic instability, inadvertent removal of catheters, and cardiac arrest during transport of a critically ill patient for tests such as brain imaging.
3. Values and preferences: The panel, including the patient representative, was in agreement that most individuals, as well as their surrogates, would likely consider an inaccurate prediction of poor outcome that led to the death of a patient who might otherwise have had a reasonable recovery to be far more undesirable than a prolonged period of uncertainty in the outcome. Therefore, a high certainty in the evidence of predictor or prediction model accuracy was necessary to recommend consideration when counseling families and surrogates on prognosis in this context.
4. Resource use: Resource use varied across predictors and models. Whereas some predictors, such as the assessment of the pupillary light response or best motor response, require minimal expenditure of resources, other predictors, such as MRI and measurement of NSE levels, do involve significant expenditure of resources in the cost of the diagnostic/prognostic test itself, as well as in transport of a critically ill patient. Some diagnostic tests, such as MRI, continuous EEG, and especially NSE, are not as widely available as others, such as routine < 1 h EEG and CT. However, an accurate prediction of poor outcome may avoid extended use of resources over days to years in patients destined to suffer a poor outcome. The use of resources was therefore thought to favor consideration of a predictor or prediction model during prognostication when confidence in its predictive accuracy was high. This was thought to especially be true in low- and middle-income settings, where extended out-of-pocket expenditure is associated with poverty [23, 24]. However, in situations where goals of care have previously been established and are unlikely to change, resource use involved with performance of the test should be considered and expensive tests not expected to alter the treatment plan should be avoided.

A summary of all recommendations for the prediction of long-term functional outcome is in Table 3. Recommendations for the prediction of mortality are in Supplementary Appendix 2.

**Table 3:** Summary of recommendations: neuroprognostication following cardiac arrest: good practice statements and predictors of long-term functional outcome

**Good practice statements**
We recommend deferral of assessment of the neurological prognosis of comatose survivors of cardiac arrest for at least 72 h following ROSC in patients not treated with therapeutic hypothermia (goal temperature < 36.5 °C), and at least 72 h following rewarming in patients treated with hypothermia. However, persistence of coma beyond this period in the ICU must not be equated with a poor neurological prognosis (strong recommendation, evidence cannot be graded)
We recommend that assessment of the neurological prognosis of comatose survivors of cardiac arrest be performed in the absence of sedation or other potential confounders (strong recommendation, evidence cannot be graded)
We recommend that factors that impact overall prognosis- such as a poor baseline level of functioning, pre-existing illness associated with limited life-expectancy and multi-organ failure- be considered prior to, and distinct from, assessment of the neurological prognosis of comatose survivors of cardiac arrest (strong recommendation, evidence cannot be graded)
We recommend that assessment of the neurological prognosis of comatose survivors of cardiac arrest be multimodal, with consideration of the complete clinical scenario, and never based on a single variable (strong recommendation, evidence cannot be graded)
We recommend that in the absence of reliable (or multiple moderately reliable) predictors of outcome, surrogates of cardiac arrest survivors who remain comatose at the time of neuroprognostication be counseled that the likelihood, extent and time course of neurological recovery is uncertain. Surrogates should also be counseled that the timeline of any functional recovery that does occur may extend from several days to several months (strong recommendation, evidence cannot be graded)
We suggest an extended period of observation for signs of neurological recovery in comatose survivors of cardiac arrest with an indeterminate prognosis, if consistent with the goals of care as established through discussions with patient surrogates (strong recommendation, evidence cannot be graded)
**Predictors of functional outcome at 3 months or later**
**Age, cardiac rhythm, and time to return of spontaneous circulation**
When counseling surrogates of comatose survivors of cardiac arrest, we suggest the patient’s age alone not be considered a reliable predictor of poor functional outcome assessed at three months or later (weak recommendation; low quality evidence)
When counseling family members and/or surrogates of comatose survivors of cardiac arrest, we suggest the initial cardiac rhythm alone not be considered a reliable predictor of poor functional outcome assessed at three months or later (weak recommendation; low quality evidence)
When counseling family members and/or surrogates of comatose survivors of cardiac arrest, we suggest the time to return of spontaneous circulation (ROSC) alone not be considered a reliable predictor of poor functional outcome assessed at three months or later (weak recommendation; moderate quality evidence)
**Neurological examination**
When counseling family members and/or surrogates of comatose survivors of cardiac arrest, we suggest the bilateral absence of a pupillary light response, assessed at least 72 h from ROSC, be considered a reliable predictor of poor functional outcome assessed at three months or later. This recommendation is conditional on accurate assessment without confounding by medication, hypothermia or prior surgery, and an overall clinical picture consistent with severe, widespread neurological injury (weak recommendation; moderate quality evidence)
When counseling family members and/or surrogates of comatose survivors of cardiac arrest, we suggest the bilateral absence of the corneal reflex alone, assessed at least 72 h from ROSC, not be considered a reliable predictor of poor functional outcome assessed at three months or later (weak recommendation; moderate quality evidence)
When counseling family members and/or surrogates of comatose survivors of cardiac arrest, we suggest that an absent or extensor best-motor response alone, assessed at least 72 h from ROSC (or 72 h from rewarming, in patients treated with therapeutic hypothermia) not be considered a reliable predictor of poor functional outcome assessed at three months or later (weak recommendation; low quality evidence)
When counseling family members and/or surrogates of comatose survivors of cardiac arrest, we suggest that the occurrence of myoclonus < 48 h from return of spontaneous circulation, in the absence of concomitant EEG evaluation, not be considered a reliable predictor of poor functional outcome assessed at three months or later (weak recommendation; very low quality of evidence)
**Brain imaging**
When counseling family members and/or surrogates of comatose survivors of cardiac arrest, we suggest that a diffuse pattern (across vascular distributions in the bilateral anterior and posterior circulation, with involvement of cerebral cortex and deep grey matter) of loss of grey–white differentiation with sulcal effacement on non-contrast computed tomography (CT) imaging of the brain performed at least 48 h from return of spontaneous circulation be considered a moderately reliable predictor of poor functional outcome assessed at three months or later (weak recommendation; very low quality evidence)
When counseling family members and/or surrogates of comatose survivors of cardiac arrest, we suggest that a diffuse pattern (across vascular distributions in the bilateral anterior and posterior circulation, with involvement of cerebral cortex and deep grey matter) of restricted diffusion on magnetic resonance imaging (MRI) of the brain performed between 2 and 7 days from ROSC be considered a moderately reliable predictor of poor functional outcome assessed at three months or later (weak recommendation; very low quality evidence)
**Electrodiagnostic**
When counseling family members and/or surrogates of comatose survivors of cardiac arrest, we suggest that a suppressed or burst suppression background, with or without periodic discharges, on EEG performed at least 72 h from ROSC (or 72 h from rewarming, in patients treated with therapeutic hypothermia) in the absence of sedation or other potential confounders such as hypothermia be considered a moderately reliable predictor of poor functional outcome assessed at three months or later (weak recommendation; low quality evidence)
When counseling family members or surrogates of comatose survivors of cardiac arrest, we suggest that the bilateral absence of the N20 wave, with preservation of responses at Erb’s point and the cervical spine, on somatosensory evoked potential (SSEP) testing performed at least 48 h from return of spontaneous circulation, be considered a reliable predictor of poor functional outcome assessed at three months or later. This recommendation is conditional on accurate measurement and interpretation of the SSEP, and an overall clinical picture consistent with severe, widespread neurological injury (weak recommendation; moderate quality evidence)
**Biomarkers**
When counseling surrogates of comatose survivors of cardiac arrest, we suggest that the serum level of neuron specific enolase (NSE) alone, measured ≤ 72 h from return of spontaneous circulation, not be considered a reliable predictor of poor functional outcome assessed at three months or later until a consistent threshold is validated (weak recommendation; moderate quality evidence)
**Prediction models**
Out of hospital cardiac arrest (OHCA): There is insufficient evidence for a recommendation
Cardiac Arrest Hospital Prognosis (CAHP): There is insufficient evidence for a recommendation
When counseling family members or surrogates of comatose survivors of in-hospital cardiac arrest, we suggest the Good Outcome Following Attempted Resuscitation (GOFAR) clinical prediction model alone not be considered a reliable predictor of poor functional outcome assessed at 3 months or later (weak recommendation; moderate quality evidence)

### Good Practice Statements

In accordance with recommendations of the GRADE network, these statements were considered by the panel to be actionable, supported by indirect evidence where appropriate, and essential to guide the practice of neuroprognostication [25]. The good clinical practice reflected in these statements lacked a meaningful body of direct supporting evidence (typically because of insufficient clinical equipoise) but was considered by the panel to be unequivocally beneficial.

### Good Practice Statement 1

We recommend deferral of assessment of the neurological prognosis of comatose survivors of cardiac arrest for at least 72 h following ROSC in patients not treated with therapeutic hypothermia (goal temperature < 36.5 °C) and at least 72 h following rewarming in patients treated with hypothermia. However, persistence of coma beyond this period in the ICU must not be equated with a poor neurological prognosis (strong recommendation, evidence cannot be graded).

### Rationale

The rationale for this recommendation is that the majority of patients who awaken from coma following cardiac arrest will do so within the recommended time frame, rendering neuroprognostication unnecessary [3, 5–7, 20, 26–32]. In the Targeted Temperature Management (TTM) clinical trial, 45% of patients in the hypothermia (33 °C) arm and 52% of patients in the normothermia arm (36 °C) woke from coma within 72 h of rewarming [4]. About 64–88% of patients not treated with hypothermia who awaken will do so within 72 h from ROSC, whereas 51–90% of patients treated with hypothermia who awaken will do so within 72 h from rewarming [4, 5, 20, 27–29, 31–39]. A further 10–15% of patients will suffer death by neurological criteria, and a similar percentage will die of cardiopulmonary instability in the first few days despite the use of life support measures, again rendering neuroprognostication unnecessary [4, 5, 40–42]. Note that some tests required for neuroprognostication, such as SSEP and imaging, can be performed beyond 48 (rather than 72) hours. Most, but not all, patients who awaken in the ICU will have a good long-term neurological recovery. There is an insufficient body of evidence to identify predictors of poor outcome in patients who do awaken.

Therapeutic hypothermia to a goal temperature of 33 °C is frequently used as a neuroprotective strategy following cardiac arrest, despite two large clinical trials, TTM and TTM-2, demonstrating equivalent outcomes with the use of therapeutic normothermia (36–37.5 °C) [43, 44]. The use of therapeutic hypothermia most likely delays awakening [5, 31, 33, 36], possibly through prolongation of the impact of sedation, although this is not a universal finding [37]. It is therefore reasonable to allow a longer period (72 h from rewarming rather than from ROSC) following the use of therapeutic hypothermia to maximize the number of patients in whom neuroprognostication is rendered unnecessary.

Although neuroprognostication is relevant only to patients who remain comatose (do not awaken) following cardiac arrest, awakening in the ICU must not be equated with neurological prognosis: the persistence of coma beyond 72 h or even seven days does not automatically imply a poor long-term outcome. A substantial number of comatose survivors will awaken beyond this period; 10–22% of all cardiac arrest survivors in the ICU not treated with hypothermia will awaken > 72 h from ROSC, and 10–19% of patients treated with hypothermia will awaken > 72 h from rewarming [5, 27–29, 35, 39]. Of patients who awaken beyond this period, 67–88% will have a good long-term functional outcome [20, 31, 32, 39]. When the waiting period in the ICU is extended to 7 days, 83–100% of patients who eventually awaken will have done so within this time period [33, 35, 38]. In a study from Taiwan, where WLST is restricted, 7% of all cardiac arrest survivors woke beyond 7 days from ROSC, of whom 74% had an excellent functional outcome (CPC = 1) at 6 months [38]. Multiple instances of awakening beyond 2 weeks [31, 32, 36, 38] or even 4 weeks [13, 14, 36, 39] have been described. In the TTM clinical trial, seven patients awoke between days 15 and 22, of whom three (43%) had a good long-term functional outcome [31]. Neuroprognostication in patients with persistent coma beyond 72 h from ROSC/rewarming must therefore be based on objective predictors with low FPRs, as described below, rather than the persistence of coma alone.

Of note, awakening from coma following cardiac arrest is variably defined in the literature as follows: a Glasgow Coma Scale (GCS) score > 8 [33, 34, 39]; a Richmond Agitation Sedation Score >  − 2 [28, 29]; orientation to either person, situation, or place [20, 38]; and (most commonly) the ability to follow commands as documented by a GCS motor score of 6 [4, 5, 27, 31–33, 35–39]. “Early” awakening has also been variably defined: within 72 h from ROSC [5, 27, 33, 34, 37], 48 h from rewarming [20, 35], 72 h from rewarming [4, 20, 31, 39], 48 h from cessation of sedation [28, 29, 32], 5 days from ROSC [36], 7 days from ROSC [33, 38], and 7 days from rewarming [35]. Regardless of definition, patients who awaken early are more likely to have a good functional outcome at 3–6 months than patients who awaken late: 82–97% vs. 67–93% [20, 31, 32, 38, 39].

### Good Practice Statement 2

We recommend that assessment of the neurological prognosis of comatose survivors of cardiac arrest be performed in the absence of sedation or other potential confounders (strong recommendation, evidence cannot be graded).

### Rationale

Sedation will delay awakening following cardiac arrest, and waiting an appropriate length of time for sedation to clear may permit awakening and render neuroprognostication unnecessary [28, 32]. Sedation may also confound EEG, and the use of opioids may result in pupillary constriction, confounding the subjective evaluation of pupillary reactivity [45]. The duration of sedative effect is dependent on medication half-life, duration of infusion, hepatic and renal function, drug interactions, patient age, temperature, and comorbidities, among others. The involvement of a clinical pharmacist may help clarify the expected duration of sedative effect in complex critically ill patients. Other factors that may confound the neurological examination and delay awakening following cardiac arrest include, but are not limited to, seizures [27, 31, 36], hypothermia [5, 31, 33, 36], sepsis, renal failure [29], delirium [32], and hepatic encephalopathy. A defined time period of waiting cannot be recommended to cover all potential confounders. Instead, this time period must be individualized with careful consideration of all relevant factors. Some predictors, such as SSEP and quantitative pupillometry, may be less subject to confounding.

### Good Practice Statement 3

We recommend that factors that impact overall prognosis, such as a poor baseline level of functioning, preexisting illness associated with limited life-expectancy, and multiorgan failure, be considered prior to, and distinct from, assessment of the neurological prognosis of comatose survivors of cardiac arrest (strong recommendation, evidence cannot be graded).

### Rationale

Neuroprognostication should not be conflated with an overall assessment of prognosis. Given the definition of a poor outcome in our systematic review, patients with severe dependence and disability at baseline are outside the scope of these guidelines. Similarly, assessment of long-term neurological outcome may not be relevant in critically ill patients with a high short-term risk of death from multiorgan failure. Assessment of long-term neurological prognosis may also not be relevant in patients with a poor prognosis for long-term survival from conditions such as advanced malignancy.

### Good Practice Statement 4

We recommend that assessment of the neurological prognosis of comatose survivors of cardiac arrest be multimodal, with consideration of the complete clinical scenario, and never based on a single variable (strong recommendation, evidence cannot be graded).

### Rationale

As previously described, an element of uncertainty is inherent in neuroprognostication, even with predictors considered “reliable.” Therefore, it is critical that a thorough assessment be performed with every patient and that the entirety of the clinical picture be considered. This uncertainty is related in part to the inability of studies to fully account for the self-fulfilling prophecy while evaluating the reliability of a predictor [46]. In addition, the possibility of technical, operator, or interpretation error while evaluating a predictor is always present [45, 47–49], and the ability to account for confounders, such as sedation, is imperfect. The use of multiple modalities of assessment, such as clinical examination, imaging, and electrophysiological studies, will mitigate the risk of error from a single modality. Information from these modalities should be largely consistent. For example, the reliability of a subjective determination of nonreactive pupils should be called into question and operator error should be considered in a comatose cardiac arrest survivor with a withdrawal motor response, reactive EEG, and normal brain imaging at 72 h.

### Good Practice Statement 5

We recommend that in the absence of reliable (or multiple moderately reliable) predictors of outcome, surrogates of cardiac arrest survivors who remain comatose at the time of neuroprognostication should be counseled that the likelihood, extent, and time course of neurological recovery is uncertain. Surrogates should also be counseled that the timeline of any functional recovery that does occur may extend from several days to several months (strong recommendation, evidence cannot be graded).

### Rationale

Clinicians should directly acknowledge uncertainty when the prognosis is indeterminate. Many patients will suffer a poor outcome or recover only limited function. When functional recovery does occur the time course of recovery is highly variable. Patients may awaken beyond 72 h and achieve a good functional outcome prior to hospital discharge [5, 27, 29, 33, 35, 36]. Awakening and progressive improvement may also occur following discharge, especially in the first 3–6 months [18–20].

### Good Practice Statement 6

We recommend an extended period of observation for signs of neurological recovery in comatose survivors of cardiac arrest with an indeterminate prognosis, if consistent with the goals of care as established through discussions with patient surrogates (strong recommendation, evidence cannot be graded).

### Rationale

The appropriate length of observation must be established through discussion with surrogates based on a best estimate of the patient’s willingness to undergo an extended duration of life-sustaining treatment. Although the majority of patients who have a good long-term functional recovery will have awakened in the first 2 weeks, a longer period of supportive care and observation, extending over three or more months, will provide a higher level of certainty in the final outcome [20, 31, 32, 38, 39]. The possibility of a good functional recovery in the setting of a chronic disorder of consciousness is never zero but does decline significantly beyond this period following cardiac arrest [14]. Although responsiveness to the outside environment may return in patients considered to be in a vegetative/unresponsive-wakeful state, this is infrequently associated with functional recovery [14]. Several other factors may impact the duration of observation. Supportive care over several months often requires invasive measures, such as tracheostomy and percutaneous gastrostomy, which may not be acceptable to all. An extended duration of supportive care may impose a financial and caregiver burden on surrogates, particularly in low- and middle-income countries, and large out-of-pocket health care expenditures may be associated with poverty in these settings [23, 24, 50]. A transition to hospice care in the setting of a persistent disorder of consciousness several months following cardiac arrest may involve withdrawal of artificial nutrition. This is medically, legally, and ethically feasible in many, but not all, states and countries [51–54]. Surrogates should be counseled on local laws regarding withdrawal of artificial nutrition or other forms of life support.

### Recommendations: Clinical Variables as Predictors

#### Outcome: Functional Outcome

##### Question 1

When counseling surrogates of comatose adult survivors of cardiac arrest, should age be considered a reliable predictor of functional outcome assessed at 3 months or later?

##### Description of the predictor

Older age may be a surrogate for baseline infirmity, comorbidities, and diminished cerebral reserve. Most studies incorporate age as a continuous variable, although some dichotomize at various cutoffs.

##### Recommendation

When counseling surrogates of comatose survivors of cardiac arrest, we suggest the patient’s age alone not be considered a reliable predictor of poor functional outcome assessed at 3 months or later (weak recommendation; low-quality evidence).

##### Rationale

The body of evidence was downgraded for risk of bias in the domains of study participation, study confounding, and the self-fulfilling prophecy. Most studies were associated with a high risk of bias from the self-fulfilling prophecy. Some studies conducted in East Asian countries where withdrawal of life support is restricted were assessed to have a low risk of bias in this domain [55–59]. Of note, in studies conducted in these countries, age achieved statistical significance as an independent predictor of long-term functional outcome in some, but not all, studies [55–59]. Effect size was typically moderate when age was an independent predictor, suggesting that a substantial number of older patients may achieve a good functional outcome when other favorable factors are present. Although the body of evidence overall was thought to indicate an association between older age and poor outcomes, age could not be considered moderately reliable because of inconsistency in the body of evidence not fully explained by study characteristics, uncertain effect size, and the absence of a clear age threshold at which a poor outcome is inevitable. The optimal use of age as a predictor may therefore be as a component of a validated prediction model.

#### Question 2

When counseling surrogates of comatose adult survivors of cardiac arrest, should the initial cardiac rhythm be considered a reliable predictor of functional outcome assessed at 3 months or later?

##### Description of the Predictor

Shockable rhythms, ventricular fibrillation or pulseless ventricular tachycardia, require immediate defibrillation [60]. These rhythms may occur earlier in the time period after cardiac arrest and are often caused by ischemic heart disease. Among nonshockable rhythms, asystole is often a terminal rhythm, whereas PEA encompasses a wide variety of electrical activity and may be associated with a reversible etiology [60].

##### Recommendation

When counseling family members and/or surrogates of comatose survivors of cardiac arrest, we suggest the initial cardiac rhythm alone not be considered a reliable predictor of poor functional outcome assessed at 3 months or later (weak recommendation; low quality evidence).

##### Rationale

The body of evidence was downgraded for risk of bias in the domains of study participation and the self-fulfilling prophecy. Unlike with age as a variable, the evidence was consistent; almost all included studies demonstrated an independent association between a nonshockable rhythm and poor outcome. The evidence was downgraded for imprecision because of relatively wide CIs in several studies. This variable could not be considered moderately reliable because of the large FPR (13–40%) [61–63]. The presence of other favorable factors, such as a rapid ROSC, may mitigate the impact of a nonshockable rhythm. Therefore, similar to age, this variable is best considered in the context of a validated prediction model.

#### Question 3

When counseling surrogates of comatose adult survivors of cardiac arrest, should the time to ROSC be considered a reliable predictor of functional outcome assessed at 3 months or later?

##### Description of the Predictor

Since cardiopulmonary resuscitation (CPR) cannot typically achieve cerebral perfusion equivalent to spontaneous circulation, it is logical to assume that a longer time to ROSC will predict greater neurological injury and worse outcomes [60]. Time to ROSC has been variably defined but typically includes a no-flow period between the onset of cardiac arrest and initiation of CPR and a low-flow period between the start of CPR and ROSC.

##### Recommendation

When counseling family members and/or surrogates of comatose survivors of cardiac arrest, we suggest the time to ROSC alone not be considered a reliable predictor of poor functional outcome assessed at 3 months or later (weak recommendation; moderate quality evidence).

##### Rationale

The body of evidence was downgraded for risk of bias in the domains of study participation, study confounding, and the self-fulfilling prophecy. The evidence was both consistent and relatively precise in demonstrating an independent association between time to ROSC and poor outcome. This variable could not be considered moderately reliable in isolation because of the large FPR (up to 32% for a time to ROSC > 25 min in one study) [63]. Although it is possible that a threshold time to ROSC at which the probability of good long-term functional outcome is infinitesimal exists, such a threshold has not been clearly identified in the literature. Other concerns include the difficulty of accurate measurement, especially of the no-flow period [64]. The quality of CPR is critically important [60] but also difficult to consistently measure. As with age and initial rhythm, this variable may be best considered as a component of a validated prediction model.

#### Question 4

- When counseling surrogates of comatose adult survivors of cardiac arrest, should the bilateral absence of a pupillary light response, assessed at least 72 h from ROSC, be considered a reliable predictor of functional outcome assessed at 3 months or later?
- When counseling surrogates of comatose adult survivors of cardiac arrest, should the bilateral absence of a corneal reflex, assessed at least 72 h from ROSC, be considered a reliable predictor of functional outcome assessed at 3 months or later?

##### Description of the Predictor

Where appropriate, an evaluation for death by neurological criteria should be performed on the basis of published guidelines and institutional protocols [65]. In patients who do not meet such criteria, the pupillary light response and the corneal reflex have long been used in neuroprognostication. The Levy neuroprognostication criteria for nontraumatic coma [66] and the 2006 American Academy of Neurology (AAN) practice parameter [67] both identified the absence of these reflexes as highly predictive of a poor outcome. Although the pupillary response has been assessed as early as a few hours from ROSC in some studies, both these reflexes are more commonly assessed 72 h from ROSC to minimize confounding. Quantitative pupillometry provides precise measurements of both size and reactivity, which can be quantified with proprietary measures such as the neuropupillary index [68, 69].

##### Recommendation

- When counseling family members and/or surrogates of comatose survivors of cardiac arrest, we suggest the bilateral absence of a pupillary light response, assessed at least 72 h from ROSC, be considered a reliable predictor of poor functional outcome assessed at 3 months or later. This recommendation is conditional on accurate assessment without confounding by medication, hypothermia, or prior surgery and an overall clinical picture consistent with severe, widespread neurological injury (weak recommendation; moderate quality evidence).
- When counseling family members and/or surrogates of comatose survivors of cardiac arrest, we suggest the bilateral absence of the corneal reflex alone, assessed at least 72 h from ROSC, not be considered a reliable predictor of poor functional outcome assessed at 3 months or later (weak recommendation; moderate quality evidence).

##### Rationale

The evidence was downgraded for risk of bias primarily from the self-fulfilling prophecy, although some studies demonstrated potential bias from study participation, study confounding, and prognostic factor measurement. The evidence was found to be consistent and precise, with an FPR ≤ 3% for the bilateral absence of the pupillary light response and an upper limit of the 95% CI < 10%, in most (but not all) studies. The sensitivity of this predictor is relatively low (24–50%) in most, but not all, studies. The pupillary light response is less susceptible to confounding and may be assessed 72 h from ROSC, conditional on the absence of hypothermia or other potential confounders at the time of assessment. The most common potential confounders include medications such as mydriatic ophthalmic drops and nebulized bronchodilators [70] and prior ophthalmic surgery. Sedatives and neuromuscular blockade used at moderate therapeutic doses do not typically abolish this response [71–74]. Although the FPR of the pupillary light response is low (but not zero), multiple studies have demonstrated measurement error with this reflex as well [45, 48, 49]. Up to one third of pupils judged to be nonreactive by manual assessment in one study were reactive when assessed with quantitative pupillometry [49]. The probability of error with manual determination of a nonreactive pupil may be highest with small pupils [45]. When a pupillometer is available, quantitative pupillometry should therefore be used to confirm the bilateral absence of a pupillary light response, given the consequences of a false positive prediction. Although other numerical thresholds corresponding to “sluggish” pupils have been evaluated following cardiac arrest in smaller studies of quantitative pupillometry, this body of evidence is insufficient to support a recommendation [69, 75, 76]. When pupillometry is unavailable, the bilateral absence of the pupillary light response must be confirmed by an experienced clinician. The use of a magnifying lens or ophthalmology consultation when bilateral absence of the pupillary light response is suspected may also minimize false positives.

The body of evidence for the corneal reflex was downgraded for risk of bias in the QUIPS domains of study participation, prognostic factor measurement, study confounding, and the self-fulfilling prophecy. The corneal reflex could not be considered reliable because of an FPR higher than our criteria for reliable and moderately reliable predictors. For example, in one prospective multicenter registry-based study in a setting with lower risk of bias from the self-fulfilling prophecy, the FPR was 16% [77]. The higher FPR for the corneal reflex may reflect confounding from factors such as residual neuromuscular blockade and sedation, as well as measurement error [4, 78]. It is possible that the FPR of this predictor may be low when the reflex is tested appropriately. In one survey, many clinicians self-reported application of stimulus predominantly to the bulbar temporal conjunctiva (whereas noxious perception is maximal in the central region or limbus) and used stimuli that might have been insufficient to provoke the reflex [79].

#### Question 5

When counseling surrogates of comatose adult survivors of cardiac arrest, should an absent or extensor best motor response, assessed at least 72 h from ROSC (or 72 h from rewarming in patients treated with therapeutic hypothermia) be considered a reliable predictor of functional outcome assessed at 3 months or later?

##### Description of the Predictor

The best motor response to stimulation is a component of the GCS. The ability to follow commands (M6) is a sign of awakening. An absent (M1) or extensor (M2) best motor response in comatose survivors of cardiac arrest may predict a poor outcome. The motor response was part of the Levy criteria for prognostication in nontraumatic coma as well as the 2006 AAN practice parameter [66, 67]. This predictor is typically assessed a minimum of 72 h after ROSC/rewarming to minimize confounding by sedation, residual neuromuscular blockade, and hypothermia.

##### Recommendation

When counseling family members and/or surrogates of comatose survivors of cardiac arrest, we suggest that an absent or extensor best motor response alone, assessed at least 72 h from ROSC (or 72 h from rewarming in patients treated with therapeutic hypothermia) not be considered a reliable predictor of poor functional outcome assessed at 3 months or later (weak recommendation; low quality evidence).

##### Rationale

The body of evidence was downgraded for risk of bias, primarily in the domains of study participation, study confounding, and the self-fulfilling prophecy. Inconsistency was present in the body of evidence. Studies prior to 2006 mostly reported zero false positives. However, more recent studies indicate much lower predictive accuracy, with a 15–30% FPR [4, 61–63, 80, 81]. Despite the high FPR in more recent studies, overall, this finding was an independent predictor of poor outcome. Imprecision was present, with wide CIs in several studies. This finding could not be considered a moderately reliable predictor because of inconsistency in the evidence not entirely explained by study characteristics and a high FPR in more recent studies. An additional concern was potential confounding by sedation, residual neuromuscular blockade, critical illness neuromyopathy, chronic severe polyneuropathy, encephalopathy, and variable intensity of applied stimulus.

Conversely, a motor response of withdrawal or localization may predict good long-term functional outcome with moderate accuracy [10, 82, 83]. In a post hoc analysis of data from the TTM trial, which included structured neuroprognostication and limitations on WLST, a GCS-M ≥ 4 on day 4 predicted good long-term functional outcome with approximately 93% sensitivity and 77% specificity [83]. In another study, a GCS-M score of 4 or 5 at the time of hospital admission following OHCA had a sensitivity of 12% but a specificity of 98% for good long-term functional outcome [82]. However, A best motor response of flexion alone (GCS-M = 3) is relatively nonspecific and may be seen even after death by neurological criteria as a spinal reflex.

#### Question 6

When counseling surrogates of comatose adult survivors of cardiac arrest, should the occurrence of myoclonus < 48 h from ROSC, in the absence of concomitant EEG evaluation, be considered a reliable predictor of functional outcome assessed at 3 months or later?

##### Description of the Predictor

This predictor refers to spontaneous myoclonus, the involuntary spasmodic contraction of groups of axial or appendicular muscles, in the early period (< 48 h) following ROSC from hypoxia-induced neuronal hyperactivity. Although EEG may be performed to establish the source of myoclonus (cortical, subcortical, or other), detect seizures, and evaluate background, this predictor refers to the purely clinical finding without concomitant EEG evaluation. Status myoclonus, a severe form of early myoclonus, has been defined as spontaneous, repetitive, unrelenting, generalized multifocal myoclonus involving the face, limbs, and axial musculature in comatose patients < 48 h from ROSC [67]. Some definitions of this phenomenon have specified a duration of 30 min [84, 85]. Status myoclonus, in particular, has traditionally been associated with a poor prognosis. Of note, Lance–Adams syndrome, characterized by the delayed onset and subsequent persistence of sporadic myoclonus in survivors of cardiac arrest, is a distinct entity frequently associated with good functional recovery [86].

##### Recommendation

When counseling family members and/or surrogates of comatose survivors of cardiac arrest, we suggest that the occurrence of myoclonus < 48 h from ROSC, in the absence of concomitant EEG evaluation, not be considered a reliable predictor of poor functional outcome assessed at 3 months or later (weak recommendation; very low quality evidence).

##### Rationale

Few studies met all of the eligibility criteria to support a recommendation; most were ineligible because of premature evaluation of poor outcome (at discharge), small sample size (*N* < 100), or both. The limited eligible evidence was downgraded for risk of bias in the domains of prognostic factor measurement and the self-fulfilling prophecy. There is variation in the definitions and nomenclature related to early postanoxic myoclonus in the literature. A meaningful FPR could not be estimated given the limitations in the body of evidence. Our decision to recommend not using this predictor is based on two factors. First, multiple studies since 2006 have described good outcomes despite the occurrence of early postanoxic myoclonus [62, 85, 87–93]. It is possible that status myoclonus, a subtype of the predictor in this question, may be an accurate predictor of poor outcome when evaluated exactly as defined (generalized, unremitting, multifocal). However, there was insufficient high-quality evidence meeting our criteria to support this hypothesis. Second, it is not clear that clinicians from the variety of disciplines likely to witness early myoclonus can consistently and reliably distinguish “true” generalized status myoclonus from other forms of postanoxic myoclonus. In one study, the interrater reliability of neurologists for the assessment of features of postanoxic myoclonus, such as generalization, stimulus-sensitivity, and localization (proximal vs. distal), was poor [94]. A recommendation to support the use of status myoclonus in clinical practice requires larger studies with explicit criteria for recognition of the prognostic variable and assessment of outcome at an appropriate time point.

Although this predictor refers to a purely clinical finding, concomitant EEG is often performed to identify the source of myoclonus (cortical, subcortical, or other). However, there is insufficient evidence to support the hypothesis that origin of myoclonus reliably predicts long-term outcome. In one study, a comparable number of patients with cortical and subcortical origin of myoclonus on EEG achieved a good outcome at discharge (12% vs. 16%), although a higher number of patients with cortical myoclonus were comatose at the time of discharge (82% vs. 39%) [95]. Several studies have attempted to identify EEG patterns associated with clinical posthypoxic clinical myoclonus that are predictive of poor outcome. In one study, the presence of a burst suppression background with high-amplitude polyspikes time locked with myoclonic jerks was invariably associated with poor outcome at discharge, whereas half of patients with a continuous background and narrow vertex spike-wave discharges time locked to myoclonus had a good discharge outcome [96]. There is insufficient high-quality evidence to establish the added prognostic value of clinical myoclonus beyond the “malignant” EEG patterns alone. For example, in one study, the presence of posthypoxic myoclonus was not associated with poor outcome in a population of post-cardiac-arrest patients with periodic discharges on EEG [97].

#### Question 7

When counseling surrogates of comatose adult survivors of cardiac arrest, should a diffuse pattern (across vascular distributions in the bilateral anterior and posterior circulation, with involvement of cerebral cortex and deep gray matter) of loss of gray–white differentiation with sulcal effacement on noncontrast CT imaging of the brain performed at least 48 h from ROSC be considered a reliable predictor of functional outcome assessed at 3 months or later?

##### Description of the Predictor

Hypoxic-ischemic injury to brain parenchyma may result in cytotoxic edema, with loss of differentiation between gray and white matter on noncontrast CT of the brain. Infarction as a result of hypoxic-ischemic injury will eventually result in frank hypodensity across gray and white matter, with subacute and chronic ischemic infarction demonstrating a radiodensity of approximately < 20 Hounsfield units [98]. The presence of unequivocal diffuse infarction encompassing the majority of gray and white matter with widespread sulcal and basal cisternal effacement confirms devastating brain injury and a poor prognosis. This finding lacks clinical equipoise for systematic evaluation. The predictor in this PICOTS question does not refer to unequivocal diffuse infarction and instead refers to diffuse homogenization of gray and white matter radiodensity accompanied by diffuse sulcal effacement, a sign of diffuse cytotoxic edema that is observed prior to unequivocal radiographic evidence of cerebral infarction. Although most often identified through subjective assessment, several studies have described quantification of the average gray–white radiodensity ratio (avGWR). As originally described, measurements of gray and white matter radiodensity are performed bilaterally at three levels using a 10-mm^2^ elliptical measuring cursor and 5-mm slice thickness: (1) basal ganglia level, with the putamen, caudate nucleus, internal capsule, third ventricle, and sylvian fissure visible; (2) centrum semiovale level, defined as the axial slice 5 mm above the lateral ventricles; and (3) high convexity level, defined as the axial slice 5 mm above the centrum semiovale level [99]. At the basal ganglia level, gray matter radiodensity may be measured within the putamen or caudate nucleus and white matter radiodensity may be measured within the internal capsule. At the centrum semiovale and high convexity levels, radiodensity is measured within the medial cortex (gray matter) to avoid beam hardening from bone and within adjacent white matter. The ratio of gray to white matter radiodensity is then calculated at each level and averaged across both sides. Studies have examined the predictive value of CT performed at various time points, from the immediate post-ROSC period to 72 h following cardiac arrest.

##### Recommendation

When counseling family members and/or surrogates of comatose survivors of cardiac arrest, we suggest that a diffuse pattern (across vascular distributions in the bilateral anterior and posterior circulation, with involvement of cerebral cortex and deep gray matter) of loss of gray–white differentiation with sulcal effacement on noncontrast CT imaging of the brain performed at least 48 h from ROSC be considered a moderately reliable predictor of poor functional outcome assessed at 3 months or later (weak recommendation; very low quality evidence).

##### Rationale

Most studies reviewed during the systematic review were ineligible because of premature evaluation of outcome (at discharge), small sample size, or both. The body of eligible evidence was downgraded for risk of bias in the domains of prognostic factor measurement, study participation, and the self-fulfilling prophecy. Limited inconsistency was present but could be entirely explained by differences in timing of prognostic factor measurement. CT scans performed within 2–12 h of ROSC (too early for ischemic changes to be consistently visible) failed to predict outcome in some studies [56, 100]. The evidence was downgraded for indirectness because the highest quality studies used the measured avGWR rather than the technique most clinicians will use—subjective determination of the loss of gray–white differentiation. Imprecision was present because of small sample size in several studies. The overall specificity of an avGWR < 1.1–1.8 measured from scans performed beyond 12–24 h was high, at 95–100% [81, 101, 102]. Given the high specificity, with downgrades for imprecision and risk of bias, this predictor was considered moderately reliable—to be used during prognostication only when at least one other moderately reliable or reliable predictor is present. Because CT and MRI both assess structural hypoxemic-ischemic injury, these moderately reliable predictors should ideally be combined with a neurophysiological study, such as EEG or SSEP, during prognostication. There are several important caveats to the use of this predictor in clinical practice. First, most clinicians will subjectively assess the predictor because quantification of avGWR requires specialized expertise and software that is inaccessible to most clinicians at the bedside. Quantification of avGWR using validated parameters and protocols should be performed where available [81, 99]. Assessment of gray–white differentiation and sulcal effacement should not be attempted in the presence of artifacts caused by motion, beam hardening from bone, and metal artifact from EEG leads [103]. To minimize the risk of confounding from artifact or misdiagnosis of an acute ischemic stroke, homogenization of radiodensity must be present across vascular distributions in the bilateral anterior and posterior circulations and must include deep gray matter structures, such as the caudate nucleus and putamen. We suggest conservative timing of CT, at least 48 h from ROSC, to allow a greater time interval for hypoxic-ischemic changes to develop. A noncontrast CT is often performed shortly after ROSC to identify a possible neurological cause of cardiac arrest. This “etiology” scan is typically performed too early for ischemic changes to develop [56, 100] and should not be used for prognostication.

#### Question 8

When counseling surrogates of comatose adult survivors of cardiac arrest, should a diffuse pattern (across vascular distributions in the bilateral anterior and posterior circulation, with involvement of cerebral cortex and deep gray matter) of restricted diffusion on MRI of the brain performed between 2 and 7 days from ROSC be considered a reliable predictor of functional outcome assessed at 3 months or later?

##### Description of the Predictor

Hypoxic-ischemic injury to brain parenchyma results in restriction of the diffusion of water molecules. In MRI diffusion weighted imaging (DWI) sequences, the intensity of each image voxel reflects the rate of water diffusion and therefore the severity of cellular injury. This predictor refers to the presence widespread diffusion restriction in the bilateral cortex and deep gray matter across vascular distributions, indicative of global injury. Although most often identified through subjective assessment [101, 104], diffusion restriction may be quantified with an apparent diffusion coefficient (ADC) value in each image voxel. Some studies have attempted to identify a threshold percentage of image voxels below a critical ADC cutoff that predicts poor neurological outcome [105–107]. Other methods of quantification have also been studied [101, 108–110]. Although changes in ADC likely develop within minutes of hypoxic-ischemic injury, waiting 48–72 h from ROSC will allow for completion of therapeutic temperature management and the opportunity to assess stability for transport and study completion.

##### Recommendation

When counseling family members and/or surrogates of comatose survivors of cardiac arrest, we suggest that a diffuse pattern (across vascular distributions in the bilateral anterior and posterior circulation, with involvement of cerebral cortex and deep gray matter) of restricted diffusion on MRI of the brain performed between 2 and 7 days from ROSC be considered a moderately reliable predictor of poor functional outcome assessed at 3 months or later (weak recommendation; very low quality evidence).

##### Rationale

Few studies met eligibility for the systematic review. Most were excluded for inadequate sample size, premature assessment of outcome (at discharge), or both. The body of eligible evidence was downgraded for risk of bias in the domains of study participation, study confounding, and self-fulfilling prophecy. The evidence was downgraded for indirectness because the highest quality studies used quantification of ADC rather than subjective assessment of the MRI. The evidence was also downgraded for imprecision. The FPR was 0–5% in most studies, with a sensitivity of 33–92% depending on the threshold used. The two thresholds validated in higher quality studies for the prediction of long-term outcome are as follows: > 10% of voxels with ADC < 650 × 10^−6^ mm^2^/s [106] and > 2.5% of voxels with ADC < 400 × 10^−6^ mm^2^/s [105]. The most important limitation is that most clinicians will subjectively assess the predictor because quantification of ADC requires specialized expertise and software that is inaccessible to most clinicians at the bedside. To minimize the risk of misdiagnosis, restricted diffusion must be present bilaterally in the anterior and posterior circulation, must be present across vascular distributions, and must involve both cortex and deep gray matter. In addition to hypoxic-ischemic injury, a variety of other conditions can result in restricted diffusion [111], including some in a diffuse pattern, such as hyperammonemic encephalopathy [112]. Seizures and status epilepticus, which are common in comatose survivors of cardiac arrest, may, in particular, result in DWI abnormalities [113, 114]. MRI-DWI should therefore not be used for the purposes of neuroprognostication when seizures (clinical or electrographic) or other possible etiologies of restricted diffusion are present. Obtaining an MRI scan in critically ill patients may be challenging and occasionally risky. Limited monitoring options are available within the MRI scanner for patients with cardiopulmonary instability. In addition, an MRI study requires more time for completion than CT. Because CT and MRI both assess structural hypoxemic-ischemic injury, these moderately reliable predictors should ideally be combined with a neurophysiological study, such as EEG or SSEP, during prognostication.

MRI may demonstrate at least moderate accuracy for the prediction of good long-term outcome [10, 104, 115, 116]. In one study that met our systematic review criteria, the presence of no DWI lesions or an isolated lesion in the cortex or gray matter achieved a sensitivity of 94% and a specificity of 92% for good 6-month functional outcome [104]. The absence of DWI lesions on MRI performed > 72 h from ROSC also achieved a sensitivity of 92–100% and a specificity of 93% for the prediction of good long-term functional outcome in two other studies with sample sizes < 100 [115, 116].

#### Question 9

When counseling surrogates of comatose adult survivors of cardiac arrest, should a suppressed or burst suppression background, with or without periodic discharges, on EEG performed at least 72 h from ROSC (or 72 h from rewarming in patients treated with therapeutic hypothermia) in the absence of sedation or other potential confounders, such as hypothermia, be considered a reliable predictor of functional outcome assessed at 3 months or later?

##### Description of the Predictor

EEG is sensitive to cerebral ischemia, demonstrating suppression of electrical activity at cerebral blood flow < 10 mL/100 g/min [117]. Next to the physical neurological examination, EEG is the oldest tool used for neuroprognostication following cardiac arrest. A variety of EEG patterns and grading systems have been studied as predictors of poor outcome following cardiac arrest. The Synek classification, first described in 1988, has five grades, with grades 4 (suppression, burst suppression, epileptiform discharges plus burst suppression with or without clinical myoclonus, diffuse alpha, and diffuse theta) and 5 (isoelectric) considered “malignant” and indicative of a poor prognosis [63, 80, 118]. More recently, the TTM clinical trial identified the following “highly malignant” EEG patterns: suppression with or without continuous periodic discharges and burst suppression with or without discharges [119]. The American Clinical Neurophysiology Society defines suppression as a background voltage < 10 µV for > 99% of the record and burst suppression as a suppressed (< 10 µV) pattern present for 50–99% of the record [120]. Other EEG patterns that may predict poor outcome include the absence of reactivity to applied stimuli and the presence of electrographic status epilepticus. Although EEG has been evaluated as a predictor as early as the day of cardiac arrest, waiting 72 h from ROSC (or rewarming in patients treated with hypothermia) will minimize the risk of confounding by these factors. Several centers perform continuous EEG monitoring starting soon after ROSC to identify and manage seizures and to assist with neuroprognostication. However, continuous EEG is not yet widely available and routine EEG, typically of < 1 h duration, may instead be performed.

##### Recommendation

When counseling family members and/or surrogates of comatose survivors of cardiac arrest, we suggest that a suppressed or burst suppression background, with or without periodic discharges, on EEG performed at least 72 h from ROSC (or 72 h from rewarming in patients treated with therapeutic hypothermia) in the absence of sedation or other potential confounders, such as hypothermia, be considered a moderately reliable predictor of poor functional outcome assessed at 3 months or later (weak recommendation; low quality evidence).

##### Rationale

The body of evidence was downgraded for risk of bias in the domains of study participation, prognostic factor measurement, study confounding, and the self-fulfilling prophecy. The evidence was consistent in demonstrating that the EEG patterns specified in the PICOTS question predicted poor long-term outcome. Imprecision was present, with wide CIs in several studies. Across studies, in addition to being the most consistent patterns predicting poor outcome, suppression and burst suppression demonstrated a low (< 5%) FPR. These patterns can be detected with routine EEG. In the TTM trial, these patterns had a sensitivity of 50% for poor long-term outcome [119]. They are commonly seen in the first 12–24 h following cardiac arrest but often evolve into less malignant patterns [121]. Suppression and burst suppression may also be artificially induced by sedation and hypothermia, particularly in the first 72 h. Our recommendation to wait 72 h from ROSC (or rewarming) to identify these patterns will minimize false positives but will likely decrease sensitivity. The presence of identical (compared to varied) bursts during burst suppression has been identified as a specific indicator of poor outcome during hypothermia [122]. However, this finding is somewhat subjective and lacks a sufficient body of evidence to support a recommendation for routine use as a predictor in the presence of potential confounders, such as hypothermia. In addition, it is often a transient finding that relies on continuous EEG, which is not universally available [122, 123].

EEG overall may be confounded by several factors, including sedation, metabolic derangements, and body temperature. The primary concerns with EEG reactivity as a predictor include the subjectivity of this finding, with only moderate interrater agreement [124, 125], and some studies that demonstrate a higher FPR [119, 123, 126, 127], up to 44% in one study [80]. The identification of electrographic status epilepticus can also be subjective, and there is an insufficient body of evidence evaluating the prognostic value of status epilepticus strictly defined using standardized criteria [120, 128]. The EEG background from which status epilepticus evolves, or reverts to following treatment, may be a more important factor in predicting outcome [123, 129, 130]. The presence of discrete electrographic seizures is not a reliable predictor of outcome [123]. Similarly, the EEG background may be of greater importance than the presence of superimposed generalized or lateralized periodic discharges, a frequent finding in the first 48–72 h following ROSC [123]. Generalized alpha and theta patterns are relatively uncommon, and an insufficient body of high-quality evidence exists to support a recommendation to use these patterns in neuroprognostication.

Of note, EEG may identify patients likely to have a good outcome, albeit with only moderate accuracy. In one study, the appearance of a continuous background on EEG within 24 h achieved a sensitivity of 85% and a specificity of 80% for prediction of good 6-month functional outcome [121], whereas in another, the appearance of a continuous background within 12 h achieved a sensitivity of 19% and a specificity of 98% [131]. Similarly, the presence of EEG reactivity 12–24 h from ROSC achieved a sensitivity of 75% and a specificity of 65% for the prediction of good long-term outcome [126].

#### Question 10

When counseling surrogates of comatose adult survivors of cardiac arrest, should the bilateral absence of the N20 wave, with preservation of responses at Erb’s point and the cervical spine, on SSEP testing performed at least 48 h from ROSC be considered a reliable predictor of functional outcome assessed at 3 months or later?

##### Description of the Predictor

SSEPs evaluate the conduction of a sensory signal from the point of stimulation to the cerebral cortex. The short-latency (20 ms) N20 negative cortical peak following stimulation of the median nerve may be used to assess the integrity of thalamocortical connections following cardiac arrest [132]. The bilateral absence of N20 responses is thought to indicate severe injury and a poor prognosis. Previous guidelines have indicated a low FPR for this predictor [60, 67, 78]. N20 responses are not abolished by sedation or neuromuscular blockade [133]. The integrity of conduction below the brain is established with electrodes placed at Erb’s point (EPi: above the clavicle, lateral to the sternomastoid muscle) and the cervical spine (C2s/C5s: C2 or C5 spinous process) [132]. The scalp electrode is placed 2 cm posterior to the C3 or C4 EEG electrode site, over the somatosensory cortex contralateral to the side of stimulus [132].

##### Recommendation

When counseling family members or surrogates of comatose survivors of cardiac arrest, we suggest that the bilateral absence of the N20 wave, with preservation of responses at Erb’s point and the cervical spine, on SSEP testing performed at least 48 h from ROSC be considered a reliable predictor of poor functional outcome assessed at 3 months or later. This recommendation is conditional on accurate measurement and interpretation of the SSEP and an overall clinical picture consistent with severe, widespread neurological injury (weak recommendation; moderate quality evidence).

##### Rationale

The body of evidence was downgraded for risk of bias in the domains of study participation, study confounding, and the self-fulfilling prophecy. The body of evidence was consistent and relatively precise in demonstrating high specificity of the predictor for poor long-term functional outcome. The FPR was overall < 3%, with the upper limit of the 95% CI < 10%. Sensitivity was highly variable, from 10 to 75%. This likely reflected clinician selection of cases to undergo SSEP in most studies rather than routine performance of the study in all participants. Of note, the impact of the self-fulfilling prophecy cannot be easily measured. One study that used statistical modeling based on rates of withdrawal of life support in published studies estimated that a true or “natural” FPR might be as high as 8% [46]. It is important, therefore, to acknowledge an element of uncertainty during counseling, even with this “reliable” predictor. It is also critical that the study be performed and interpreted correctly. Confirmation of the presence of responses at Erb’s point and the cervical spine is a prerequisite because extracranial injury, including at the level of the cervical spine with hanging or other trauma, may abolish the N20 response [134]. Although interrater reliability to determine the absence of N20 responses appears to be high [135, 136], inaccurate interpretation may occur in the context of background electrical noise, which is common in the ICU [47]. A single dose of a neuromuscular blocking agent may be administered to eliminate muscle artifact while performing the study [137]. Studies should be interpreted as indeterminate in the presence of significant background noise, which may obscure the N20 response. Finally, hypothermia appears to prolong SSEP latency [138–140], and severe hypothermia (20–25 °C) will abolish the N20 response [141]. Although some studies suggest the study remains accurate within 24 h of cardiac arrest [142], we suggest waiting at least 48 h from ROSC to minimize the risk of confounding from hypothermia.

A higher amplitude of the N20 response, measured as the voltage difference from the P25 positive wave, may predict good outcome. In a recent systematic review of predictors of good outcome in comatose survivors of cardiac arrest, a largest measured N20 amplitude > 4 μV at 48–72 h from ROSC achieved a specificity of > 80% and a sensitivity of > 40% for prediction of good long-term functional outcome [10], including in three studies that met criteria for our systematic review [104, 131, 143].

#### Question 11

When counseling surrogates of comatose adult survivors of cardiac arrest, should the serum level of NSE, measured ≤ 72 h from ROSC, be considered a reliable predictor of functional outcome assessed at 3 months or later?

##### Description of the Predictor

Enolase is a glycolytic enzyme, whereas NSE is an isoenzyme specific to neurons and peripheral neuroendocrine cells [144]. In the context of hypoxic-ischemic brain injury, the serum level of NSE has been used as a biomarker to quantify neuronal damage. The 2006 AAN practice parameter identified a threshold of > 33 µg/L as a predictor of poor outcome, with an FPR of 0% [67]. NSE has been evaluated at varied time points, most commonly between 24 and 72 h following ROSC, and with variable thresholds.

##### Recommendation

When counseling surrogates of comatose survivors of cardiac arrest, we suggest that the serum level of NSE alone, measured ≤ 72 h from ROSC, not be considered a reliable predictor of poor functional outcome assessed at 3 months or later until a consistent threshold is validated (weak recommendation; moderate quality evidence).

##### Rationale

The body of evidence was downgraded for risk of bias in the domains of study participation, study confounding, and the self-fulfilling prophecy. Inconsistency in the accuracy of this predictor seems directly related to the threshold selected. For a given threshold, CIs were relatively narrow in eligible studies. A clear biological association between increasing NSE levels and poor outcome was seen across studies, with point estimates of the AUC between 0.78 and 0.91 and an OR of 1.04–37.47. This predictor could not be recommended as a reliable or moderately reliable predictor because of the high FPR for various thresholds. Specifically, an FPR as high as 34% was identified for the threshold of > 33 µg/L used in the 2006 AAN practice parameter [63]. An insufficient body of evidence existed to support other NSE criteria, such as an increase after 24 h, which demonstrated an FPR of 32% in one study [63], or at other time points [145]. Higher NSE thresholds demonstrated progressively lower FPRs. In one study, the FPR was 7% for a threshold of > 60 µg/L and 2% for a threshold > 80 µg/L [63]. Although a higher threshold of > 70–80 µg/L may in fact predict poor outcome with a low FPR [63, 104], an insufficient body of evidence exists at this time to support the use of any single threshold across centers. Additionally, the sensitivity of the test decreases as threshold increases. Single-center validation of biomarker thresholds is challenging given the need for an adequate sample size and appropriate length of follow-up. This recommendation is expected to change if larger multicenter studies identify a threshold using standardized times and techniques of measurement. Because an isoform of NSE is present in circulating erythrocytes and platelets, hemolysis (visible or invisible) in the blood sample should be excluded [146]. The presence of a normal serum NSE level (< 17–18 μg/L) at 24–72 h following ROSC achieved a specificity of > 80% and a sensitivity of > 40% for prediction of good long-term functional outcome in a recent systematic review [10], which included two studies that met criteria for our systematic review [147, 148]. However, these thresholds for good outcome are also potentially subject to variability between centers. Consideration of resource use also weighed against recommending the routine use of NSE, currently a send-out test at most centers.

## Recommendations: Clinical Prediction Models

### Outcome: Functional Outcome

#### Question 1

When counseling surrogates of comatose adult survivors of cardiac arrest, should the OHCA clinical prediction model be considered a reliable predictor of functional outcome assessed at 3 months or later?

##### Description of the Predictor

The OHCA clinical prediction model is focused on patients who have suffered OHCA rather than IHCA and incorporates four admission variables identified as independent predictors of outcome in the original development study [149]. These variables are the initial rhythm, no-flow interval, low-flow interval, lactate, and serum creatinine. The major advantage of the OHCA model is the ability to predict outcome early on ICU admission. The major disadvantage is the difficulty in estimating no-flow and low-flow time. An OHCA score > 60 has been proposed as a threshold with 100% specificity [150, 151].

##### Recommendation

There is insufficient evidence for a recommendation.

##### Rationale

Only one study met all eligibility criteria, including assessment of outcome at 3 months or beyond [152]. However, this study did not report model calibration and could not support a recommendation. Several other studies assessed outcome at discharge [149–151, 153] and reported no evidence of miscalibration [149, 150]. One study with assessment of outcome at discharge was focused on IHCA [151]. All studies were at risk for bias from the self-fulfilling prophecy, which may have impacted components of the prediction model. Additional external validation studies of adequate size with assessment of outcome at 3 months or beyond may impact this recommendation. At this time, it is reasonable to use the model for purposes of research and risk-adjusted quality analysis.

#### Question 2

When counseling surrogates of comatose adult survivors of cardiac arrest, should the CAHP clinical prediction model be considered a reliable predictor of functional outcome assessed at 3 months or later?

##### Description of the Predictor

The CAHP clinical prediction model is focused on patients who have suffered OHCA rather than IHCA and incorporates seven variables identified as independent predictors of outcome in the original development study. These variables included five variables related to cardiopulmonary resuscitation (no-flow interval, low-flow interval, total dose of adrenaline required during CPR, the arrest setting, and the presence of a shockable rhythm), age, and the admission arterial pH [154]. Similar to OHCA, the major advantage of the CAHP model is the ability to predict outcome shortly after ROSC, whereas the major disadvantage is the difficulty in estimating no-flow and low-flow time [151, 153–155]. A CAHP score > 200 has been proposed as a threshold with 95–100% specificity [151, 154].

##### Recommendation

There is insufficient evidence for a recommendation.

##### Rationale

Only one study met all eligibility criteria, including assessment of outcome at 3 months or beyond [152]. However, this study did not report model calibration and could not support a recommendation. Several other studies assessed outcome at discharge [151, 153–155], and one reported no evidence of miscalibration [154]. One study with assessment of outcome at discharge was focused on IHCA [151]. All studies were at risk for bias from the self-fulfilling prophecy, which may have impacted components of the prediction model. Additional external validation studies of adequate size with assessment of outcome at 3 months or beyond may impact this recommendation. At this time, it is reasonable to use the model for purposes of research and risk-adjusted quality analysis.

#### Question 3

When counseling surrogates of comatose adult survivors of cardiac arrest, should the GOFAR clinical prediction model be considered a reliable predictor of functional outcome assessed at 3 months or later?

##### Description of the Predictor

The GOFAR clinical prediction model is focused on patients who have suffered IHCA rather than OHCA and incorporates 13 variables identified as predictors of outcome in the original development study [156]. This model was constructed using data from the GWTG-Resuscitation IHCA database. The variables are all categorical and mostly binary (yes/no): age (stratified in four groups within a range from 70 to ≥ 85 years), baseline CPC = 1, major trauma, acute stroke, metastatic or hematological cancer, septicemia, hepatic insufficiency, admission from skilled nursing facility, medical noncardiac admission, hypotension or hypoperfusion, renal insufficiency/dialysis, respiratory insufficiency, and pneumonia. Of note, this model was not originally created to inform prognostication after cardiac arrest. Instead, the purpose was to select inpatients who have not suffered cardiac arrest but are at high risk for poor outcome should cardiac arrest occur to permit shared decision-making about a do-not-attempt-resuscitation (DNAR) order [156]. All variables are therefore available prearrest. Based on the continuous GOFAR score, four categories are identified on the basis of the likelihood of neurologically intact survival: very low (< 1%), low (1–3%), average (> 3–15%), or higher than average (> 15%).

##### Recommendation

When counseling family members or surrogates of comatose survivors of IHCA, we suggest the GOFAR clinical prediction model alone not be considered a reliable predictor of poor functional outcome assessed at 3 months or later (weak recommendation; moderate quality evidence).

##### Rationale

All studies evaluated outcome at discharge only and were therefore ineligible to support a recommendation. Our decision to recommend not using this model for prognostication in comatose survivors of cardiac arrest, rather than withholding a recommendation based on insufficient evidence meeting our criteria, is based on two factors. First, this was not the original purpose as envisioned by the developers of the model, which was instead to identify patients in whom a DNAR order may be appropriate prior to cardiac arrest [156]. Second, although model calibration was reported with the original study as well as in one external validation study [156, 157], the latter reported systematic underestimation of neurologically intact survival at discharge [157]. At this time, it is reasonable to use the model for purposes of research and risk-adjusted quality analysis in the setting of IHCA.

### Approach to Neuroprognostication in Comatose Survivors of Cardiac Arrest

**Fig. 2:**
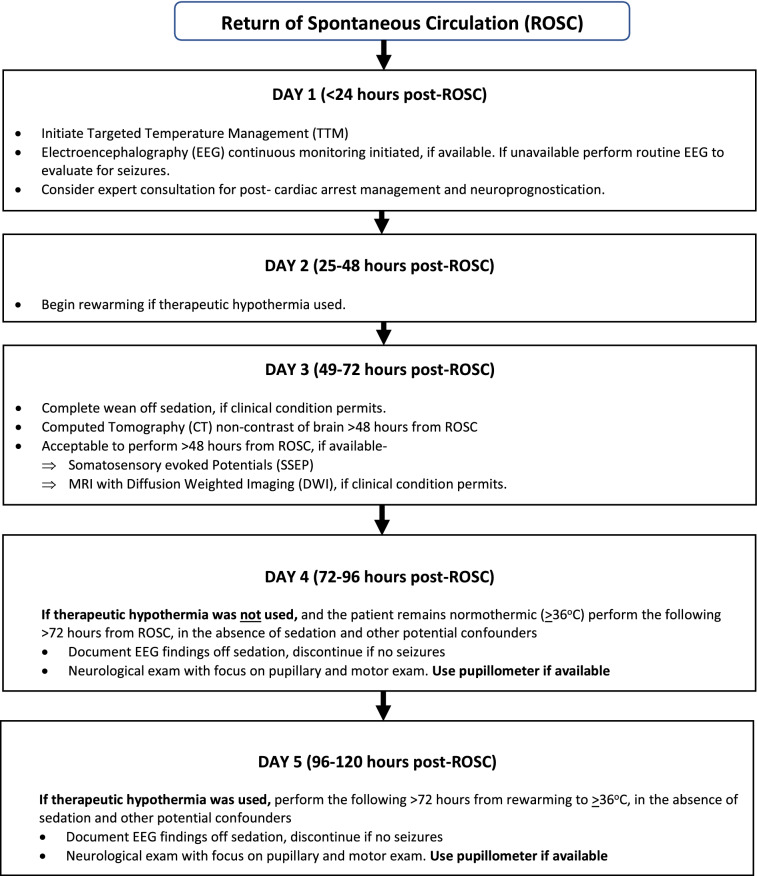
Algorithm for neuroprognostication in adult comatose cardiac arrest survivors: evaluation

**Fig. 3:**
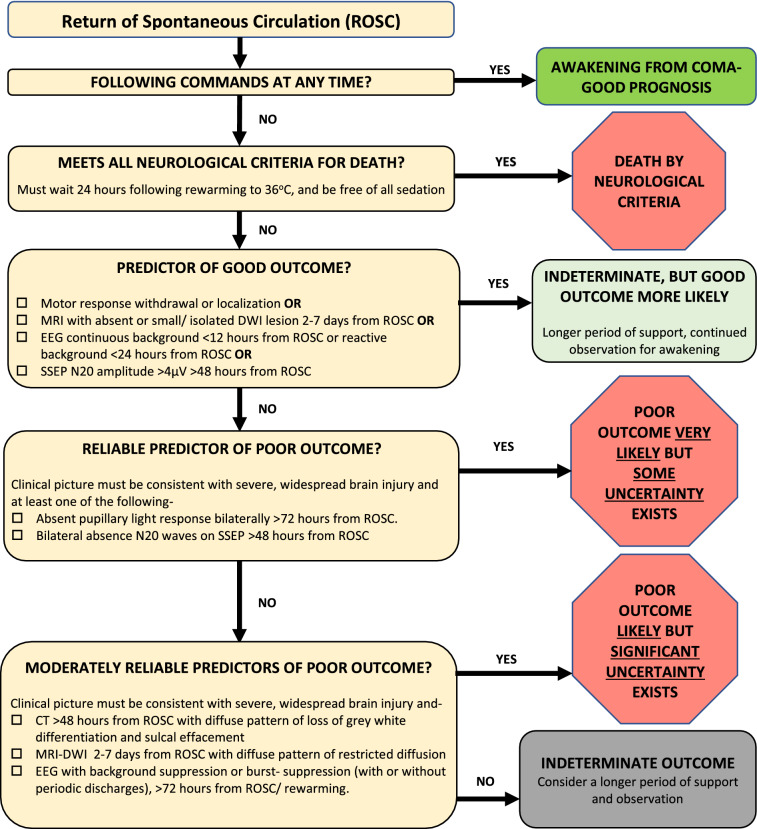
Algorithm for neuroprognostication in adult comatose cardiac arrest survivors: predictors & prognosis

The suggested approach to neuroprognostication in comatose survivors of cardiac arrest, incorporating the recommendations in these guidelines, is in Figs. 2 and 3.

Table 4 lists key considerations with the evidence-based use of predictors in this population.

**Table 4:** Predictors used in neuroprognostication in comatose survivors of cardiac arrest: key considerations

Predictor with time of assessment	Assessment	Interpretation
**Neurological examination**		
Pupillary light response (PLR) ≥ 72 h from Return of spontaneous circulation (ROSC)	Use quantitative pupillometry where available Where a pupillometer is unavailable and the PLR is thought to be absent, consult ophthalmology or use a magnifying glass Consider potential confounders such as medications (mydriatic ophthalmic drops, nebulized bronchodilators) and prior ophthalmic surgery	Bilateral absence of the PLR is a reliable predictor of poor functional outcome at 3 months or later
Neurological exam- motor exam Hypothermia not used- ≥ 72 h following ROSC Hypothermia used- ≥ 72 h following rewarming to normothermia	Motor response to noxious stimulation	A good functional outcome at 3 months or later is more likely than a poor outcome in the presence of withdrawal, localization or command-following at any time An absent or extensor motor response is not reliable for prediction of poor outcome
**Brain imaging**		
Computed tomography (CT) of the brain, noncontrast ≥ 48 h following ROSC	Diffuse pattern- loss of gray–white differentiation and sulcal effacement should be present across vascular distributions in the bilateral anterior and posterior circulation, with involvement of cerebral cortex and deep gray matter (diffuse pattern) Do not use in the presence of artifact from sources such as EEG electrodes, patient movement and beam hardening from bone	A diffuse pattern of loss of gray–white differentiation and sulcal effacement is a moderately reliable predictor of poor functional outcome at 3 months or later
Magnetic resonance imaging (MRI) brain with diffusion weighted imaging (DWI) 2–7 days following ROSC	Diffuse pattern- restricted diffusion should be present across vascular distributions in the bilateral anterior and posterior circulation, with involvement of cerebral cortex and deep gray matter (diffuse pattern) Do not use in the presence of- Artifact from sources such as patient movement Seizures Other potential etiologies of restricted diffusion	A diffuse pattern of loss of restricted diffusion is a moderately reliable predictor of poor functional outcome at 3 months or later A good functional outcome at 3 months or later is more likely than a poor outcome when DWI lesions are absent, or an isolated lesion is present in the cortex or deep gray matter
**Electrodiagnostic**		
Electroencephalography (EEG) Hypothermia not used- ≥ 72 h following ROSC Hypothermia used- ≥ 72 h following rewarming to normothermia	Exclude confounders such as sedation, toxic-metabolic encephalopathy and hypothermia Suppression is defined as a background voltage < 10 µV for > 99% of the record Burst suppression is defined as a suppressed (< 10 µV) pattern present for 50–99% of the record	The presence of suppression or burst suppression on EEG is a moderately reliable predictor of poor functional outcome at 3 months or later
Somatosensory Evoked Potentials (SSEP) ≥ 48 h from ROSC	Responses must be present at Erb’s point and the cervical spine as a prerequisite to prognostication Consider routine use of neuromuscular blockade during testing to minimize artifact Studies should be interpreted as indeterminate in the presence of significant background noise which may obscure the N20 response Severe hypothermia may abolish the N20 response	Bilateral absence of the N20 cortical response is a reliable predictor of poor functional outcome at 3 months or later A good functional outcome at 3 months or later is more likely than a poor outcome when the largest-measured N20 amplitude is > 4 μV at 48–72 h from ROSC

### Future Directions

Only a limited number of predictors had a sufficient body of evidence to support recommendations for use in clinical practice. Although these predictors met criteria for reliability, they were often insensitive. Therefore, a large number of patients will have an indeterminate prognosis on the basis of these guidelines, highlighting the importance of future high-quality neuroprognostication research. The AHA outlined standards for cardiac arrest neuroprognostication research in 2019 [8].

Based on the most common study limitations identified in our systematic review, future studies should consider the following general principles:

1. Outcomes should be assessed at least 3 months, and ideally 6 or more months, following cardiac arrest. Assessors of long-term outcome should ideally be blinded to the predictor and clinical details of the patient.
2. Multicenter studies will facilitate recruitment and a larger sample size.
3. Standardized definitions of the predictor should be used across studies. Clinical predictors should be explicitly defined. Accurate and objective techniques subject to the least interrater variance should be given preference. EEG findings, such as reactivity [120, 126] and electrographic/electroclinical status epilepticus [120, 128], should be standardized on the basis of published criteria and parameters of testing. Imaging-based studies should use validated quantification techniques [99, 106]. Biomarkers should be measured with standardized laboratory techniques at specific time points.
4. All study participants should, ideally, undergo assessment of the predictor rather than a subpopulation selected at the discretion of the treating clinician.
5. Studies of clinical prediction models should report model calibration for predicted probabilities.
6. The impact of the self-fulfilling prophecy should be mitigated with the following measures:
  - Institutional clinical practice guidelines should encourage delaying neuroprognostication for at least 72 h following ROSC/rewarming in the absence of death by neurological criteria, severe premorbid illness, baseline disability, severe comorbid illness, multiorgan dysfunction, or prior DNAR status. In the context of prospective research, a 2-week period of supportive care prior to a decision on tracheostomy may further mitigate the impact of the self-fulfilling prophecy.
  - The predictor should ideally not be used systematically by treating clinicians to formulate a prognosis and counsel surrogates. This may not be feasible for common clinical predictors, such as the physical examination and EEG.
  - Clinicians should be blinded to predictors that are not a part of routine clinical care.

Individual clinical variables are unlikely to be reliable predictors in isolation. The only exceptions in our systematic review were the bilateral absence of the pupillary light response and the bilateral absence of N20 responses on SSEP testing. Clinical prediction models that incorporate multiple independent clinical, electrophysiological, and imaging predictors may therefore be optimal for neuroprognostication. The primary limitation of the most widely studied clinical prediction models in the setting of cardiac arrest is a lack of validation for the prediction of long-term outcomes. Several prediction models based on machine learning algorithms, including neural networks, have undergone preliminary evaluation and await larger multicenter validation studies for the prediction of long-term outcome [158–160]. Several brain injury biomarkers other than NSE, such as neurofilament light chain, tau protein, S100 calcium-binding protein B, glial fibrillary acidic protein, and ubiquitin C-terminal hydrolase L1, are currently under investigation and may demonstrate value in multimodal neuroprognostication following cardiac arrest [161].

These guidelines and the preponderance of the neuroprognostication literature focus on the prediction of poor outcome. However, analyses of predictor accuracy for good long-term neurological outcome should be routinely incorporated into future neuroprognostication research. Discussions with patient and family representatives also highlighted the importance of lesser-studied outcomes following cardiac arrest. Several studies have established that cognitive dysfunction, anxiety, depression, posttraumatic stress disorder, and impairment of health-related quality of life are common in long-term survivors of cardiac arrest [162–170]. However, in addition to examining predictors of impairments in these domains, future prospective studies should use standardized instruments and time points for evaluation and compare occurrence with that in an age- and sex-matched control population.

## Conclusions

These guidelines provide recommendations on the reliability of predictors of poor outcome in the context of counseling surrogates of comatose survivors of cardiac arrest and suggest broad principles of neuroprognostication (Figs. 2, 3). Few predictors were considered reliable or moderately reliable based on the available body of evidence.

### Endorsements

These guidelines were endorsed by the American Heart Association and the Society of Critical Care Medicine. The American Academy of Neurology affirms the value of these guidelines.

## Supplementary Information

Below is the link to the electronic supplementary material.

Supplementary file1 (PDF 738 kb)
