# Supplementary material for: Guidelines for Neuroprognostication in Comatose Adult Survivors of Cardiac Arrest
Source: Neurocrit Care. 2023 Mar 22;38(3):533–63. doi: 10.1007/s12028-023-01688-3 (PMC10241762; doi:10.1007/s12028-023-01688-3)
Supplement: Supplementary file 1 — Supplementary file1 (PDF 738 kb) [file 12028_2023_1688_MOESM1_ESM.pdf]

Supplementary appendix 1

Neurocritical Care Society (NCS) and Deutsche Gesellschaft für Neurointensivmedizin (DGNI) Neuroprognostication guidelines: Methodology

How to use these guidelines

These guidelines provide recommendations on the reliability of selected individual clinical variables and prediction models (both hereafter referred to as "predictors"). These predictors have been categorized as reliable, moderately reliable, or not reliable. We based this categorization on the GRADE criteria supporting the use of the predictor in the context of counseling, as well as effect size. This categorization led to the recommendation to either use or not use the predictor to formulate a prognosis, caveats to the use of the predictor, and suggested language during counseling (**Table 1,** at the end of this document).

A key distinction exists between a *reliable* predictor of outcome in the context of counseling surrogates of patients requiring neurocritical care and an *independent* predictor of outcome. An independent predictor fulfills one criterion—a statistically significant association with the outcome of interest in an appropriately conducted multivariate analysis. In clinical practice, independent predictors of outcome may be used in risk stratification, selection of patients for targeted treatment (such as chemotherapy regimens for cancer), or as building blocks of clinical prediction models.[1, 2] A reliable predictor in the context of counseling patients requiring neurocritical care or their family members must be independent, but also fulfil other criteria as described in the "Effect size (Predictor accuracy)" and "Evidence to Recommendation (EtoR)" sections.

Reliable predictors, for the purposes of these guidelines, may be used to formulate a prognosis when the appropriate clinical context is present in the absence of potential confounders. These are predictors with clear actionable thresholds or clinical/ radiographic definitions, and a low rate of error in prediction of poor outcomes and with at least moderate certainty in the body of evidence using GRADE criteria. When the prognosis is formulated on the basis of one or more reliable predictors the clinician may describe the outcome as "very likely" during counseling. Given the inherent limitations in

neuroprognostication research, the clinician must nevertheless acknowledge the presence of uncertainty- albeit low- in the prognosis. **Moderately reliable individual predictors** may be used for prognostication only when additional reliable or moderately reliable predictors are present, in addition to the appropriate clinical context as specified above. These are also predictors with clear, actionable thresholds or clinical/ radiographic definitions, and a low rate of error in prediction of poor outcomes, but with lower certainty in the body of evidence using GRADE criteria, often a result of smaller studies that result in imprecision. When the prognosis is formulated on the basis of multiple moderately reliable predictors the clinician may describe the outcome as "likely" during counseling, but must acknowledge "substantial" uncertainty in the prognosis. **Moderately reliable clinical prediction models** that generate predicted probabilities of outcomes, in contrast, may be used for prognostication during counseling in the absence of other reliable or moderately reliable predictors. However, it is recommended that the clinician describe the predicted probability of the outcome as "an objective estimate only, subject to considerable uncertainty". While the panelists recognize that predictors that do not meet the criteria to be described as reliable or moderately reliable are often used by clinicians in formulating their subjective impressions of prognosis, they have nevertheless been deemed **not reliable** for the purposes of these guidelines and cannot be formally recommended for prognostication on their own. Variables deemed not reliable however may be a component of reliable or moderately reliable prediction models.

Guideline panel

The NCS and DGNI recruited 20 professionals in neurocritical care, nursing, and pharmacy to create a panel of experts for the neurocritical condition considered; including a NCS-assigned GRADE methodologist in 2018. The guideline panel consisted of two to four content experts who served as the primary authors for each disease, an affected patient or family member who served as the public representative, and the content experts of the other seven disease states which included the two chairs of the entire neuroprognostication guidelines. The inclusion of neurointensivists, neurosurgeons, advanced practice providers (nurse practitioners), pharmacists, and a neuropalliative care expert on the guideline panel created a multidisciplinary collaboration. A statistician-epidemiologist with expertise in

neuroprognostication and medical decision making served as a consultant. The panel convened in monthly video-conferences, with in-person meetings at two consecutive annual meetings of the NCS and one annual meeting of DGNI. Additional small group video and telephone meetings occurred ad hoc among content experts, the chairs, the GRADE expert, and the public representative. Recommendations were voted on using online survey software (Google Forms, Google Inc., Mountain View, California, United States). Panelists were required to disclose all potential conflicts of interest prior to participation. Potential conflicts were reviewed by the co-chairs of the panel, as well as the NCS guidelines committee, and managed in accordance with policies of NCS.

Selection of guideline questions

An initial Patient, Intervention, Comparison, Outcome and Time (PICOTS) question was framed to help identify candidate predictors: *"What are the reliable predictors (prognostic factors, variables, tests, scores, and multivariable models), prior to and on admission as well as during the hospital course to predict patient outcome at different follow up time points following each specific disease?"*. A professional librarian executed a comprehensive literature search using search criteria appropriate to this initial question. Since this search was expected to generate a large volume of articles, additional criteria were used to select studies with a focus on reliability of predictors and appropriate outcomes. These criteria are described below in the "systematic review methodology" section. The content experts reviewed the full-text articles that fulfilled selection criteria. Candidate predictors were then selected by these experts and confirmed by the panel, based on clinical relevance, availability in daily clinical practice, AND the presence of an appropriate body of literature. Candidate predictors and prediction models were considered "clinically relevant" if, in the subjective opinion of the content experts and guideline chairs, the predictor or components of the prediction models were accessible to clinicians; although universal availability was not required AND likely to be considered by clinicians while formulating a prognosis. An appropriate body of literature was considered present for any predictor that fulfilled two criteria—1)

evaluated in at least two published studies that included a minimum of 35-100 subjects (number of patients required was dependent on the disease state: 35 for Guillain-Barré Syndrome; 50 for spinal cord injury and status epilepticus; 100 for cardiac arrest, traumatic brain injury, acute ischemic stroke, intracerebral hemorrhage, and subarachnoid hemorrhage) and 2) established as an independent predictor in a multivariate analysis. An appropriate body of literature was considered present for clinical prediction models with at least one external validation study of at least 35-100 patients (dependent on the disease state as above) in addition to the initial report on development of the model (also with a minimum of 35-100 patients).

Selection of outcomes

Outcomes relevant to the question and each disease were identified by the panel, then rated by the primary content experts and the public representative. The outcomes were rated using the GRADE 1-9 scale and the score averaged. An outcome with an average score of greater than 6 was considered "critical" and included in the evaluation. The range of outcomes and the time period of outcome assessment was specific for each disease. The only outcomes included at the time of discharge from the acute care facility were mortality at discharge and beyond for most disease states and the need for mechanical ventilation with 14 days of onset of Guillain-Barré-Syndrome. Other outcomes considered were required to be assessed after a disease-specific longer time period for recovery and beyond. While a longer duration from time of injury to outcome assessment is ideal to capture the entirety of functional recovery, this prioritization may result in loss to follow-up. Significant loss to follow-up in observational studies may result in a selection bias, based on the patients most likely to respond or return to the index hospital for further medical care.

Systematic review methodology

Since the literature on prognosis was expected to be heterogenous, a narrative systematic review was performed. As described earlier, the initial librarian search string was appropriate to the question *"What*

are the reliable predictors (prognostic factors, variables, tests, scores, and multivariable models), prior to and on admission as well as during the hospital course to predict patient outcome at different follow up time points following each specific disease ?", to identify candidate predictors. Screening of articles was completed using DistillerSR software (Evidence Partners, Ottawa, Canada).

Abstract screening (Level I) was performed with the question "Does this study address prognostication in the focus disease (Yes/ No)?". Pediatric (<16 years) and animal studies were excluded, as were studies evaluating the efficacy of a therapeutic intervention.

Full text screening (Level II) was performed with the following exclusion criteria:

- Sample size less than 35/50/100 patients depending on the prevalence of the disease,
- Studies confined to a mild form the disease,
- Studies focused on a highly selected subgroup (for example penetrating traumatic brain injury, traumatic cardiac arrest, paraneoplastic Guillain-Barré-Syndrome, etc.),
- Studies focused entirely on genetic polymorphism,
- Studies that include multiple disease states and without an adequate sample size and separate analysis of the disease of interest,
- Studies of an intervention,
- Studies with neuroimaging not consistent with contemporary standards used to confirm the diagnosis,
- Studies that did not include one of the selected outcomes for the specific disease,
- Studies of predictors not established as independent with appropriate multivariate analysis,
- Studies of clinical prediction models that did not report model discrimination,
- Studies of laboratory biomarkers were included only if the biomarker was considered clinically relevant, and had been evaluated in more than one published study that met other criteria.

Data extraction and assessment for risk of bias (RoB) and was then performed only for studies that addressed the PICOTS question for each selected candidate predictor AND fulfilled full-text selection criteria. The Quality in Prognostic Studies (QUIPS) RoB instrument was used to evaluate studies of individual prognostic variables,[3] and the Prediction model Risk Of Bias ASsessment Tool (PROBAST) instrument used to evaluate studies of clinical prediction models.[4, 5] In addition to the standard domains of these RoB instruments, studies were evaluated for the risk of bias related to the self-filling prophecy with an additional domain that included three questions- whether a treatment suspension policy was used in the study, whether clinicians were blinded to the predictor and whether the predictor was systematically utilized by clinicians for prognostication during the time period of the study. Following assessment of risk of bias in each domain, an overall risk of bias- *high, moderate or low*- was assigned to each study.

Following data extraction and assessment of risk of bias of individual studies, a GRADE evidence profile with summary of findings table was constructed. Statistical measures of effect size varied across studies and were recorded in narrative form in the summary of findings. Decisions to downgrade the body of evidence for risk were based on review of risk of bias across all individual studies for the PICOTS question. The body of evidence for a specific predictor was downgraded for inconsistency only when the results of studies with approximately equal risk of bias were in conflict, with some studies suggesting the predictor was reliable and other studies showing a lack of statistical significance or a high rate of error. The evidence was downgraded for indirectness when population, predictor, outcome, or time of assessment varied significantly from the specifications of the PICOTS question. Downgrading of the evidence for imprecision was applied when the estimated confidence intervals were thought to be excessive for the clinical question. Publication bias could not be meaningfully evaluated, and the GRADE reasons to upgrade were not thought to be applicable to this body of evidence. Judgments on risk of bias, indirectness, inconsistency and imprecision were inherently subjective and focused on the respective prognostication question.

Effect size (Predictor accuracy)

Predictor accuracy is often described using measures such as the odds ratio (OR), which measures the *relative* probability of the outcome when the predictor is present, compared to the probability of the outcome in the absence of the predictor. In the context of neuroprognostication, predictors of good and poor outcomes are considered. However, the absolute probability of the outcome when the predictor is present is also important. For example, an older patient may be statistically more likely to suffer a poor outcome than a younger patient, but a large proportion of older individuals may nevertheless have a good outcome.[6] While surrogates of neurocritical care patients may be interested in factors that increase the probability of poor outcome, the absolute probability of a poor outcome is most likely used to make decisions on escalation or withdrawal of treatment measures. The performance of a clinical prediction model is evaluated using its ability to discriminate binary (good vs poor) outcomes, with measures such as the c-statistic or area under the receiver operating characteristic curve (AUC). Another important measure is model calibration, or the ability to correctly specify the probability of an outcome. Model calibration is typically reported as a goodness of fit, often using the Hosmer-Lemeshow test, or with a calibration curve, slope or intercept.[7]

Evidence to recommendation criteria

The GRADE Evidence-to-Recommendation criteria encompassed the overall quality of evidence, balance between desirable and undesirable outcomes, confidence in values and preferences, and resource use. Common principles were established for each disease state by the guideline panel in consultation with the patient or family representative, when considering these criteria.

Good practice statements

During literature review, the content experts recognized a lack of direct evidence to support the response to the PICOTS question for the specific disease, often because of insufficient clinical equipoise. Therefore, the panel decided to provide good clinical practice statements. Explicit statement of these clinical practice principles was considered essential to provide context as well as appropriate guidance.

REFERENCES:

1. Hemingway, H., Croft, P., Perel, P., et al. Prognosis research strategy (PROGRESS) 1: a framework for researching clinical outcomes. BMJ 2013;346:e5595.
2. Riley, R.D., Hayden, J.A., Steyerberg, E.W., et al. Prognosis Research Strategy (PROGRESS) 2: prognostic factor research. PLoS Med 2013;10(2):e1001380.
3. Hayden, J.A., van der Windt, D.A., Cartwright, J.L., Cote, P.Bombardier, C. Assessing bias in studies of prognostic factors. Ann Intern Med 2013;158(4):280-6.
4. Moons, K.G.M., Wolff, R.F., Riley, R.D., et al. PROBAST: A Tool to Assess Risk of Bias and Applicability of Prediction Model Studies: Explanation and Elaboration. Ann Intern Med 2019;170(1):W1-W33.
5. Wolff, R.F., Moons, K.G.M., Riley, R.D., et al. PROBAST: A Tool to Assess the Risk of Bias and Applicability of Prediction Model Studies. Ann Intern Med 2019;170(1):51-8.
6. Gattellari, M., Goumas, C., Garden, F.Worthington, J.M. Relative survival after transient ischaemic attack: results from the Program of Research Informing Stroke Management (PRISM) study. Stroke 2012;43(1):79-85.
7. Van Calster, B., McLernon, D.J., van Smeden, M., et al. Calibration: the Achilles heel of predictive analytics. BMC Med 2019;17(1):230.

Table 1: Reliable and moderately reliable predictors

Category of predictor/ model	GRADE criteria					Point estimates of accuracy in the body of evidence	Use during counseling of patients or surrogates?	Presence of additional specific reliable or moderately predictors required for use during counseling?	Suggested language during counseling of patients or surrogates	
	Risk of Bias	Inconsistency	Imprecision	Indirectness	Quality of Evidence-Overall				Likelihood of outcome	Disclaimer of Uncertainty during counseling
Reliable	One downgrade permitted	Downgrade NOT permitted	Downgrade NOT permitted	Downgrade NOT permitted	Moderate or High	High	Yes	Preferred, but not absolutely required	"Very likely"	Present, but low
Moderately reliable individual predictors	One downgrade permitted	Downgrade NOT permitted	One downgrade permitted	One downgrade permitted	Any	High	Yes	Yes	"Likely"	Substantial
Moderately reliable clinical prediction models	One downgrade permitted	Downgrade NOT permitted	One downgrade permitted	One downgrade permitted	Any	High.	Yes	No	Use predicted probability of outcome	"The predicted probability is an estimate, subject to considerable uncertainty"
Not reliable	One downgrade permitted	Downgrade NOT permitted	One downgrade permitted	One downgrade permitted	Any	Any	*No	Not applicable	Not applicable	Not applicable

- * Many predictors designated "not reliable" are practically utilized by clinicians in formulating and communicating real-world subjective impressions of prognosis. The purpose of these guidelines is to identify predictors, if any, that meet reliable or moderately reliable criteria.

Supplementary Appendix 2

GRADE Evidence Profile/ Summary of Findings table: Neuroprognostication- Cardiac arrest

Individual predictors- Mortality

Outcome	Predictor	Quality of Evidence					Summary of Findings (Narrative of effect size)
		Risk of Bias	Inconsistency	Indirectness	Imprecision	Quality of Evidence- Summary	
Mortality	Age	↓↓	↓			Very low	Point estimate of odds ratio for poor outcome 1.02 – 2.23. Point estimate of odds ratio for survival 0.78-0.97
Mortality	Cardiac rhythm- nonshockable	↓↓	↓			Very low	Point estimate of odds ratio for poor outcome 1.4 – 3.65. False positive rate 16%
Mortality	Time to return of spontaneous circulation	↓↓	↓			Very low	Point estimate of odds ratio for poor outcome 1.03 – 1.10. False positive rate 24%.
Mortality	Neurological examination≥ 72 hours from ROSC - Bilateral absence of the pupillary reflex.	↓↓			↓	Very low	Point estimate of odds ratio for poor outcome 2.53- 10.20 for the pupillary reflex. Insufficient evidence for the corneal reflex as a predictor of mortality.
Mortality	Neurological examination ≥ 72 hours from ROSC - Motor response	↓↓				Low	False positive rate 0-24%.
Mortality	Myoclonus ≤48 hours from ROSC						Insufficient evidence
Mortality	Computed Tomography (CT) ≥ 72 hours from ROSC- quantification of grey white ratio						Insufficient evidence
Mortality	Magnetic Resonance Imaging (MRI)- quantification of ADC 2-7 days from ROSC.						Insufficient evidence
Mortality	Electro- encephalography (EEG) ≥ 72						Insufficient evidence

	hours from ROSC with suppressed or burst suppression background, with or without periodic discharges						
Mortality	Somatosensory evoked potential (SSEP)						Insufficient evidence
Mortality	Neuron Specific Enolase (NSE)	↓↓	↓			Very low	AUC 0.81-0.88. False positive rate 27% for NSE>33 and 15% for NSE>47.8

<u>Prediction models- Mortality</u>

**Outcome**	**Prediction models**	**Quality of Evidence**					**Summary of Findings (<u>Narrative of effect size</u>)**
		**RoB**	**Inconsistency**	**Indirectness**	**Imprecision**	**QoE-Summary (High/ Moderate/ Low/ Very Low)**	
Mortality	OHCA	↓				Moderate	AUC 0.79-0.85. OHCA score >60: Sensitivity about 2%, Specificity 100% for mortality. No evidence of miscalibration when reported.
Mortality	CAHP	↓				Moderate	AUC 0.81-0.84. CAHP >200: Sensitivity about 39%, Specificity about 91% for mortality. No evidence of miscalibration when reported.
Mortality	GOFAR	↓				Moderate	Insufficient evidence- no studies with calibration reported. Single study reported that in patients with GO-FAR >= 24 (Very low survival)- 3/60 (5%) survived. Among patients with GO-FAR 14–23 (Low survival)- 5/90 (5.5%) survived.

Summary of recommendations- predictors of MORTALITY

**Predictors of MORTALITY at discharge or later**
**Age, Cardiac Rhythm & Time to return of spontaneous circulation**
When counseling surrogates of comatose survivors of cardiac arrest, we **suggest** the patient’s age alone **not be considered a reliable predictor** of mortality assessed at discharge or later. (weak recommendation; very low quality evidence)
When counseling family members and/ or surrogates of comatose survivors of cardiac arrest, we **suggest** the initial cardiac rhythm alone **not be considered a reliable predictor** of mortality assessed at discharge or later. (weak recommendation; very low quality evidence)
When counseling family members and/ or surrogates of comatose survivors of cardiac arrest, we **suggest** the time to return of spontaneous circulation (ROSC) alone **not be considered a reliable predictor** of mortality assessed at discharge or later. (weak recommendation; very low quality evidence)
**Neurological examination**
When counseling family members and/ or surrogates of comatose survivors of cardiac arrest, we **suggest** the bilateral absence of a pupillary light response, assessed at least 72 hours from ROSC, **not be considered a reliable predictor** of mortality assessed at discharge or later. (weak recommendation; very low quality evidence)
When counseling family members and/ or surrogates of comatose survivors of cardiac arrest, we **suggest** the bilateral absence of the corneal reflex alone, assessed at least 72 hours from ROSC, **not be considered a reliable predictor** of mortality assessed at discharge or later. (weak recommendation; very low quality evidence)
When counseling family members and/ or surrogates of comatose survivors of cardiac arrest, we **suggest** that an absent or extensor best-motor response alone, assessed at least 72 hours from ROSC (or 72 hours from rewarming, in patients treated with therapeutic hypothermia) **not be considered a reliable predictor** of mortality assessed at discharge or later. (weak recommendation; low quality evidence)
Myoclonus <48 hours from return of spontaneous circulation, in the absence of concomitant EEG evaluation- There is insufficient evidence for a recommendation
**Brain imaging**
Diffuse pattern of loss of grey-white differentiation with sulcal effacement on non-contrast computed tomography (CT) imaging of the brain performed at least 48 hours from return of spontaneous circulation- There is insufficient evidence for a recommendation
Diffuse pattern of restricted diffusion across vascular distributions in the bilateral cerebral cortex and deep grey matter on magnetic resonance imaging (MRI) of the brain performed between 2-7 days from ROSC - There is insufficient evidence for a recommendation
**Electrodiagnostic**
Suppressed or burst suppression background, with or without periodic discharges, on EEG performed at least 72 hours from ROSC (or 72 hours from rewarming, in patients treated with therapeutic hypothermia)- There is insufficient evidence for a recommendation
Bilateral absence of the N20 wave, with preservation of responses at Erb’s point and the cervical spine, on somatosensory evoked potential (SSEP) testing performed at least 48 hours from return of spontaneous circulation- There is insufficient evidence for a recommendation
**Biomarkers**
When counseling surrogates of comatose survivors of cardiac arrest, we **suggest** that the serum level of Neuron Specific Enolase (NSE) alone, measured ≤72 hours from return of spontaneous circulation,

**not be considered a reliable predictor** of mortality assessed at discharge or later. (*weak recommendation; very low quality evidence*)
**Prediction models**
When counseling surrogates of comatose survivors of cardiac arrest, we **suggest** that the Out of Hospital Cardiac Arrest (OHCA) prediction model **not be considered a reliable predictor** of mortality assessed at discharge or later. (*weak recommendation; moderate quality evidence*)
When counseling surrogates of comatose survivors of cardiac arrest, we **suggest** that the Cardiac Arrest Hospital Prognosis (CAHP) prediction model **not be considered a reliable predictor** of mortality assessed at discharge or later. (*weak recommendation; moderate quality evidence*)
When counseling surrogates of comatose survivors of cardiac arrest, we **suggest** that the Good Outcome Following Attempted Resuscitation (GOFAR) prediction model **not be considered a reliable predictor** of mortality assessed at discharge or later. (*weak recommendation; moderate quality evidence*)

Recommendation table: Individual prognostic indicators of mortality at discharge or later

PREDICTOR	TIME OF ASSESSMENT	RELIABILITY
Age	On admission	Not reliable
Initial cardiac rhythm- Nonshockable	On admission	Not reliable
Time to return of spontaneous circulation (ROSC)	On admission	Not reliable
Bilateral absence of the pupillary light response	At least 72 hours from ROSC	Not reliable
Bilateral absence of the corneal reflex	At least 72 hours from ROSC	Not reliable
Best motor response- extensor or absent	At least 72 hours from ROSC or rewarming	Not reliable
Myoclonus (clinical only, no EEG)	Within 48 hours of ROSC	Insufficient evidence
CT Diffuse loss of grey-white differentiation with sulcal effacement	At least 72 hours from ROSC	Insufficient evidence
MRI Diffuse pattern of restricted diffusion across vascular distributions in the bilateral cerebral cortex and deep grey matter	2-7 days from ROSC	Insufficient evidence
EEG- suppression or burst suppression with or without periodic discharges	At least 72 hours from ROSC or rewarming	Insufficient evidence
SSEP- bilateral absence of N20 waves	At least 48 hours from ROSC	Insufficient evidence
Neuron Specific Enolase (NSE)	Within 72 hours of ROSC	Not reliable

Clinical prediction models for mortality at discharge or later

CLINICAL PREDICTION MODEL	RELIABILITY
Good Outcome Following Attempted Resuscitation (GOFAR)	Not reliable
Out of Hospital Cardiac Arrest (OHCA)	Not reliable
Cardiac Arrest Hospital Prognosis (CAHP)	Not reliable

Supplementary appendix 3: Cerebral Performance Category and modified Rankin scale

Cerebral Performance Categories (CPC) Scale

Safar P. Resuscitation after Brain Ischemia, in Grenvik A and Safar P Eds: Brain Failure and Resuscitation, Churchill Livingstone, New York, 1981; 155-184.

Note: If patient is anesthetized, paralyzed, or intubated, use "as is" clinical condition to calculate scores.

CPC 1- Good cerebral performance: conscious, alert, able to work, might have mild neurologic or psychologic deficit.

CPC 2- Moderate cerebral disability: conscious, sufficient cerebral function for independent activities of daily life. Able to work in sheltered environment.

CPC 3- Severe cerebral disability: conscious, dependent on others for daily support because of impaired brain function. Ranges from ambulatory state to severe dementia or paralysis.

CPC 4- Coma or vegetative state: any degree of coma without the presence of all brain death criteria. Unawareness, even if appears awake (vegetative state) without interaction with environment; may have spontaneous eye opening and sleep/awake cycles. Cerebral unresponsiveness.

CPC 5- Brain death: apnea, areflexia, EEG silence, etc.

Modified Rankin Scale

Van Swieten, J C; Koudstaal, P J; Visser, M C; Schouten, H J; van Gijn, J (May 1988). "Interobserver agreement for the assessment of handicap in stroke patients". Stroke. 19 (5): 604–607.

0- The patient has no residual symptoms.

1- The patient has no significant disability; able to carry out all pre-stroke activities.

2- The patient has slight disability; unable to carry out all pre-stroke activities but able to look after self without daily help.

3 - The patient has moderate disability; requiring some external help but able to walk without the assistance of another individual.

4 - The patient has moderately severe disability; unable to walk or attend to bodily functions without assistance of another individual.

5- The patient has severe disability; bedridden, incontinent, requires continuous care.

6- The patient has expired (during the hospital stay or after discharge from the hospital).

Supplementary Appendix 4

Librarian search string

Database: All Ovid Medline <1946 - present>

Search Strategy:

----

1 exp Heart Arrest/ (44223)

2 (arrest* adj3 cardiac).ti,ab. (30880)

3 (arrest* adj3 cardiopulmonary).ti,ab. (3048)

4 (arrest* adj3 heart).ti,ab. (1702)

5 asystole*.ti,ab. (3513)

6 cardiorespiratory arrest*.ti,ab. (923)

7 circulatory arrest.ti,ab. (5385)

8 anoxic encephalopath*.ti,ab. (304)

9 ischemic encephalopath*.ti,ab. (2659)

10 exp Hypoxia, Brain/ and *Coma/ (224)

11 ((anoxic or hypoxic) adj3 coma).ti,ab. (183)

12 1 or 2 or 3 or 4 or 5 or 6 or 7 or 8 or 9 or 10 or 11 (68845)

13 Disease Progression/ (145228)

14 exp "OUTCOME ASSESSMENT (HEALTH CARE)"/ (977947)

15 outcome*.ti,ab,kf. [title, abstract, author supplied keyword] (1498262)

16 "Predictive Value of Tests"/ (187455)

17 exp PROGNOSIS/ (1478486)

18 prognostic.tw. (257337)

19 or/13-18 (2823645)

20 12 and 19 (21786)

21 incidence.sh. or exp mortality/ or follow-up studies.sh. or prognos:.tw. or predict:.tw. or course:.tw. [validated hedge for prognosis from the HIRU at McMaster University] (3162483)

22 12 and 21 (20118)

23 20 or 22 (30497)

24 "Analysis of Variance"/ [includes Multivariate Analysis] (212630)

25 outcome variable.mp. (6338)

26 Multivariate.tw. (292572)

27 variable*.ti,ab. (724641)

28 variance*.ti,ab. (169055)

29 Data Interpretation, Statistical/ (54504)

30 statistic*.tw. (971228)

31 sn.fs. [statistics and numerical data as a floating subheading] (722114)

32 Data Interpretation, Statistical/ (54504)

33 24 or 25 or 26 or 27 or 28 or 29 or 30 or 31 or 32 (2666481)

34 23 and 33 (7439)

35 exp cohort studies/ [includes: follow-up studies/, longitudinal studies/, prospective studies/, retrospective studies/, controlled before-after studies/, cross-sectional studies/, or historically controlled study/] (1816568)

36 (Follow-up or longitudinal or prospective or retrospective or before-after or cross-sectional or controlled).tw. (2586857)

37 (comparative study or evaluation study or meta analysis).pt. (1907158)

38 35 or 36 or 37 (4827690)

39 34 and 38 (5348)

40 *PEDIATRICS/ (35995)

41 animals/ not (humans/ and animals/) (4506319)

42 40 or 41 (4542275)

43 39 not 42 (5206)

44 limit 43 to english language (4970)

45 remove duplicates from 44 (4962)

Supplementary Appendix 5: Individual studies — Age as a predictor of functional outcome

FIRST AUTHOR LAST NAME	PMID	PROGNOSTIC FACTOR(S) EVALUATED	Prevalence % of the predictor	OUTCOME(S)	Sample size for the outcome	Prevalence % of the outcome	EFFECT SIZE WITH 95% CI as reported in the study (False Positive Rate, Sensitivity, Specificity, Odds Ratio, Relative Risk)	OVERALL RISK OF BIAS FOR THE STUDY	Overall risk of bias: Comments
Edgren 1989	2655364	Age >65	NA	GOOD functional outcome 1 year	262	0.17	Not significant	High	Study participation high, Study attrition moderate, Study confounding moderate, Self fulfilling prophecy high
Wu 2011	21330629	Age	Continuous	mRS>4 at 6 months	151	0.91	p=0.49 in univariate, not significant in multivariate analysis	High	Study participation moderate, Self-fulfilling prophecy high
Hayashida 2014	25168063	Age	continuous	CPC 1-2 at 3 months	495	0.15	OR 0.98 (0.95-0.99)	Low	
Thomsen 2015	25619443	Age	continuous	CPC 3-5 at 6 months	234	Not available	OR every 5 years of age- 1.21 (1.05-1.4), p<0.01	High	Self-fulfilling prophecy high
Kragholm 2015	25941005	Age 1-49 vs 50-65 years		return to work	796 survivors to day 30	0.77 of survivors at day 30	HR 1.31 (1.02-1.68)	High	Study confounding high, Self-fulfilling prophecy high
Stammet 2015	25975474	Age	continuous	CPC 3-5 at 6 months	686	0.49	OR 1.098 (1.073-1.124)	Moderate	Study participation moderate, Self-fulfilling prophecy moderate. TTM trial substudy.
Hifumi 2015	26212234	Age >65	0.38	CPC 1-2 at 90 days	302	0.57	OR 0.30 (0.17-0.53), p<0.01	Low	

Thomsen 2016	26468897	Age	continuous	CPC 3-5 at 6 months	447	Not available	OR 1.06 (1.04-1.08), p<0.0001	Moderate	Study participation moderate, Self-fulfilling prophecy moderate. TTM trial substudy.
Devaux 2016	27438111	Age	continuous	CPC 3-5 at 6 months	579	0.48	OR 2.90 (2.14-3.94)	Moderate	Study participation moderate, Self-fulfilling prophecy moderate. TTM trial substudy.
Grand 2016	27553102	Age	continuous	CPC 3-5 at 6 months	602	Not available	OR 1.38 (1.24-1.52), p<0.0001	Moderate	Study participation moderate, Self-fulfilling prophecy moderate. TTM trial substudy.
Frydland 2016	27614855	Age	continuous	CPC 3-5 at 6 months	647	Not available	OR 1.06 (1.04-1.08), p<0.0001	Moderate	Study participation moderate, Self-fulfilling prophecy moderate. TTM trial substudy.
Frydland 2016	27614855	Age	continuous	mRS 4-6 at 6 months	647	Not available	OR 1.06 (1.04-1.08), p<0.0001	Moderate	Study participation moderate, Self-fulfilling prophecy moderate. TTM trial substudy.
Lee 2018	29763714	Age	continuous	CPC 3-5 at 6 months	329	0.70	OR 1.06 (1.03-1.092)	Low	
Ebner 2019	30691510	Age	continuous	CPC 3-5 at 6 months	869	0.49	OR 1.07 (1.05-1.09), p<0.001	Moderate	Study participation moderate, Self-fulfilling prophecy moderate. TTM trial substudy.
Pekkarinen 2019	30819234	Age	Continuous	CPC 3-5 at 12 months	1324	0.55	OR 1.03 (95%CI 1.02 - 1.04), p< 0.01	High	Self fulfilling prophecy high
Han 2019	30934011	Age	Continuous	CPC 1-2 at 3 months	335	0.14	OR 0.988 (95%CI 0.970–1.006), p=0.186	High	Outcome measurement high

Hong 2019	30953628	Age	Continuous	CPC 3-5 at 6 months	512	0.68	OR 1.058 (95%CI 1.027 1.089), p< 0.001	Low	
Ruijter 2019	31155751	Age	Continuous	CPC 3-5 at 6 months	850	0.54	Beta coefficient 0.039 (SE 0.010), p<0.001	High	Study participation moderate, Self-fulfilling prophecy high
Ruijter 2019	31155751	Age	Continuous	CPC 1-2 at 6 months	850	0.46	Beta coefficient −0.028 (SE 0.009), p=0.003	High	Study participation moderate, Self-fulfilling prophecy high
Oh 2019	31521016	Age	Continuous	mRS<4 at 6 months	583	0.35	OR .98 (95%CI 0.96-1.00), p=0.1	Low	
Nakstad 2020	31926258	Age	Continuous	CPC 3-5 at 6 months	259	0.51	p=0.095	High	Study confounding moderate, Self fulfilling prophecy high
Deye 2020	32858156	Age	Continuous	CPC 3-5 at 3 months	330	0.67	p=0.0002 in univariate analysis	High	Self fulfilling prophecy high

Age as a predictor of mortality

FIRST AUTHOR LAST NAME	PMID	PROGNOSTIC FACTOR(S) EVALUATED	Prevalence % of the predictor	OUTCOME(S)	Sample size for the outcome	Prevalence % of the outcome	EFFECT SIZE WITH 95% CI as reported in the study (False Positive Rate, Sensitivity, Specificity, Odds Ratio, Relative Risk)	OVERALL RISK OF BIAS FOR THE STUDY	Overall risk of bias: Comments
Edgren 1989	2655364	Age >65	NA	Mortality 1 year	262	0.79	p=0.009	High	Study participation high, Study attrition moderate, Study confounding moderate, Self fulfilling prophecy high
Grubb 1995	7623574	Age	Continuous	Mortality in-hospital, all-cause	270	0.51	NS	High	Self fulfilling prophecy high
Arrich 2006	16974213	age	continuous	in-hospital mortality	774	0.56	OR 1.05 (1.04-1.07)	High	Self fulfilling prophecy high
Chan 2013	23484828	Age 75-84 compared to 65-74	Continuous	Survival 1 year	6972	0.42	OR 0.92 (95% CI 0.88 to 0.96), p<0.001	High	Study participation High, Self-fulfilling prophecy High

Age as a predictor of mortality

Chan 2013	23484828	Age >85 compared to 65-74	Continuous	Survival 1 year	6972	0.42	OR 0.78 (95% CI 0.73 to 0.84), p<0.001	High	Study participation High, Self-fulfilling prophecy High
Roberts 2013	23507719	Age	Continuous	Mortality in-hospital, all-cause	203	0.67	OR 1.29 (95% CI 1.02 to 1.62)	High	Self-fulfilling prophecy high
Bro-Jeppesen 2013	23917079	age	continuous	Mortality at 30 days	270	0.29	OR 1.20 (1.1-1.4)	High	Study participation moderate, Self fulfilling prophecy high
Thomsen 2015	25619443	age	continuous	mortality 6 mo	234	0.28	OR 1.19 (1.05-1.36)	High	Self-fulfilling prophecy high
Thomsen 2016	26468897	age (both)	continuous	mortality 6 months	447	0.47	OR 1.04 (1.03-1.06)	Moderate	Study participation moderate, Self-fulfilling prophecy moderate. TTM trial substudy.
Devaux 2016	27438111	Age (both poor outcome and death)	continuous	mortality 6 months	579	Not available	OR 1.75 (1.49-2.05)	Moderate	Study participation moderate, Self-fulfilling prophecy moderate. TTM trial substudy.

Age as a predictor of mortality

Grand 2016	27553102	age	continuous	mortality 6 months	602	Not available	OR 1.25 (1.17-1.33)	Moderate	Study participation moderate, Self-fulfilling prophecy moderate. TTM trial substudy.
Wada 2016	27679536	Age	Continuous	Mortality in-hospital, all-cause	388	0.40	Not statistically significant	High	Self-fulfilling prophecy high
Russo 2018	28045336	Age	continuous	hospital death	122	0.24	OR 1.07 (1.02-1.11)	High	Self-fulfilling prophecy high
Lee 2017	28392372	Age	continuous	6 month mortality	534	0.60	OR 1.05 (1.03-1.07)	Low	
Nayeri 2017	28589506	Age	Continuous	Mortality 1 year	314	0.62	OR 1.03 (95% CI 1.01 to 1.05), p=0.014	High	Self-fulfilling prophecy high
Salam 2018	29553891	Age in shockable	continuous	30 day mortality	324	0.62	OR 1.26 (1.16-1.36)	High	Self-fulfilling prophecy high
Limpawattana 2018	29593417	Age	Continuous	Mortality 1 year	202	0.92	Hazard ratio 0.99 (95%CI 0.98-1.00), p=0.370 in univariate analysis	Moderate	Self fulfilling prophecy moderate
Sinkovic 2018	29854815	Age	Continuous	Mortality 6 month	119	0.48	Adjusted OR per 10 years of age 1.56 (95%CI 0.92 to 2.63), p=0.097	High	Study participation moderate, Study attrition moderate, Self-fulfilling prophecy high

Age as a predictor of mortality

Smith 2019	30411470	Age >=50	0.83	Mortality in-hospital, all-cause	388	0.62	OR 2.23, p=0.04	High	Self fulfilling prophecy high
Patel 2019	30815378	Age	Continuous	Mortality in-hospital, all-cause	153	0.65	OR 1.03 (95%CI 1.0-1.07), p=0.05	High	Statistical analysis high, Self fulfilling prophecy High
Pekkarinen 2019	30819234	Age	Continuous	Mortality 1 year	1324	0.48	OR 1.03 (95%CI 1.02 - 1.04), p< 0.01	High	Self fulfilling prophecy high
McLaughlin 2019	31103131	Age	Continuous	Mortality in-hospital, all-cause	1433	0.47	Hazard ratio 1.03 (95% CI 1.03-1.04), p<0.001	High	Self fulfilling prophecy high
Kong 2020	31306348	Age	Continuous	Survival in-hospital, all-cause	524	0.70	OR 0.968 (95%CI 0.946–0.989), p=0.004	Moderate	Self fulfilling prophecy moderate
Cocchi 2020	31466497	Age	Continuous	Mortality in-hospital, all-cause	249	0.61	Odds ratio (per year increase) 1.02 (95% CI 1.00-1.04), p=0.18	High	Self fulfilling prophecy high
Oh 2019	31521016	Age	Continuous	Survival 6 months	583	0.45	NS in multivariate analysis	Moderate	Self fulfilling prophecy moderate
Fisher 2021	33021889	Age	Continuous	Mortality in-hospital, all-cause	254	0.30	Cox proportional hazard ratio for death 1.02 (95% CI 1.01–1.03), p=0.0001	High	Self fulfilling prophecy high

Initial cardiac rhythm as a predictor of functional outcome

FIRST AUTHOR LAST NAME	PMID	PROGNOSTIC FACTOR(S) EVALUATED	Prevalence % of the predictor	OUTCOME(S)	Sample size for the outcome	Prevalence % of the outcome	EFFECT SIZE WITH 95% CI as reported in the study (False Positive Rate, Sensitivity, Specificity, Odds Ratio, Relative Risk)	OVERALL RISK OF BIAS FOR THE STUDY	Overall risk of bias: Comments
Edgren 1989	2655364	Cardiac rhythm-Nonshockable	0.39	GOOD functional outcome 1 year	262	0.17	Not statistically significant	High	Study participation high, Study attrition moderate, Study confounding moderate, Self fulfilling prophecy high
Reisinger 2007	17060343	Non shockable for persistent coma poor outcome at 6 mo (CPC 4)	0.47	CPC 4 at 6 months	177	0.33	OR 10.10 (2.3-44.24)	Moderate	Study confounding moderate, Self-fulfilling prophecy moderate
Rossetti 2010	20373341	Non-Shockable rhythm	0.4	CPC 3-5 at 3-6 months	109	0.77	FPR 0.16	High	Self fulfilling prophecy high
Greer 2013	23954666	Non-shockable rhythm	0.66	mRS 4-6 at 6 months	200	0.90	FPR 0.4 for all non-shockable-FPR 0.3 for PEA and 0.1 for asystole	High	Study confounding moderate, Self-fulfilling prophecy high
Terman 2014	25014063	Non-Shockable rhythm	0.46	CPC 1-2 at 6-12 months	123	0.29	OR 0.09 (0.09-0.3)	High	Self-fulfilling prophecy high

Initial cardiac rhythm as a predictor of functional outcome

Hayashida 2014	25168063	Shockable rhythm	0.24	CPC 1-2 at 3 months	495	0.15	OR 2.53 (1.10-5.79)	Low	
Devaux 2016	27438111	First rhythm (both)	0.79	CPC 3-5 at 6 months	579	0.53	OR 0.36 (0.18-0.76)	Moderate	Study participation moderate, Self-fulfilling prophecy moderate. TTM trial substudy.
Lee 2018	29763714	ECG rhythm in EMS nonshockable	0.69	CPC 3-5 at 6 months	329	0.70	OR 13.46 (1.725-105)	Low	
Lee 2018	29763714	ECG rhythm in ED nonshockable	0.78	CPC 3-5 at 6 months	329	0.70	OR 4.06 (1.077-15.3)	Low	
Ebner 2019	30691510	Shockable rhythm	Not available	CPC 3-5 at 6 months	869	0.49	OR 0.19 (0.11–0.34), p<0.001	Moderate	Study participation moderate, Self-fulfilling prophecy moderate. TTM trial substudy.
Pekkarinen 2019	30819234	Cardiac rhythm-Nonshockable	0.40	CPC 3-5 at 12 months	1324	0.55	OR 3.89 (95%CI 2.82 - 5.37), p< 0.01	High	Self fulfilling prophecy high
Hong 2019	30953628	Cardiac rhythm-Nonshockable	0.56	CPC 3-5 at 6 months	512	0.68	OR 6.767 (95%CI 1.540-29.727), p=0.011	Low	
Nakstad 2020	31926258	Cardiac rhythm-Nonshockable	0.32	CPC 3-5 at 6 months	259	0.51	FPR 0.13 (0.08-0.21)	High	Study confounding moderate, Self fulfilling prophecy high

Initial cardiac rhythm as a predictor of mortality

FIRST AUTHOR LAST NAME	PMID	PROGNOSTIC FACTOR(S) EVALUATED	Prevalence % of the predictor	OUTCOME(S)	Sample size for the outcome	Prevalence % of the outcome	EFFECT SIZE WITH 95% CI as reported in the study (False Positive Rate, Sensitivity, Specificity, Odds Ratio, Relative Risk)	OVERALL RISK OF BIAS FOR THE STUDY	Overall risk of bias: Comments
Edgren 1989	2655364	Cardiac rhythm-Nonshockable	0.39	Mortality 1 year	262	0.79	p=0.012	High	Study participation high, Study attrition moderate, Study confounding moderate, Self fulfilling prophecy high
Grubb 1995	7623574	Cardiac rhythm-Nonshockable	0.16	Mortality in-hospital, all-cause	197	0.51	p<0.001	High	Self fulfilling prophecy high
Rossetti 2010	20373341	Non-Shockable rhythm	0.4	Mortality in-hospital, all-cause	111	0.59	FPR 0.16	High	Self fulfilling prophecy high
Devaux 2016	27438111	First rhythm (both)	0.79	mortality 6 months	579	Not available	OR 0.5 (0.37-0.68)	Moderate	Study participation moderate, Self-fulfilling prophecy moderate. TTM trial substudy.

Initial cardiac rhythm as a predictor of mortality

Sinkovic 2018	29854815	Cardiac rhythm-Nonshockable	0.42	Mortality 6 month	119	0.48	Adjusted OR 1.40 (95%CI 0.33 to 5.88), p=0.647	High	Study participation moderate, Study attrition moderate, Self-fulfilling prophecy high
Pekkarinen 2019	30819234	Cardiac rhythm-Nonshockable	0.40	Mortality 1 year	1324	0.48	OR 3.65 (95%CI 2.61 - 4.82), p< 0.01	High	Self fulfilling prophecy high
McLaughlin 2019	31103131	Cardiac rhythm-Nonshockable	0.25	Mortality in-hospital, all-cause	1433	0.47	Hazard ratio 1.55 (95% CI 1.17-2.03), p=0.002	High	Self fulfilling prophecy high

Time to return of spontaneous circulation (ROSC) as a predictor of functional outcome

FIRST AUTHOR LAST NAME	PMID	PROGNOSTIC FACTOR(S) EVALUATED	Prevalence % of the predictor	OUTCOME(S)	Sample size for the outcome	Prevalence % of the outcome	EFFECT SIZE WITH 95% CI as reported in the study (False Positive Rate, Sensitivity, Specificity, Odds Ratio, Relative Risk)	OVERALL RISK OF BIAS FOR THE STUDY	Overall risk of bias: Comments
Edgren 1989	2655364	Time to ROSC- Untreated arrest time >5 mins	0.36	GOOD functional outcome 1 year	262	0.17	p<0.001	High	Study participation high, Study attrition moderate, Study confounding moderate, Self fulfilling prophecy high
Edgren 1989	2655364	Time to ROSC- CPR time >15 mins	0.45	GOOD functional outcome 1 year	262	0.17	p=0.033	High	Study participation moderate, Study attrition moderate, Study confounding moderate, Self fulfilling prophecy high
Rossetti 2010	20373341	ROSC >25 minutes	0.48	CPC 3-5 at 3-6 months	109	0.77	FPR 0.24	High	Self fulfilling prophecy high

Time to return of spontaneous circulation (ROSC) as a predictor of functional outcome

Laurikkala 2019	29608551	ROSC delay	Not reported	CPC 3-5 at 12 months	458	0.60	NS on multivariate analysis	Moderate	Study confounding moderate, Self-fulfilling prophecy moderate
Ebner 2019	30691510	Time to ROSC	continuous	CPC 3-5 at 6 months	869	0.49	OR 1.03 (1.02-1.04), p<0.001	Moderate	Study participation moderate, Self-fulfilling prophecy moderate. TTM trial substudy.
Pekkarinen 2019	30819234	ROSC delay (min)	Continuous	CPC 3-5 at 12 months	1324	0.55	OR 1.05 (95%CI 1.03 - 1.06), p< 0.01	High	Self fulfilling prophecy high
Han 2019	30934011	ACLS duration	Continuous	CPC 1-2 at 3 months	335	0.14	OR 0.965 (95% 0.947–0.984), p<0.001	High	Outcome measurement high
Oh 2019	31521016	Collapse to ROSC	Continuous	mRS<4 at 6 months	583	0.35	OR .96 (95%CI 0.94-0.98), p<0.001	Low	
Nakstad 2020	31926258	Time to ROSC >25 min	Not clearly stated	CPC 3-5 at 6 months	259	0.51	FPR 0.32 (0.24-0.42)	High	Study confounding moderate, Self fulfilling prophecy high

Time to return of spontaneous circulation (ROSC) as a predictor of functional outcome

Deye 2020	32858156	No flow time-collapse to onset of CPR	Continuous	CPC 3-5 at 3 months	330	0.67	OR 0.790 (95% CI 0.707-0.884), p<0.0001	High	Self fulfilling prophecy high

Time to return of spontaneous circulation (ROSC) as a predictor of mortality

FIRST AUTHOR LAST NAME	PMID	PROGNOSTIC FACTOR(S) EVALUATED	Prevalence % of the predictor	OUTCOME(S)	Sample size for the outcome	Prevalence % of the outcome	EFFECT SIZE WITH 95% CI as reported in the study (False Positive Rate, Sensitivity, Specificity, Odds Ratio, Relative Risk)	OVERALL RISK OF BIAS FOR THE STUDY	Overall risk of bias: Comments
Edgren 1989	2655364	Time to ROSC- Untreated arrest time >5 mins	0.36	Mortality 1 year	262	0.79	p=0.039	High	Study participation high, Study attrition moderate, Study confounding moderate, Self fulfilling prophecy high
Edgren 1989	2655364	Time to ROSC- CPR time >15 mins	0.45	Mortality 1 year	262	0.79	p=0.021	High	Study participation moderate, Study attrition moderate, Study confounding moderate, Self fulfilling prophecy high
Rossetti 2010	20373341	ROSC >25 minutes	0.49	Mortality in-hospital, all-cause	111	0.59	FPR 0.24	High	Self fulfilling prophecy high

Time to return of spontaneous circulation (ROSC) as a predictor of mortality

Lee 2017	28392372	Time to ROSC	continuous	6 month mortality	534	0.60	OR 1.03 (1.01-1.04)	Moderate	Self-fulfilling prophecy moderate
Nayeri 2017	28589506	Time to ROSC (min)	Continuous	Mortality 1 year	306	0.62	OR 1.06 (95% CI 1.03 to 1.08), p<0.001	High	Self-fulfilling prophecy high
Salam 2018	29553891	Time to ROSC with shockable rhythm	continuous	30 day mortality	324	0.28	OR 1.10 (1.03-1.18), p<0.01	High	Prognostic factor measurement moderate, Self-fulfilling prophecy high
Salam 2018	29553891	Time to ROSC with nonshockable rhythm	continuous	30 day mortality	324	0.62	OR 1.07 (0.98-1.18), p=0.11	High	Prognostic factor measurement moderate, Self-fulfilling prophecy high
Pekkarinen 2019	30819234	Time to ROSC (min)	Continuous	Mortality 1 year	1324	0.48	OR 1.05 (95%CI 1.03 - 1.06), p< 0.01	High	Self fulfilling prophecy high
Kong 2020	31306348	Arrest to ROSC time	Continuous	Survival in-hospital, all-cause	524	0.70	OR 0.934 (95%CI 0.913–0.957), p<0.001	Moderate	Self-fulfilling prophecy moderate
Oh 2019	31521016	Collapse to ROSC	Continuous	Survival 6 months	583	0.45	OR .96 (95%CI 0.95-0.98), p<0.001	Moderate	Self-fulfilling prophecy moderate

Pupillary light response and Corneal reflex as predictors of functional outcome

FIRST AUTHOR LAST NAME	PMID	Category of Predictor	Timing of prognostic factor evaluation	Prevalence % of the predictor	OUTCOME(S)	Sample size for the outcome	Prevalence % of the outcome	EFFECT SIZE WITH 95% CI as reported in the study (False Positive Rate, Sensitivity, Specificity, Odds Ratio, Relative Risk)	OVERALL RISK OF BIAS FOR THE STUDY	Overall risk of bias: Comments
Lee 2018	29763714	Pupillary reflex absent, bilateral	within 2 h of ROSC	0.80	CPC 3-5 at 6 months	329	0.70	FPR 0.15	High	Prognostic factor measurement high
Dragancea 2015	25921544	Pupillary light response absent, bilateral	Median 117 (93-137) hours	0.21	CPC 3-5 at 6 months	308	0.85	FPR 2.1% (0.3-11), Sensitivity 24.1% (19.3–29.7)	Moderate	Study participation moderate, Study confounding moderate. Self-fulfilling prophecy moderate. TTM trial substudy.

Pupillary light response and Corneal reflex as predictors of functional outcome

Edgren 1994	7909098	Pupillary light response absent, bilateral	72h from ROSC	0.07	CPC 3-5 at 6-12 months	262	0.54	FPR 0	High	Study confounding moderate, Statistical analysis moderate, Self fulfilling prophecy high
Greer 2013	23954666	Pupillary light response absent, bilateral	Day 3, Day 7	Day 3- 0.19, Day 7- 0.12	mRS 4-6 at 6 months	200	0.90	FPR 0 at both time points. Sensitivity 24% at day 3 and 18% at day 7.	High	Study confounding moderate, Self-fulfilling prophecy high
Hong 2019	30953628	Pupillary light response absent, bilateral	Within 2 hours of ROSC	0.52	CPC 3-5 at 6 months	512	0.68	OR 3.789, (95%CI 1.480-9.698), p=0.005	High	Prognostic factor measurement high
Levy 1985	3968772	Pupillary light response absent, bilateral	Initial examination	0.25	Poor functional outcome at 1 year- Severe disability (dependence for some functions) or worse	210	0.88	FPR 0 (0-0.07)	High	Study confounding moderate, Self fulfilling prophecy high

Pupillary light response and Corneal reflex as predictors of functional outcome

Moseby-Knappe 2018	30383090	Pupillary light response absent, bilateral	Median 119 hrs	0.21	CPC 3-5 at 6 months	245	0.86	Sensitivity 24%, Specificity 97%	Moderate	Study participation moderate, Study confounding moderate, Self-fulfilling prophecy moderate. TTM trial substudy.
Nakstad 2020	31926258	Pupillary light response absent, bilateral	>72h	0.06	CPC 3-5 at 6 months	185	0.51	FPR 0	High	Study confounding moderate, Self fulfilling prophecy high
Oh 2020	32169609	Pupillary light response absent, bilateral	72-96 hours	0.44	CPC 3-5 at 6 months	259	0.70	Sensitivity 62.2% (54.6-69.3), FPR 0.05 (0.01-0.12)	Moderate	Study confounding moderate
Scarpino 2021	33819501	Pupillary light response absent, bilateral	72h from ROSC	0.28	CPC 3-5 at 6 months	403	0.66	FPR 0.04 (0.01-0.11), sensitivity 66% (58-73)	Moderate	Study participation moderate, Study confounding moderate

Pupillary light response and Corneal reflex as predictors of functional outcome

Velly 2018	29500154	Pupillary light response absent, bilateral	On admission	0.18	CPC 3-5 at 6 months	150	0.78	FPR 0.18	High	Study participation high, Prognostic factor measurement high, Self-fulfilling prophecy high
Wu 2011	21330629	Pupillary light response absent, bilateral	72h from ROSC	0.19	mRS>4 at 6 months	151	0.91	Sensitivity 24% (16–34), Specificity 100% (73–100), significant in multivariate analysis	High	Study participation moderate, Self-fulfilling prophecy high
Rossetti 2010	20373341	Either pupillary, corneal or oculocephalic reflexes absent bilaterally	36-72 hours from ROSC	0.43	CPC 3-5 at 3-6 months	109	0.77	FPR 0.08	High	Self fulfilling prophecy high

Pupillary light response and Corneal reflex as predictors of functional outcome

Dragancea 2015	25921544	Corneal reflex absent, bilateral	Median 117 (93-137) hours	0.3	CPC 3-5 at 3-6 months	301	0.85	FPR 2.2% (0.3-11), Sensitivity 35% (29–41)	Moderate	Study participation moderate, Study confounding moderate, Self-fulfilling prophecy moderate. TTM trial substudy.
Greer 2013	23954666	Corneal reflex absent, bilateral	Day 3, Day 7	Day 3- 0.39, Day 7- 0.15	mRS 4-6 at 6 months	200	0.70	FPR 0 at both time points. Sensitivity 49% at day 3 and 23% at day 7.	High	Study confounding moderate, Self-fulfilling prophecy high
Hong 2019	30953628	Corneal reflex absent, bilateral	Within 2 hours of ROSC	0.64	CPC 3-5 at 6 months	512	0.68	OR 6.643 (95%CI 2.445-18.049), p=0.005	High	Prognostic factor measurement high
Lee 2018	29763714	Corneal reflex absent, bilateral	within 2 h of ROSC	0.80	CPC 3-5 at 6 months	329	0.70	FPR 0.26, OR 5.63 (2.08 - 15.20), p=0.001	High	Prognostic factor measurement high

Pupillary light response and Corneal reflex as predictors of functional outcome

Moseby-Knappe 2018	30383090	Corneal reflex absent, bilateral	Median 119 hrs	0.32	CPC 3-5 at 6 months	245	0.86	Sensitivity 37%, Specificity 97%	Moderate	Study participation moderate, Study confounding moderate, Self-fulfilling prophecy moderate. TTM trial substudy.
Oh 2020	32169609	Corneal reflex absent, bilateral	72-96 hours	0.63	CPC 3-5 at 6 months	221	0.74	Sensitivity 84.9% (78.2-90.2), FPR 0.16 (0.08- [illegible]	Moderate	Study confounding moderate
Velly 2018	29500154	Corneal reflex absent, bilateral	On admission	0.18	CPC 3-5 at 6 months	150	0.78	FPR 0.09	High	Study participation high, Prognostic factor measurement high, Self-fulfilling prophecy high
Wu 2011	21330629	Corneal reflex absent, bilateral	72h from ROSC	0.43	mRS>4 at 6 months	151	0.91	Sensitivity 50% (39–61), Specificity 100% (73–100), significant in multivariate analysis	High	Study participation moderate, Self-fulfilling prophecy high

Pupillary light response and Corneal reflex as predictors of mortality

FIRST AUTHOR LAST NAME	PMID	PROGNOSTIC FACTOR(S) EVALUATED	Category of Predictor	Timing of prognostic factor evaluation	Prevalence % of the predictor	OUTCOME(S)	Sample size for the outcome	Prevalence % of the outcome	EFFECT SIZE WITH 95% CI as reported in the study (False Positive Rate, Sensitivity, Specificity, Odds Ratio, Relative Risk)	OVERALL RISK OF BIAS FOR THE STUDY	Overall risk of bias: Comments
Rossetti 2010	20373341	Either pupillary, corneal or oculocephalic reflexes absent bilaterally	36-72 hours from ROSC	Mortality in-hospital, all-cause	0.42	Mortality in-hospital, all-cause	109	0.59	FPR 0.04	High	Self fulfilling prophecy high
Youn 2015	26164682	Four B 3 or 4- at least one pupillary or corneal reflex present	Pupillary reflex	<6h from ROSC	0.31	Survival in-house, all-cause	331	0.29	OR 10.21 (95% CI 4.50 to 23.16)	High	Study participation moderate, Self-fulfilling prophecy high

Pupillary light response and Corneal reflex as predictors of mortality

Limpawattana 2018	29593417	Pupillary light response absent	Pupillary reflex	Not stated	0.35	Mortality 1 year	202	0.92	Adjusted hazard ratio 2.529 (95%CI 1.378 - 4.642), p=0.003 in multivariate best-fit model	Moderate	Self fulfilling prophecy moderate

Motor response as a predictor of functional outcome

FIRST AUTHOR LAST NAME	PMID	PROGNOSTIC FACTOR(S) EVALUATED	Timing of prognostic factor evaluation	Prevalence % of the predictor	OUTCOME(S)	Sample size for the outcome	Prevalence % of the outcome	EFFECT SIZE WITH 95% CI as reported in the study (False Positive Rate, Sensitivity, Specificity, Odds Ratio, Relative Risk)	OVERALL RISK OF BIAS FOR THE STUDY	Overall risk of bias: Comments
Levy 1985	3968772	Motor response flexion or worse	Day 3	0.33	Poor functional outcome- Severe disability (dependence for some functions) or worse	210	0.88	FPR 0% (0-7%)	High	Study confounding moderate, Self fulfilling prophecy high
Edgren 1994	7909098	Motor response absent	72h from ROSC	0.16	CPC 3-5 at 6-12 months	262	0.54	FPR 0	High	Study confounding moderate, Statistical analysis moderate, Self fulfilling prophecy high
Rossetti 2010	2E+07	GCS M 1 or 2	36-72 hours from ROSC	0.62	CPC 3-5 at 3-6 months	109	0.77	FPR 0.16	High	Self fulfilling prophecy high

Motor response as a predictor of functional outcome

Wu 2011	2.1E+07	GCS M 1 or 2	72h from ROSC	0.72	mRS>4 at 6 months	151	0.91	Sensitivity 82% (72–89), Specificity 79% (49–94), significant in multivariate analysis	High	Study participation moderate, Self-fulfilling prophecy high
Greer 2013	2.4E+07	GCS motor response extensor or absent	Day 3, Day 7	Day 3- 0.65, Day 7- 0.49	mRS 4-6 at 6 months	200	0.90	Day 3- FPR 0.3, Sensitivity 0.81. Day 7- FPR 0.05, Sensitivity 0.74.	High	Study confounding moderate, Self-fulfilling prophecy high
Dragancea 2015	2.6E+07	GCS M 1 or 2	Median 117 (93-137) hours	0.73	CPC 3-5 at 3-6 months	312	0.85	FPR 0.19 (0.10–0.33), Sensitivity 82.3% (77.2–86.4)	Moderate	Study participation moderate, Study confounding moderate, Self-fulfilling prophecy moderate. TTM trial substudy.

Motor response as a predictor of functional outcome

Hifumi 2015	2.6E+07	GCS-M4-5	Immediately after admission, without sedation or paralytics.	0.08	CPC 1-2 at 90 days	302	0.57	OR 8.18 (1.9-60.28), p<0.01. Sensitivity 12% (7 - 17), Specificity 98% (93 - 99).	High	Prognostic factor measurement high
Velly 2018	3E+07	GCS M 1 or 2	Day 7	0.57	CPC 3-5 at 6 months	150	0.78	FPR 0.15, OR 12·60 (4·85–39·49), p<0·0001	High	Study participation high, Prognostic factor measurement high, Self-fulfilling prophecy high
Ebner 2019	3.1E+07	GCS-M=2-5 vs GCS-M=1	Median 117 hours	0.49	CPC 3-5 at 6 months	869	0.49	OR 0.4 (0.28-0.57), p<0.001	Moderate	Study participation moderate, Self-fulfilling prophecy moderate. TTM trial substudy.
Nakstad 2020	3.2E+07	GCS M 1-3	>72h	0.49	CPC 3-5 at 6 months	185	0.51	FPR 0.27 (0.18-0.37)	High	Study confounding moderate, Self fulfilling prophecy high

Motor response as a predictor of functional outcome

Nobile 2020	3.2E+07	Motor response-absent or posturing at 72h	72h from ROSC	0.53	CPC 3-5 at 3 months	532	0.63	AUC 0.77 (95% CI 0.72 to 0.81), p<0.001	High	Study participation high, Self-fulfilling prophecy High

Motor response as a predictor of mortality

FIRST AUTHOR LAST NAME	PMID	PROGNOSTIC FACTOR(S) EVALUATED	Timing of prognostic factor evaluation	Prevalence % of the predictor	OUTCOME(S)	Sample size for the outcome	Prevalence % of the outcome	EFFECT SIZE WITH 95% CI as reported in the study (False Positive Rate, Sensitivity, Specificity, Odds Ratio, Relative Risk)	OVERALL RISK OF BIAS FOR THE STUDY	Overall risk of bias: Comments
Grubb 1995	7623574	GCS<6	72h from ROSC	0.10	Mortality in-hospital, all-cause	270	0.51	FPR 0%	High	Self fulfilling prophecy high
Rossetti 2010	20373341	GCS M 1 or 2	36-72 hours from ROSC	0.62	Mortality in-hospital, all-cause	109	0.59	FPR 24%	High	Self fulfilling prophecy high

Early myoclonus as a predictor of functional outcome

FIRST AUTHOR LAST NAME	PMID	PROGNOSTIC FACTOR(S) EVALUATED	Timing of prognostic factor evaluation	Prevalence % of the predictor	OUTCOME(S)	Sample size for the outcome	Prevalence % of the outcome	EFFECT SIZE WITH 95% CI as reported in the study (False Positive Rate, Sensitivity, Specificity, Odds Ratio, Relative Risk)	OVERALL RISK OF BIAS FOR THE STUDY	Overall risk of bias: Comments
Greer 2013	23954666	Status myoclonus	First 24 hours	0.31	mRS 4-6 at 6 months	200	0.90	FPR 0.10, Sensitivity- 0.33	High	Prognostic factor measurement high, Study confounding moderate, Self-fulfilling prophecy high

Early myoclonus as a predictor of functional outcome

Rossetti 2010	20373341	Status myoclonus	Within 24 hours of weaning sedation. Therapeutic hypothermia was used for all patients for 24 hours. Sedation was then stopped after passive rewarming to a central temperature of 35°C.	0.33	CPC 3-5 at 3-6 months	109	0.77	FPR 0.04	High	Prognostic factor measurement high, Self fulfilling prophecy high
Velly 2018	29500154	Status myoclonus	Not stated	0.14	CPC 3-5 at 6 months	150	0.78	FPR 0, OR $3{\cdot}98 \times 10^7$ (0·00–NA), p=0·9902	High	Study participation high, Prognostic factor measurement high, Self-fulfilling prophecy high

Early myoclonus as a predictor of mortality

FIRST AUTHOR LAST NAME	PMID	PROGNOSTIC FACTOR(S) EVALUATED	Timing of prognostic factor evaluation	Prevalence % of the predictor	OUTCOME(S)	Sample size for the outcome	Prevalence % of the outcome	EFFECT SIZE WITH 95% CI as reported in the study (False Positive Rate, Sensitivity, Specificity, Odds Ratio, Relative Risk)	OVERALL RISK OF BIAS FOR THE STUDY	Overall risk of bias: Comments
Rossetti 2010	20373341	Status myoclonus	Within 24 hours of weaning sedation. Therapeutic hypothermia was used for all patients for 24 hours. Sedation was then stopped after passive rewarming to a central temperature of 35°C.	0.33	Mortality in-hospital, all-cause	111	0.59	FPR 0.04	High	Prognostic factor measurement high, Self fulfilling prophecy high

CT diffuse loss of grey white differentiation and sulcal effacement as a predictor of functional outcome

FIRST AUTHOR LAST NAME	PMID	PROGNOSTIC FACTOR(S) EVALUATED	Timing of prognostic factor evaluation	Prevalence % of the predictor	OUTCOME(S)	Sample size for the outcome	Prevalence % of the outcome	EFFECT SIZE WITH 95% CI as reported in the study (False Positive Rate, Sensitivity, Specificity, Odds Ratio, Relative Risk)	OVERALL RISK OF BIAS FOR THE STUDY	Overall risk of bias: Comments
Wu 2011	21330629	CT Hounsfeld units whole brain	<=72h from ROSC	Not stated, threshold not stated	mRS>4 at 6 months	151	0.91	Sensitivity 1% (0–7), Specificity 100% (73–100), significant in multivariate analysis	High	Study participation moderate, Self-fulfilling prophecy high
Wu 2011	21330629	CT Hounsfeld units whole putamen + posterior limb internal capsule	<=72h from ROSC	Not stated, threshold not stated	mRS>4 at 6 months	151	0.91	Sensitivity 25% (15–33), Specificity 100% (73–100), significant in multivariate analysis	High	Study participation moderate, Self-fulfilling prophecy high

CT diffuse loss of grey white differentiation and sulcal effacement as a predictor of functional outcome

Wu 2011	21330629	CT Hounsfeld units whole putamen/ posterior limb internal capsule	<=72h from ROSC	Not stated, threshold not stated	mRS>4 at 6 months	151	0.91	Sensitivity 8% (4–16), Specificity 100% (73–100), significant in multivariate analysis	High	Study participation moderate, Self-fulfilling prophecy high
Hong 2019	30953628	CT- Grey-white ratio	Within 2 hours of ROSC	Continuous	CPC 3-5 at 6 months	512	0.68	Multivariate analysis: GWRaverage p=0.727, GWRbasal ganglia	High	Prognostic factor measurement high
Moseby-Knappe 2018	30383090	CT Gen edema	Median 23 (2-19) hours	0.25	CPC 3-5 at 6 months	261	0.65	Sensitivity 37%, Specificity 97%	High	Study participation moderate, Prognostic factor measurement high, Study confounding moderate, Self-fulfilling prophecy moderate. TTM trial substudy

CT diffuse loss of grey white differentiation and sulcal effacement as a predictor of mortality

FIRST AUTHOR LAST NAME	PMID	PROGNOSTIC FACTOR(S) EVALUATED	Timing of prognostic factor evaluation	Prevalence % of the predictor	OUTCOME(S)	Sample size for the outcome	Prevalence % of the outcome	EFFECT SIZE WITH 95% CI as reported in the study (False Positive Rate, Sensitivity, Specificity, Odds Ratio, Relative Risk)	OVERALL RISK OF BIAS FOR THE STUDY	Overall risk of bias: Comments
Chang 2019	31521958	CT TENS score	Prior to TTM	TENS >0 in 0.38, TENS >1 in 0.20	Mortality in-hospital, all-cause	341	0.64	p<0.05 for TENS >0 and p<0.01 for TENS >1	High	Study confounding high, Statistical analysis high, Self fulfilling prophecy high
Fisher 2020	33021889	CT- cerebral edema	In emergency department	0.16	Mortality in-hospital, all-cause	254	0.30	Adjusted OR 13.7, 95% CI 3.26–57.4	High	Prognostic factor measurement high, Self fulfilling prophecy high

MRI diffuse restricted diffusion as a predictor of functional outcome

FIRST AUTHOR LAST NAME	PMID	PROGNOSTIC FACTOR(S) EVALUATED	Timing of prognostic factor evaluation	Prevalence % of the predictor	OUTCOME(S)	Sample size for the outcome	Prevalence % of the outcome	EFFECT SIZE WITH 95% CI as reported in the study (False Positive Rate, Sensitivity, Specificity, Odds Ratio, Relative Risk)	OVERALL RISK OF BIAS FOR THE STUDY	Overall risk of bias: Comments	Other comments
Oh 2019	31215475	MRI DWI No lesion	After Normothermia,>38-48h from ROSC	0.23	CPC 1-2 at 6 months	134	0.27	Sensitivity 72.2% (95%CI 54.8–85.8), Speificity 94.9% (95%CI 88.5–98.3). AUC 0.94 (95%CI, 0.88–0.97). AUC 0.94 (95%CI 0.88–0.97)	Moderate	Study participation moderate, Study confounding moderate	
Oh 2019	31215475	MRI DWI No lesion or isolated cortex or deep gray matter lesion	After Normothermia,>38-48h from ROSC	0.31	CPC 1-2 at 6 months	134	0.27	Sensitivity 94.4% (95%CI 81.3–99.3), Speificity 91.8% (95%CI 84.6–96.4). AUC 0.94 (95%CI, 0.88–0.97)	Moderate	Study participation moderate, Study confounding moderate	
Oh 2019	31215475	MRI DWI multifocal or global lesions	After Normothermia,>38-48h from ROSC	0.69	CPC 3-5 at 6 months	134	0.73	Sensitivity 91.8% (95%CI 84.6–96.4), Speificity 94.4% (95%CI 81.3–99.3)	Moderate	Study participation moderate, Study confounding moderate	

MRI diffuse restricted diffusion as a predictor of functional outcome

Velly 2018	29500154	MRI FLAIR-DWI intensity presence in cortex and deep grey matter, and FLAIR-DWI scoring system. Also DTI.	Day 7-28	FLAIR- DWI hyperintensity in deep grey nuclei 0.66, in cortex 0.45.	CPC 3-5 at 6 months	148	0.78	FLAIR-DWI OVERALL SCORE: AUC 0·83 (0·76–0·90), For cutoff ≥41, Specificity 100% (89–100), Sensitivity 40% (31–50), PPV 100% (92–100), NPV 32% (23–42). FLAIR-DWI CORTEX SCORE: AUC 0·75 (0.67–0·84), For cutoff ≥30, Specificity 100% (89–100), Sensitivity 33% (25–42), PPV 100% (91–100), NPV 30% (22–39). FLAIR-DWI CORTEX PLUS DEEP GREY NUCLEI SCORE: AUC 0·81 (0·74–0·88), For cutoff ≥41, Specificity 100% (89–100), Sensitivity 37% (28–46), PPV 100% (92–100), NPV [illegible]	High	Study participation high, Self-fulfilling prophecy high	MRI FLAIR-DWI score: The adjudicators were instructed to only score MRI abnormalities that could be attributed to acute global hypoxic-ischemic brain injury. The brain regions were: cortical gray matter and subcortical white matter in the frontal, parietal, temporal, and occipital [illegible]

MRI diffuse restricted diffusion as a predictor of functional outcome

Moon 2018	29474879	MRI apparent diffusion coefficient (ADC) value- average across all voxels.	Within 7 days of ROSC. Early (within 48 h after return of spontaneous circulation (ROSC)) and late group (between 48 h and 7 days after ROSC) according to the DWI scan time	Continuous. No further data on prevalence of specific thresholds	CPC 3-5 at 6 months	96 patients, 110 scans. Early 44, Late 66.	0.68	Mean ADC was not an indepndent predictor in the logistic regression analysis in the early group, but was in the late group (OR 0.98, 95% CI 0.97–0.99). In the late group AUC 0.82 (0.71-0.91), sensitivity 21% (11-35) and specificity 100% (82-100).	Moderate	Study participation moderate	The standard of b=1000 s/mm2 was used for all DWIs. Voxels with ADC values under 50^−6mm2/s or above 1200^−6mm 2/s were extracted from the analysis to exclude artifacts or cerebrospinal fluid.

MRI diffuse restricted diffusion as a predictor of functional outcome

Moon 2018	29474879	MRI apparent diffusion coefficient (ADC) value measured in all voxels. Percentage of voxels below the specified apparent ADC value was the predictor. The ADC thresholds ranged from 400 to 750×10^−6 mm2/s and were analyzed at 50×10^−6mm2/s intervals.	Within 7 days of ROSC. Early (within 48 h after return of spontaneous circulation (ROSC)) and late group (between 48 h and 7 days after ROSC) according to the DWI scan time	Continuous. No further data on prevalence of specific thresholds	CPC 3-5 at 6 months	96 patients, 110 scans. Early 44, Late 66.	0.68	ADC threshold of 400 × 10^-6 mm2/s- percentage voxels below this threshold of 2.5% had AUC 0.90 (0.84-0.95), Sensitivity 67% (55–78), Specificity 100% (91–100). In the early group, AUC was 0.89 (0.79-0.99), sensitivity 64% (43–82) and specificity 100% (82-100). Thresholds of 400-600 achieved statistical significance in the logistic regression analysis in the early group, while all thresholds (400-750) achieved statistical significance in logistic regression analysis in the late	Moderate	Study participation moderate	The standard of b=1000 s/mm2 was used for all DWIs. Voxels with ADC values under 50^−6mm2/s or above 1200^−6mm2/s were extracted from the analysis to exclude artifacts or cerebrospinal fluid.

MRI diffuse restricted diffusion as a predictor of functional outcome

Hirsch 2020	32269116	>10% of brain tissue with an ADC <650 ×10^-6 mm2/s	Within 7 days of cardiac arrest	Not clearly stated	Glasgow Outcome Scale score of ≤2 at 6 months after arrest	97 (100 enrolled)	0.57	Sensitivity of 0.63 (95% confidence interval [CI] 0.42-0.80), a specificity of 0.96 (95% CI 0.77-0.998), and a positive predictive value (PPV) of 0.94 (95% CI 0.71-0.997)	Moderate	Study participation moderate, Study confounding moderate	Other predictors such as pupillar reactivity, GCS M1-2 and SSEP studies, but N for these seems to be well below 100.

MRI diffuse restricted diffusion as a predictor of mortality

FIRST AUTHOR LAST NAME	PMID	PROGNOSTIC FACTOR(S) EVALUATED	Timing of prognostic factor evaluation	Prevalence % of the predictor	OUTCOME(S)	Sample size for the outcome	Prevalence % of the outcome	EFFECT SIZE WITH 95% CI as reported in the study (False Positive Rate, Sensitivity, Specificity, Odds Ratio, Relative Risk)	OVERALL RISK OF BIAS FOR THE STUDY	Overall risk of bias: Comments
No studies met criteria.										

EEG suppression or burst suppression as a predictor of functional outcome

FIRST AUTHOR LAST NAME	PMID	PROGNOSTIC FACTOR(S) EVALUATED	Timing of prognostic factor evaluation	Prevalence % of the predictor	OUTCOME(S)	Sample size for the outcome	Prevalence % of the outcome	EFFECT SIZE WITH 95% CI as reported in the study (False Positive Rate, Sensitivity, Specificity, Odds Ratio, Relative Risk)	OVERALL RISK OF BIAS FOR THE STUDY	Overall risk of bias: Comments
Nakstad 2020	31926258	EEG grade 4-5. Synek EEG Grade 4- burst suppression, generalized epileptic activity including status myoclonus, nonreactive with low voltage, alpha coma, theta coma. Grade 5- No visible activity on high sensitivity registration	>72h	0.29	CPC 3-5 at 6 months	107	0.51	FPR 0.05 (0.01-0.15)	Moderate	Prognostic factor measurement moderate, Study confounding moderate, Self fulfilling prophecy high
Ruitjer 2019	31155751	EEG- Unfavorable. Unfavorable EEG- Generalized EEG suppression (all activity <10 µV) and synchronous patterns with ≥50% suppression	6-120 hours	0.24	CPC 3-5 at 6 months	469	0.25	Beta coefficient 5.922 (SE 1.40), p<0.001. FPR 0% for suppression, synchronous burst suppression and and GPD.	High	Study participation moderate, Self-fulfilling prophecy high

EEG suppression or burst suppression as a predictor of functional outcome

Ruitjer 2019	31155751	EEG- Favorable. Favorable EEG- Continuous EEG pattern	6-120 hours	0.29	CPC 1-2 at 6 months	469	0.75	Beta coefficient 2.531 (SE 0.314), p<0.001	High	Study participation moderate, Self-fulfilling prophecy high
Admiraal 2020	32651293	EEG reactivity	12-24 hours from ROSC	0.4 of EEG recordings	CPC 3-5 at 6 months	108	0.48	Prognostic accuracy of EEG reactivity for GOOD outcome- Sensitivity 75% (62.1–85.3), Specificity 65.2% (42.7–83.6), Positive predictive value 84.9% (72.4–93.3), Negative predictive value 50% (31.3–68.7). Patients with a continuous (n = 64) or discontinuous (n = 19) normal voltage background pattern with reactivity were 3 and 8 times more likely to have a good outcome than without reactivity (continuous: Diagnostic Odds Ratio DOR, 3.4; 95% confidence interval [CI], 0.97–12.0; p = 0.06; discontinuous: DOR, 8.0; 95% CI, 1.0–63.97; p =	Moderate	Study confounding moderate, Statistical analysis moderate

EEG suppression or burst suppression as a predictor of functional outcome

Westhall 2018	29933239	EEG- Absence of continuous activity within 24 hours	12-60 hours from ROSC	0.46	CPC 3-5 at 6 months	134	0.51	OR 16.8 (95%CI 7.1–40.1), p<0.001. PPV 0.84 (0.72–0.92), NPV 0.76 (0.65–0.85), Sensitivity 0.75 (0.63–0.85), Specificity 0.85 (0.73–0.92)	Moderate	Study participation moderate, Study confounding moderate
Westhall 2018	29933239	EEG- Absence of continuous activity within 60 hours	12-60 hours from ROSC	0.23	CPC 3-5 at 6 months	134	0.51	OR Infinite. PPV 1.00 (0.86–1.00), NPV 0.63 (0.53–0.72), Sensitivity 0.45 (0.33–0.57), Specificity 1.00 (0.93–1.00)	Moderate	Study participation moderate, Study confounding moderate
Westhall 2018	29933239	EEG- epileptiform activity within 24 hours	12-60 hours from ROSC	0.28	CPC 3-5 at 6 months	134	0.51	OR 14.8 (95%CI 4.9–45.2), p<0.001. PPV 0.89 (0.74–0.97), NPV 0.63 (0.53–0.73), Sensitivity 0.49 (0.37–0.61), Specificity 0.94 (0.84–0.98)	Moderate	Study participation moderate, Study confounding moderate

EEG suppression or burst suppression as a predictor of functional outcome

Westhall 2016	26865516	EEG highly malignant pattern	12–36 hours after rewarming in patients who were still comatose, typically corresponding to 48–72 hours after the cardiac arrest or later if this period coincided with a	0.37	CPC 3-5 at 6 months	103	0.74	Sensitivity 50% (39–61), Specificity 100% (88–100)	Moderate	
Moseby-Knappe 2018	30383090	EEG highly malignant pattern	Median 67 (51-97) hrs	0.37	CPC 3-5 at 6 months	81	0.78	Sensitivity 48%, Specificity 100%	Moderate	Study participation moderate, Study confounding moderate, Self-fulfilling prophecy moderate. TTM trial substudy.

EEG suppression or burst suppression as a predictor of functional outcome

Velly 2018	29500154	EEG synek classification grade 4-5	Median 3 days (IQR 2-5)	0.26	CPC 3-5 at 6 months	136	0.80	FPR for grades 4-5 was 0.04. For Grade 5- Specificity 100% (87–100), Sensitivity 5% (2–10), PPV 100% (48–100), NPV 21% (14–29). For overall Synek classification OR 3·80 (2·01–7·95), p=0·0001.	High	Study participation high, Prognostic factor measurement moderate, Self-fulfilling prophecy high
Velly 2018	29500154	EEG nonreactive	Median 3 days (IQR 2-5)	0.70	CPC 3-5 at 6 months	136	0.80	FPR 0.44.	High	Study participation high, Prognostic factor measurement moderate, Self-fulfilling prophecy high
Rossetti 2010	20373341	EEG- epileptiform activity on routine EEG	36-72 hours from ROSC. Median 2 days.	0.36	CPC 3-5 at 3-6 months	108	0.77	FPR 0.12	High	Prognostic factor measurement moderate, Self-fulfilling prophecy high
Rossetti 2010	20373341	EEG- nonreactive background on routine EEG	36-72 hours from ROSC. Median 2 days.	0.52	CPC 3-5 at 3-6 months	108	0.77	FPR 0	High	Prognostic factor measurement moderate, Self-fulfilling prophecy high

EEG suppression or burst suppression as a predictor of functional outcome

Glimmerveen 2020	32425878	Suppressed, or synchronous patterns on suppressed background	12 hours from ROSC	0.4	CPC 3-5 at 6 months	97	0.74	Specificty 100% (87–100), Sensitivity 37% (28–47), PPV 100% (89–100), NPV 32% (24–43)	Moderate	Study confounding moderate, Self-fulfilling prophecy moderate
Glimmerveen 2020	32425878	Suppressed, or synchronous patterns on suppressed background	24 hours from ROSC	0.26	CPC 3-5 at 6 months	123	0.76	Specificty 100% (87–100), Sensitivity 30% (22–40), PPV 100% (89–100), NPV 32% (24–43)	Moderate	Study confounding moderate, Self-fulfilling prophecy moderate
Glimmerveen 2020	32425878	Continuous EEG at 12 hours	12 hours from ROSC	0.09	CPC 1-2 at 6 months	89	0.28	Specificty 98% (92–100), Sensitivity 19% (8–37), PPV 75% (36–96), NPV 80% (72–86)	Moderate	Study confounding moderate, Self-fulfilling prophecy moderate
Scarpino 2021	33819501	Suppression or burst suppression on EEG at 72h	72h from ROSC	0.24	CPC 3-5 at 6 months	403	0.66	FPR 4% (0-3%), sensitivity 64% (56-71)	Moderate	Study participation moderate, Study confounding moderate

EEG suppression or burst suppression as a predictor of mortality

FIRST AUTHOR LAST NAME	PMID	PROGNOSTIC FACTOR(S) EVALUATED	Timing of prognostic factor evaluation	Prevalence % of the predictor	OUTCOME(S)	Sample size for the outcome	Prevalence % of the outcome	EFFECT SIZE WITH 95% CI as reported in the study (False Positive Rate, Sensitivity, Specificity, Odds Ratio, Relative Risk)	OVERALL RISK OF BIAS FOR THE STUDY	Overall risk of bias: Comments
Youn 2015	26164682	NON-malignant EEG. Malignant EEG defined as- non-convulsive status epilep-ticus (NCSE), convulsive status epilepticus (CSE), myoclonic statusepilepticus (MSE) and generalized periodic epileptiform discharges (GPEDs).	<48h from ROSC	0.69	Survival in-house, all-cause	331	0.29	OR 13.00 (95% CI 5.09 to 33.18)	High	Study participation moderate, Prognostic factor measurement moderate, Self-fulfilling prophecy high

EEG suppression or burst suppression as a predictor of mortality

Faro 2019	30586605	EEG patterns	Within 5 days of ROSC	Never checked 0.38, Nothing malignant 0.31, Epileptiform discharges 0.04, Periodic discharges 0.05, Seizures 0.02, Polyspike 0.21	Survival in-hospital, all-cause	818	0.28	Survival: Never checked 0.38 (0.32 – 0.43), Nothing malignant 0.29 (0.24 – 0.35), Epileptiform discharges 0.32 (0.17 – 0.50), Periodic discharges 0.27 (0.14 – 0.43), Seizures 0.17 (0.02 – 0.48), Polyspike 0.07 (0.04 – 0.12)	High	Study participation moderate, Prognostic factor measurement moderate, Study confounding moderate, Statistical analysis modrate, Self fulfilling prophecy [illegible]

EEG suppression or burst suppression as a predictor of mortality

Amorim 2016	27554945	EEG- status epilepticus, Nonreactive or Pure suppression burst. EEG suppression burst per ACNS, pure SB if no other features present. EEG status epileptoicus per NCS SE version 1 guidelines. EEG reactivity was best obtained in first 72 hours, tested every day, defined as change in EEG background frequency or amplitude after a noxious or auditory stimulus	EEG start at TTM, at least 10 hours. EEG background reactivity- best within 72 hours.	EEG status epilepticus 0.31, GPD 0.08, discrete seizure 0.005, Pure suppression burst 0.23, Nonreactive 0.71	Mortality in-hospital, all-cause	373	0.69	STATUS EPILEPTICUS: Sensitivity 42% (36–49), Specificity 92% (85–96), PPV 92% (86–96), NPV 42% (36–49), FPR 0.08 (0.04–0.15). NONREACTIVE EEG BACKGROUND: Sensitivity 96% (93–98), Specificity 83% (75–89), PPV 92% (88–95), NPV 91% (83–95), FPR 0.17 (0.11–0.25). PURE SUPPRESSION BURST: Sensitivity 25% (2–31), Specificity 81% (73–88), PPV 75% (64–83), PPV 33% (28–39), FPR 0.19 (0.12–0.27)	High	Self fulfilling prophecy high
Rossetti 2010	20373341	EEG- epileptiform activity on routine EEG	36-72 hours from ROSC. Median 2 days.	0.35	Mortality in-hospital, all-cause	110	0.59	FPR 0.09	High	Prognostic factor measurement high, Self-fulfilling prophecy high
Rossetti 2010	20373341	EEG- epileptiform activity on routine EEG	36-72 hours from ROSC. Median 2 days.	0.51	Mortality in-hospital, all-cause	110	0.59	FPR 0.07	High	Prognostic factor measurement high, Self-fulfilling prophecy high

EEG suppression or burst suppression as a predictor of mortality

Rossetti 2007	17636063	EEG status epilepticus. One EEG certified author (A.O.R.) reviewed retrospectively on two parallel computerized databases all reports and selected tracings. SE was defined by prolonged (>5 minutes) spontaneous or stimulus-induced occurrence of repetitive or rhythmic focal or generalized spikes, sharp waves, spike and waves, or rhythmic waves evolving in amplitude, frequency, or field. Spontaneous burst-suppression with epileptiform bursts and PEDs were [illegible]	Median 2 days (10% to 90%: 1 to 4)	0.33	Mortality in-hospital, all-cause	107	0.66	FPR 0.38. Multivariate analysis goodness of fit was valid only in patients treated with hypothermia, N=63- for status epilepticus, OR was 14 (3-75).	High	Prognostic factor measurement high, Self fulfilling prophecy high

Somatosensory evoked potentials (SSEP) as a predictor of functional outcome

FIRST AUTHOR LAST NAME	PMID	PROGNOSTIC FACTOR(S) EVALUATED	Timing of prognostic factor evaluation	Prevalence % of the predictor	OUTCOME(S)	Sample size for the outcome	Prevalence % of the outcome	EFFECT SIZE WITH 95% CI as reported in the study (False Positive Rate, Sensitivity, Specificity, Odds Ratio, Relative Risk)	OVERALL RISK OF BIAS FOR THE STUDY	Overall risk of bias: Comments
Nakstad 2020	31926258	SSEP bilateral N20 absent	>72h	0.16	CPC 3-5 at 6 months	50	0.51	FPR 0 (0)	Moderate	Study confounding moderate, Self fulfilling prophecy moderate
Ruitjer 2019	31155751	SSEP bilateral N20 absent	Not stated	0.39	CPC 3-5 at 6 months	319	0.87	FPR 0%	High	Study participation moderate, Self-fulfilling prophecy high
Nobile 2020	32114066	SSEP bilateral N20 absent	After Normothermia, 48-72h from ROSC	0.27	CPC 3-5 at 3 months	532	0.63	Sensitivity 43%, Positive predictive value 100%	High	Study participation high, Self-fulfilling prophecy High

Somatosensory evoked potentials (SSEP) as a predictor of functional outcome

Oh 2020	32169609	SSEP bilateral N20 absent	75 h (IQR, 62-96) for patients with a good outcome and 70 h (IQR, 58-91) for patients with a poor outcome (p = 0.233)	0.48	CPC 3-5 at 6 months	262	0.68	Sensitivity 71.0% (95% CI, 63.7-77.5) and an FPR 0% (95% CI, 0.0-4.3)	Moderate	Study confounding moderate. In South Korea, withdrawal of life support is not permitted. Study excluded 8 patients who underwent WLST. 31 patients with absent N20 (vs 7 with N20s present) were permitted a DNR order and died before hospital discharge e. Limitations of active treatment and DNR orders statistically significantly higher in patients without pupillary light reflex as also corneal reflex.

Somatosensory evoked potentials (SSEP) as a predictor of functional outcome

Oh 2019	31215475	SSEP bilateral N20 absent	After Normothermia ,>38-48h from ROSC	0.22	CPC 3-5 at 6 months	192	0.73	Sensitivity 30.5% (95%CI 23.0–38.8), Specificity 100% (95% CI 93.0–100.0). AUC 0.65 (95% CI 0.58–0.72)	Moderate	Study participation moderate, Study confounding moderate
Oh 2019	31215475	SSEP bilateral P25 absent	After Normothermia ,>38-48h from ROSC	0.52	CPC 3-5 at 6 months	192	0.73	Sensitivity 70.2% (95% CI 61.9–77.6), Specificity 100% (95% CI 93.0–100.0), AUC 0.85 (95% CI 0.79–0.90)	Moderate	Study participation moderate, Study confounding moderate
Oh 2019	31215475	SSEP bilateral N20 OR P25 absent	After Normothermia ,>38-48h from ROSC	0.53	CPC 3-5 at 6 months	192	0.73	Sensitivity 71.6% (95% CI 63.4–78.9), Specificity 100% (95% CI 93.0–100.0). AUC 0.86 (95% CI, 0.80–0.90)	Moderate	Study participation moderate, Study confounding moderate

Somatosensory evoked potentials (SSEP) as a predictor of functional outcome

Oh 2019	31215475	N20-P25 amplitude <0.64mcV	After Normothermia ,>38-48h from ROSC	0.55	CPC 3-5 at 6 months	192	0.73	Sensitivity 74.5% (95% CI 66.5–81.4), Specificity 100% (95% CI 93.0–100.0). AUC of N20-P25 amplitude was 0.94 (95% CI, 0.90–0.97)	Moderate	Study participation moderate, Study confounding moderate
Oh 2019	31215475	N20-P25 amplitude >5.04mcV	After Normothermia ,>38-48h from ROSC	0.03	CPC 1-2 at 6 months	192	0.73	Sensitivity 9.8% (95%CI 3.3–21.4), Specificity 100% (95% CI 97.4–100.0). AUC of N20-P25 amplitude 0.94 (95% CI, 0.90–0.97)	Moderate	Study participation moderate, Study confounding moderate
Moseby-Knappe 2018	30383090	SSEPs bil absent N20	Median 93 hrs	0.38	CPC 3-5 at 6 months	170	0.81	Sensitivity 47%, Specificity 97%	Moderate	Study participation moderate, Study confounding moderate, Self-fulfilling prophecy moderate. TTM trial substudy.

Somatosensory evoked potentials (SSEP) as a predictor of functional outcome

Rossetti 2010	20373341	SSEP bilateral N20 absent	Median 2.0-2.5 days from ROSC. At least 24 hours after weaning sedation. Therapeutic hypothermia was used for all patients for 24 hours. Sedation was then stopped after passive rewarming to a central temperature of 35°C.	0.35	CPC 3-5 at 3-6 months	95	0.75	FPR 0	High	Study participation moderate, Self fulfilling prophecy high
Glimmerveen 2020	32425878	SSEP bilateral N20 absent	48-72h from ROSC	0.3	CPC 3-5 at 6 months	138	0.77	Sensitivity 39%, Specificity 100%	Moderate	Study confounding moderate, Self-fulfilling prophecy moderate
Scarpino 2021	33819501	SSEP bilateral N20 absent	72h from ROSC	0.22	CPC 3-5 at 6 months	403	0.66	FPR 0% (0-5%), sensitivity 53% (45-61).	Moderate	Study participation moderate, Study confounding moderate. No withdrawal of life support in Italy

Somatosensory evoked potentials (SSEP) as a predictor of mortality

FIRST AUTHOR LAST NAME	PMID	PROGNOSTIC FACTOR(S) EVALUATED	Timing of prognostic factor evaluation	Prevalence % of the predictor	OUTCOME(S)	Sample size for the outcome	Prevalence % of the outcome	EFFECT SIZE WITH 95% CI as reported in the study (False Positive Rate, Sensitivity, Specificity, Odds Ratio, Relative Risk)	OVERALL RISK OF BIAS FOR THE STUDY	Overall risk of bias: Comments
Rossetti 2010	20373341	SSEP bilateral N20 absent	Median 2.0-2.5 days from ROSC. At least 24 hours after weaning sedation. Therapeutic hypothermia was used for all patients for 24 hours. Sedation was then stopped after passive rewarming to a central temperature of 35°C.	0.33	Mortality in-hospital, all-cause	100	0.56	FPR 0	High	Study participation moderate, Self fulfilling prophecy high

Neuron Specific Enolase (NSE) as a predictor of functional outcome

FIRST AUTHOR LAST NAME	PMID	PROGNOSTIC FACTOR(S) EVALUATED	Timing of prognostic factor evaluation	Prevalence % of the predictor	OUTCOME	Sample size for the outcome	Prevalence % of the outcome	EFFECT SIZE WITH 95% CI as reported in the study (False Positive Rate, Sensitivity, Specificity, Odds Ratio, Relative Risk)	OVERALL RISK OF BIAS FOR THE STUDY	Overall risk of bias: Comments
Nakstad 2020	31926258	NSE highest value > 33 mg/L	24h and 48h	Not clearly stated	CPC 3-5 at 6 months	258	0.51	FPR 0.34 (0.26-0.43)	Moderate	Study confounding moderate
Nakstad 2020	31926258	NSE highest value > 60 mg/L	24h and 48h	Not clearly stated	CPC 3-5 at 6 months	258	0.51	FPR 0.07 (0.03-0.13)	Moderate	Study confounding moderate
Nakstad 2020	31926258	NSE highest value > 60 mg/L	24h and 48h	Not clearly stated	CPC 3-5 at 6 months	258	0.51	FPR 0.07 (0.03-0.13)	Moderate	Study confounding moderate
Nakstad 2020	31926258	NSE highest value > 80 mg/L	24h and 48h	Not clearly stated	CPC 3-5 at 6 months	258	0.51	FPR 0.02 (0.01-0.07)	Moderate	Study confounding moderate
Nakstad 2020	31926258	NSE increases after 24 h	24h and 48h	Not clearly stated	CPC 3-5 at 6 months	225	0.51	FPR 0.32 (0.24-0.41)	Moderate	Study confounding moderate
Nakstad 2020	31926258	NSE increases after 24 h and exceeds 60 mg/L	24h and 48h	Not clearly stated	CPC 3-5 at 6 months	69	0.51	FPR 0.13 (0.01-0.53)	Moderate	Study confounding moderate

Neuron Specific Enolase (NSE) as a predictor of functional outcome

Nakstad 2020	31926258	NSE increases after 24 h and exceeds 80 mg/L	24h and 48h	Not clearly stated	CPC 3-5 at 6 months	50	0.51	FPR 0 (0)	Moderate	Study confounding moderate
Deye 2020	32858156	NSE at 48 hours, criterion 38.3 mcg/L	48 hours from ROSC	Continuous	CPC 3-5 at 3 months	330	0.67	Sensitivity 92.3%, Specificity 59.6%, AUC 0.776 [95%CI 0.716-0.837]	High	Self fulfilling prophecy high

Neuron Specific Enolase (NSE) as a predictor of functional outcome

Rafecas 2020	30935900	Delta NSE>=1 (increase, not decrease)	The best OHCA-NSE1 time interval appeared to be 18 to 24 hours (AUC 0.9389, 95% CI, 0.8692-1.000, including 38 patients). The best OHCA-NSE2 time interval appeared to be 69 to 77 hours (AUC 0.9910, 95%, CI 0.9657-1.000 with 21 patients). There was, however, remarkable overlap of CI in all assessed time intervals.	0.35	CPC 3-5 at 6 months	150	0.41	Positive Delta-NSE had a sensitivity of 63.8% for predicting CPC 3-5 and negative Delta-NSE had a specificity of 86.5% for CPC 1-2; positive predictive value was 77.2% and negative predictive value was 76.9%. As continuous variable, OR 1.016 (95% CI, 1.008-1.024), P < .001, for 1% increase. This was also true for Delta-NSE as a binary variable: OR 11.58 (95% CI, 4.89-27.41), P < .001. Among patients with a high first NSE > 33 ng/mL, 32 of 33 patients with CPC 3-5 had increasing NSE. 60.0% of patients with NSE1 > 33 ng/mL and decreasing NSE had good prognosis (CPC 1-2)	High	Self fulfilling prophecy moderate
Oh 2019	31215475	Peak NSE > 41.7 ng/mL	Immediately after ROSC and repeated 24, 48, and 72 h later.	0.59	CPC 3-5 at 6 months	160	0.70	Sensitivity 81.3% (95%CI 72.8–88.0), Specificity 91.7% (95%CI 80.0–97.7). AUC of NSE 0.91 (95% CI, 0.86–0.95)	Moderate	Study participation moderate, Study confounding moderate

Neuron Specific Enolase (NSE) as a predictor of functional outcome

Oh 2019	31215475	Peak NSE > 68.49 ng/mL	Immediately after ROSC and repeated 24, 48, and 72 h later.	0.43	CPC 3-5 at 6 months	160	0.70	Sensitivity 60.7% (95%CI 51.0–69.8), Specificity 100% (95% CI 92.6–100.0). AUC of NSE 0.91 (95% CI, 0.86–0.95)	Moderate	Study participatio n moderate, Study confounding moderate
Devaux 2016	27438111	NSE (both)	48 h after ROSC	continuo us	CPC 3-5 at 6 months	579	0.53	OR 37.47 (5.16-271.9)	Moderate	Study participatio n moderate, Self-fulfilling prophecy moderate. TTM trial substudy.
Stammet 2015	25975474	serum NSE at 24	24, 48, 72h post ROSC	continuo us	CPC 3-5 at 6 months	686	0.49	OR 0.97 (0.95-0.99)	Moderate	Study participatio n moderate, Self fulfilling prophecy moderate. TTM trial substudy.
Stammet 2015	25975474	serum NSE at 48	24, 48, 72h post ROSC	continuo us	CPC 3-5 at 6 months	686	0.49	OR 1.04 (1.01-1.07). Cutoff of 33 ng/ml at 48 h yielded Specificity 91% and Sensitivity 65%	Moderate	Study participatio n moderate, Self fulfilling prophecy moderate. TTM trial substudy.

Neuron Specific Enolase (NSE) as a predictor of functional outcome

Stammet 2015	25975474	serum NSE at 72h	24, 48, 72h post ROSC	continuous	CPC 3-5 at 6 months	686	0.49	OR 1.07 (1.033-1.105)	Moderate	Study participation moderate, Self fulfilling prophecy moderate. TTM trial substudy.
Reisinger 2007	17060343	Peak NSE for persistent coma poor outcome at 6 mo (CPC 4)	highest during the first 4 days	continuous	CPC 4 at 6 months	177	0.33	OR 1.07 (1.04-1.11)	Moderate	Study confounding moderate, Self-fulfilling prophecy moderate
Moseby-Knappe 2021	34417831	NSE	24, 48, 72h post ROSC	Continuous	CPC 3-5 at 6 months	717	0.50	NSE 24h: sensitivity 85% (80.7–88.5) specificity 46.4% (41.1–51.8); 48h: sensitivity 83.6% (78.9–87.4) specificity 57.5% (52.3–63); 72h: sensitivity 80.4% (75.2–84.7) specificity 74.9% (69.8–79.4)	Moderate	Study participation moderate, Study confounding moderate, Self-fulfilling prophecy moderate. TTM trial substudy.
Zellner 2013	23528678	NSE	Admission, day 1, day 2	Continuous	CPC 3-5 at 6 months	123	0.5	NSE >33 day 1-2: Sensitivity 84% (71–92), Specificity 76% (63–86)	Moderate	Study confounding moderate, Self fulfilling prophecy moderate

Neuron Specific Enolase (NSE) as a predictor of mortality

FIRST AUTHOR LAST NAME	PMID	PROGNOSTIC FACTOR(S) EVALUATED	Timing of prognostic factor evaluation	Prevalence % of the predictor	OUTCOME	Sample size for the outcome	Prevalence % of the outcome	EFFECT SIZE WITH 95% CI as reported in the study (False Positive Rate, Sensitivity, Specificity, Odds Ratio, Relative Risk)	OVERALL RISK OF BIAS FOR THE STUDY	Overall risk of bias: Comments
Luescher 2019	31306716	NSE	72h from ROSC	Continuous	Mortality in-hospital, all-cause	336	0.46	AUC 0.88 (95%CI 0.83–0.94). NSE >33: Sensitivity 83% (72–91); Specificity 73% (64–81); PPV 64% (54–74); NPV 88% (79–93). NSE>47.8: Sensitivity 79% (67.1–87.5); Specificity 85% (77–91); PPV 75% (64–85); NPV 87% (79–92). NSE on days 0, 1, 2, 5 or 7 were less predictive and delta NSE between any 2 time points was not superior.	High	Self fulfilling prophecy high

Neuron Specific Enolase (NSE) as a predictor of mortality

Devaux 2016	27438111	NSE	48 h after ROSC	continuous	mortality 6 months	579	Not available	OR 1.37 (1.23-1.53)	Moderate	Study participation moderate, Self-fulfilling prophecy moderate. TTM trial substudy.
Grubb 2007	17502328	NSE	24-48 hrs post arrest	continuous	in-hospital mortality	133	0.58	Statistically significant in multivariate analysis, OR not stated. AUC 0.81, 95% CI not stated	High	Statistical analysis moderate, Self fulfilling prophecy high

Clinical prediction models- Functional outcome

FIRST AUTHOR LAST NAME	PMID	OHCA/ IHCA/ Both	PROGNOSTIC MODEL(S) EVALUATED	OUTCOME	Sample size for the outcome of interest	Proportion of patients with the outcome of interest	Discrimination: C-statistic/ Area under the curve with 95% CI	Calibration: Predicted to observed, Hosmer-Lemeshow or other, or enter "Not reported"	Any other reported effect size (Odds Ratio, 95% CI etc)- enter as comment	Development vs Validation of model	External validation study?	Overall risk of bias	Comments: Overall Risk of Bias	Overall concern about applicability
Velly 2018	29500154	OHCA and IHCA	OHCA	CPC 3-5 at 6 months	150	0.78	Cutoff ≥ 58 AUC 0·57 (0·46–0·68)		Specificity 100% (89–100), Sensitivity 2% (0–6), PPV 100% (16–100), NPV 22% (16–30)	Both	Yes	UNCLEAR		LOW
Song 2021	33922191	OHCA	OHCA	CPC 3-5 at 3 months	106	0.58	0.86 (0.78–0.92)		Sensitivity 25% (14.7–37.9), Specificity 100% (92–100)	Validation	Yes	UNCLEAR	Analysis unclear, Self-fulfilling prophecy unclear	LOW
Song 2021	33922191	OHCA	CAHP	CPC 3-5 at 3 months	106	0.58	0.80 (0.71–0.87)		Sensitivity 5% (1.0–13.9), Specificity 100% (92-100)	Validation	Yes	UNCLEAR	Analysis unclear, Self-fulfilling prophecy unclear	LOW

Clinical prediction models- Mortality

FIRST AUTHOR LAST NAME	PMID	OHCA/ IHCA/ Both	PROGNOSTIC MODEL(S) EVALUATED	OUTCOME	Sample size for the outcome of interest	Proportion of patients with the outcome of interest	Discrimination: C-statistic/ Area under the curve with 95% CI	Calibration: Predicted to observed, Hosmer-Lemeshow or other, or enter "Not reported"	Any other reported effect size (Odds Ratio, 95% CI etc)- enter as comment	Development vs Validation of model	External validation study?	Overall risk of bias	Comments: Overall Risk of Bias	Overall concern about applicability
Isenschmid 2019	30391369	OHCA and unwitnessed IHCA	OHCA	Mortality, in-house, all-cause	349	0.49	AUC 0.80 (95%CI 0.75-0.85)	Goodness of fit with no evidence of miscalibration, p=0.1	Sensitivity/ Specificity/ PPV/ NPV% for different cutoffs: 20-81/64/68/78, 40-41/94/87/63, 60-2/100/100/52, Youden index 27-67/80/76/72	Validation	Yes	UNCLEAR	Self fulfilling prophecy high	LOW
Isenschmid 2019	30391369	OHCA and unwitnessed IHCA	CAHP	Mortality, in-house, all-cause	349	0.49	AUC 0.84 (95%CI 0.79-0.88)	Goodness of fit with no evidence of miscalibration, p=0.76	Sensitivity/ Specificity/ PPV/ NPV% for different cutoffs: >150-82/72/73/81, >200-39/91/81/61, Youden index 161-77/81/81/77	Validation	Yes	HIGH		LOW

Clinical prediction models- Mortality

Luescher 2019	31306716	OHCA and unwitnessed IHCA	OHCA	Mortality, in-house, all-cause	336	0.46	AUC 0.79, no 95% CI		Adding NSE day 3 levels improved AUC to 0.89. Adding NSE day 3 to OHCA resulted in a Net Reclassification Index (NRI) of 0.64 ($p < 0.001$) for OHCA (among patients with poor outcome, adding NSE increased the risk in the statistical model in 44%, while decreasing the risk in 17%; and among patients with favorable outcome, adding NSE decreased the risk of the model in 52% while increasing it in 15%. Adding NSE day 3 to OHCA resulted in an integrated Discrimination Index (IDI) of 0.18 ($p<0.001$).	Validation	Yes	UNCLEAR	Self fulfilling prophecy high	LOW

Clinical prediction models- Mortality

Luescher 2019	31306716	OHCA and unwitnessed IHCA	CAHP	Mortality, in-house, all-cause	336	0.46	AUC 0.81, no 95% CI		Adding NSE day 3 levels improved AUC to 0.91. Adding NSE day 3 to OHCA resulted in a Net Reclassification Index (NRI) of 0.75 (p < 0.001) for OHCA (among patients with poor outcome, adding NSE increased the risk in the statistical model in 47%, while decreasing the risk in 17%; and among patients with favorable outcome, adding NSE decreased the risk of the model in 59% while increasing it in 14%. Adding NSE day 3 to CAHP resulted in an integrated Discrimination Index (IDI) of 0.25 (p<0.001).	Validation	Yes	HIGH	Self fulfilling prophecy ROB high	LOW
Rubins 2019	31512185	IHCA	GOFAR	Survival to discharge	403	0.19			GO-FAR >= 24 (Very low survival)- 3/60 (5%) survived; 14–23 (Low survival)- 5/90 (5.5%) survived.	Validation	Yes	UNCLEAR	Analysis unclear, Self fulfilling prophecy unclear	LOW

Clinical prediction models- Mortality

Hunziker 2011	21494106	OHCA	OHCA	Mortality at discharge	128	0.77	0.85 (95%CI 0.78-0.91)	Hosmer-Lemeshow C p=0.4		Validation	Yes	UNCLEAR	Self-fulfilling prophecy unclear	LOW
